# The CCR4-NOT complex contributes to repression of Major Histocompatibility Complex class II transcription

**DOI:** 10.1038/s41598-017-03708-7

**Published:** 2017-06-14

**Authors:** Alfonso Rodríguez-Gil, Olesja Ritter, Vera V. Saul, Jochen Wilhelm, Chen-Yuan Yang, Rudolf Grosschedl, Yumiko Imai, Keiji Kuba, Michael Kracht, M. Lienhard Schmitz

**Affiliations:** 1 Institute of Biochemistry, Medical Faculty, Justus-Liebig-University, Member of the German Center for Lung Research, D-35392 Giessen, Germany; 2 Department of Pathology, Universities of Giessen and Marburg Lung Center, Excellence Cluster Cardio-Pulmonary System, D-35392 Giessen, Germany; 3 Max-Planck Institute for Immunobiology and Epigenetics, D-79108 Freiburg, Germany; 4 Department of Biochemistry and Metabolic Sciences, Akita University Graduate School of Medicine, Akita, 010-8543 Japan; 5 Rudolf-Buchheim-Institute of Pharmacology, Justus-Liebig-University, Member of the German Center for Lung Research, D-35392 Giessen, Germany

## Abstract

The multi-subunit CCR4 (carbon catabolite repressor 4)-NOT (Negative on TATA) complex serves as a central coordinator of all different steps of eukaryotic gene expression. Here we performed a systematic and comparative analysis of cells where the CCR4-NOT subunits CNOT1, CNOT2 or CNOT3 were individually downregulated using doxycycline-inducible shRNAs. Microarray experiments showed that downregulation of either CNOT subunit resulted in elevated expression of major histocompatibility complex class II (MHC II) genes which are found in a gene cluster on chromosome 6. Increased expression of MHC II genes after knock-down or knock-out of either CNOT subunit was seen in a variety of cell systems and also in naïve macrophages from CNOT3 conditional knock-out mice. CNOT2-mediated repression of MHC II genes occurred also in the absence of the master regulator class II transactivator (CIITA) and did not cause detectable changes of the chromatin structure at the chromosomal MHC II locus. CNOT2 downregulation resulted in an increased *de novo* transcription of mRNAs whereas tethering of CNOT2 to a regulatory region governing MHC II expression resulted in diminished transcription. These results expand the known repertoire of CCR4-NOT members for immune regulation and identify CNOT proteins as a novel group of corepressors restricting class II expression.

## Introduction

Eukaryotic gene expression is a multi-step process that is regulated at all levels from chromatin accessibility to the various steps of transcription, mRNA splicing, nuclear export, mRNA decay and protein translation^1^. One important regulator controlling all different levels of gene expression is the multi-subunit CCR4-NOT complex. This protein complex is evolutionary conserved in eukaryotes and consists of enzymatically active subunits as well as scaffolding proteins^2^. The integrity of the entire complex depends on the catalytically inactive scaffold proteins CNOT1, CNOT2 and CNOT3^2, 3^. Eukaryotic cells contain differentially composed CCR4-NOT complexes, but the dynamics and regulation of this structural diversity is not understood^2^. The four deadenylases (CNOT6, CNOT6L, CNOT7 and CNOT8) are not bound simultaneously and associate with the complex in a mutually exclusive fashion, whereas CNOT4 associates only transiently with the CCR4-NOT complex^4^.

The physiological roles of the CCR4-NOT complex have been extensively studied in yeast where deletion of *NOT1* results in lethality, while deletion of *NOT2*, *NOT4*, or *NOT5* leads to a slow growth phenotype^5^. The physiological function of CCR4-NOT complex proteins were also studied in vertebrates which surprisingly showed a variety of tissue- or organ-specific defects. Deletion of the *Cnot7* gene in mice causes male sterility due to defects in spermatogenesis and teratozoospermia^6, 7^. While deletion of both *Cnot3* alleles is embryonically lethal, heterodeficient *Cnot3* mice show cardiac dysfunction, increased osteoporosis and increased hepatic expression of mRNAs encoding catabolic enzymes^8–10^. A conditional *Cnot3* knock-out results in a block of early B-cell differentiation, which is associated with an impaired autoregulation of the transcription factor EBF1 or p53^11, 12^.

Components of the CCR4-NOT complex are found in the nucleus where they localize at promoters and bind to nascent RNA transcripts, but also in the cytoplasm where they regulate mRNA metabolism and translation from ribosomes^13–16^. At the chromatin level, deletion of *NOT4* or *NOT5* genes leads to impaired acetylation of histones H3 and H4 and diminished tri-methylation of H3K4 in yeast^17–19^. Gene regulation by CCR4-NOT subunits may involve interaction with specific transcription factors such as retinoic acid X receptor or c-Myb and AP-1^13, 20^. In addition, CCR4-NOT proteins can also interact with general components of the transcription machinery. For example, the yeast CNOT1 subunit binds to the TBP-associated factor yTAF while the CCR4-NOT complex increases the incorporation of TFIIS into elongation complexes^21, 22^. The function of CCR4-NOT proteins for transcriptional repression is also reflected by the name giving abbreviation “negative on TATA” for the NOT proteins^23^.

An example for a transcriptionally repressed gene cluster is provided by the MHC II genes. These genes are typically only expressed in thymic epithelial cells (TECs) and professional antigen-presenting cells (APCs) of the immune system such as dendritic cells, B cells and activated macrophages^24^. APCs take up antigen through various mechanisms to allow subsequent processing and loading of the digestion products onto MHC II proteins for presentation to CD4^+^ T-cells^25^. MHC II-mediated antigen presentation is relevant for protective immune responses against invading pathogens and for the maintenance of self-tolerance. The genes encoded in the MHC II region are found in a 0.9 Mb region on chromosome 6p21 and include “classical” (HLA-DP, HLA-DQ and HLA-DR) and “non-classical” genes (HLA-DM and HLA-DO)^26, 27^. MHC II expression is either completely repressed, fully active or amenable for upregulation in response to cytokines such as interferon γ (IFNγ) in endothelial cells and dermal fibroblasts^28^. Transcriptional regulation of MHC II expression is mediated by a regulatory module consisting of four sequence motifs (the S, X, X2 and Y boxes) which are found upstream of the transcription start site in all MHC II genes^29, 30^. Genetic and biochemical studies allowed the identification of four key trans-acting factors that regulate MHC II gene transcription by interacting with the SXY module: Regulatory Factor X 5 (RFX5), RFX-associated protein (RFXAP), RFX-associated ankyrin-containing protein (RFXANK) and, most importantly, CIITA^31–34^. Inappropriate expression of MHC II genes or mutation of genes encoded in the MHC II locus can be causative for a remarkable number of inflammatory, infectious or autoimmune diseases^35^. Also a number of tumor cells, such as primary mediastinal large B Cell lymphomas, display CIITA mutations which result in diminished MHC II expression to allow immune escape of tumor cells^36^. Pathophysiologically relevant downregulation of MHC II expression is also mediated by the Epstein-Barr virus encoded BDLF3 protein to disable the immune system of the infected host^37^.

Here we performed a systematic and comparative analysis of target genes for the CCR4-NOT subunits CNOT1, CNOT2 and CNOT3. We discovered that downregulation of each CCR4-NOT subunit resulted in a CIITA-independent upregulation of MHC II genes by transcriptional derepression. We thus identify another important regulator ensuring the shut-down of MHC II expression in cells not constitutively expressing these genes.

## Results

### Characterization of CCR4-NOT-dependent gene expression

**Figure 1:**
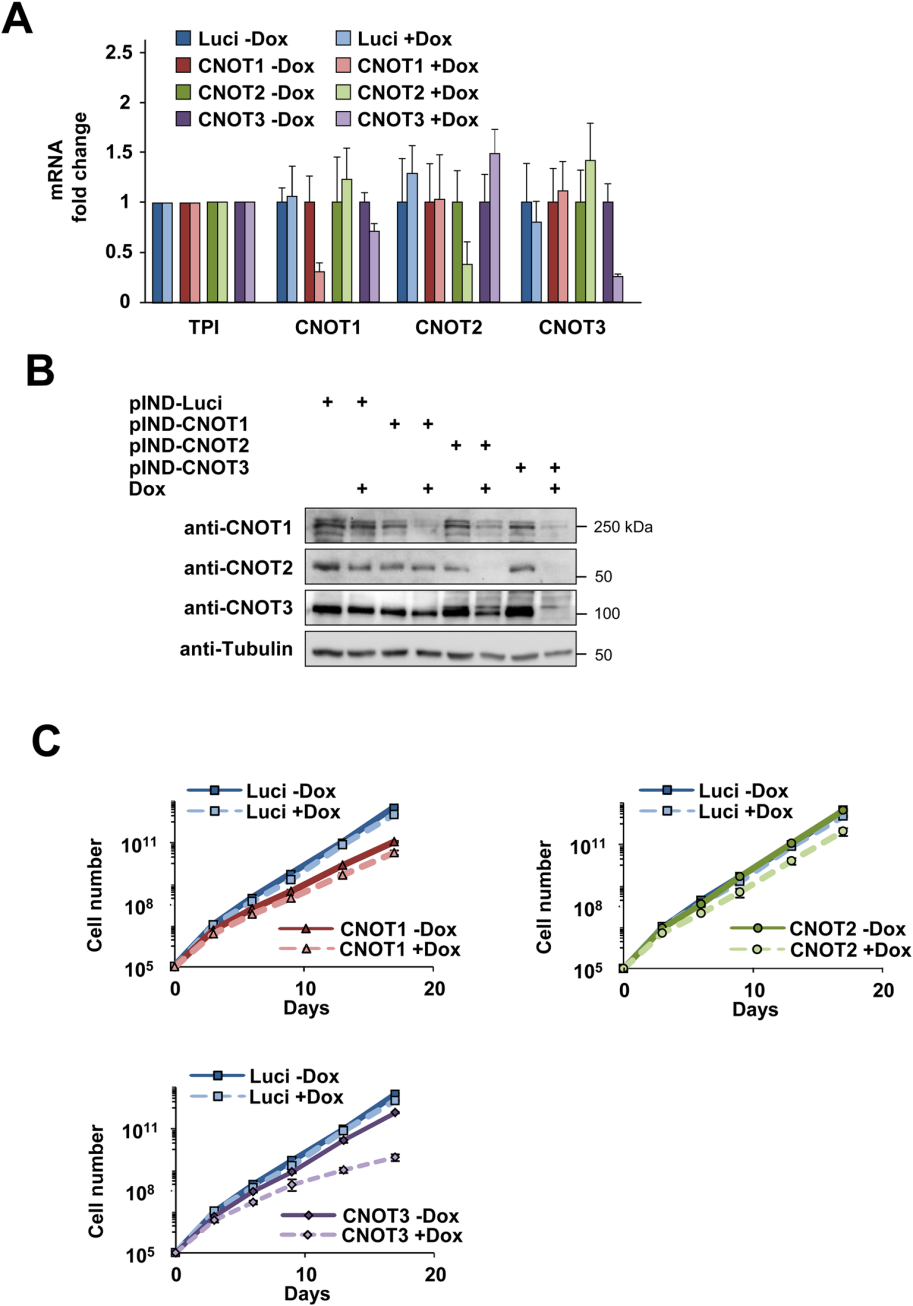
Characterization of human HEK-293T cells allowing Dox-induced downregulation of CNOT subunits. (**A**) Cell clones with a stably integrated pINDUCER plasmid allowing Dox-inducible downregulation of CNOT1, CNOT2 or CNOT3 were treated for 4 days with Dox (1 μg/ml) as shown and analyzed for CNOT mRNA levels by RT-qPCR. Values were normalized to the housekeeping gene TPI (triosephosphate isomerase) which was arbitrarily set as 1 and relative expression levels were determined using the ΔΔCt method. Error bars show standard deviations from two independent experiments performed in triplicates. (**B**) Cell lysates from cells treated as described in (**A**) were prepared and equal amounts of proteins were analyzed by Western blotting for the expression of CNOT1-3 and tubulin as a loading control as shown. The positions of molecular weight markers are indicated. (**C**) The indicated cells were treated with DOX or left untreated as shown, followed by the determination of cell numbers. Growth curves are shown, error bars represent the standard deviation of three biological replicates.

In order to compare the cellular functions of CNOT1, CNOT2 and CNOT3 in a systematic manner, HEK-293T cells allowing the individual knock-down of each subunit were produced. This was achieved by using the pINDUCER system that allows for doxycycline (Dox)-inducible expression of miR30-based shRNAs^38^. This system circumvents potential cellular adaptations to permanent knock-outs and enables a efficient and highly reproducible knock-down. HEK-293T cells were transfected with pINDUCER (pIND) plasmids encoding the individual shRNAs with specificity for CNOT1, CNOT2, CNOT3 or a target sequence against firefly luciferase (Luci) as a control. Stable cell clones were treated with Dox to allow specific downregulation of the individual components of the CCR4-NOT complex as assessed at the mRNA level (Fig. 1A). Western blotting confirmed the efficient knock-down of the individual CNOT subunits at the protein level (Fig. 1B). While the knock-down efficiency was higher for CNOT2 and CNOT3, the reduction in CNOT1 levels were less pronounced, which might be explained by the fact that CNOT1 is the only essential subunit of the complex in yeast^5^. Interestingly, the downregulation of CNOT2 or CNOT3 also impaired protein expression of the other subunits. This crossregulatory effect reflects the commonly observed phenomenon that loss of one component of the CCR4-NOT complex results in the destabilization of further complex members^39–43^. To characterize these cells in more detail, we measured the impact of knock-down of the respective CNOT components on cell proliferation. The CNOT1 knock-down remained without consequences for cell proliferation, which might be due to the limited knock-down efficiency. On the other hand, the prolonged knock-down of CNOT2 and CNOT3 significantly impaired cell proliferation (Fig. 1C). The knock-down did not reduce cell viability (data not shown) or affect the cell cycle profile (Supplementary Fig. 1), thus enabling further functional characterization.

**Figure 2:**
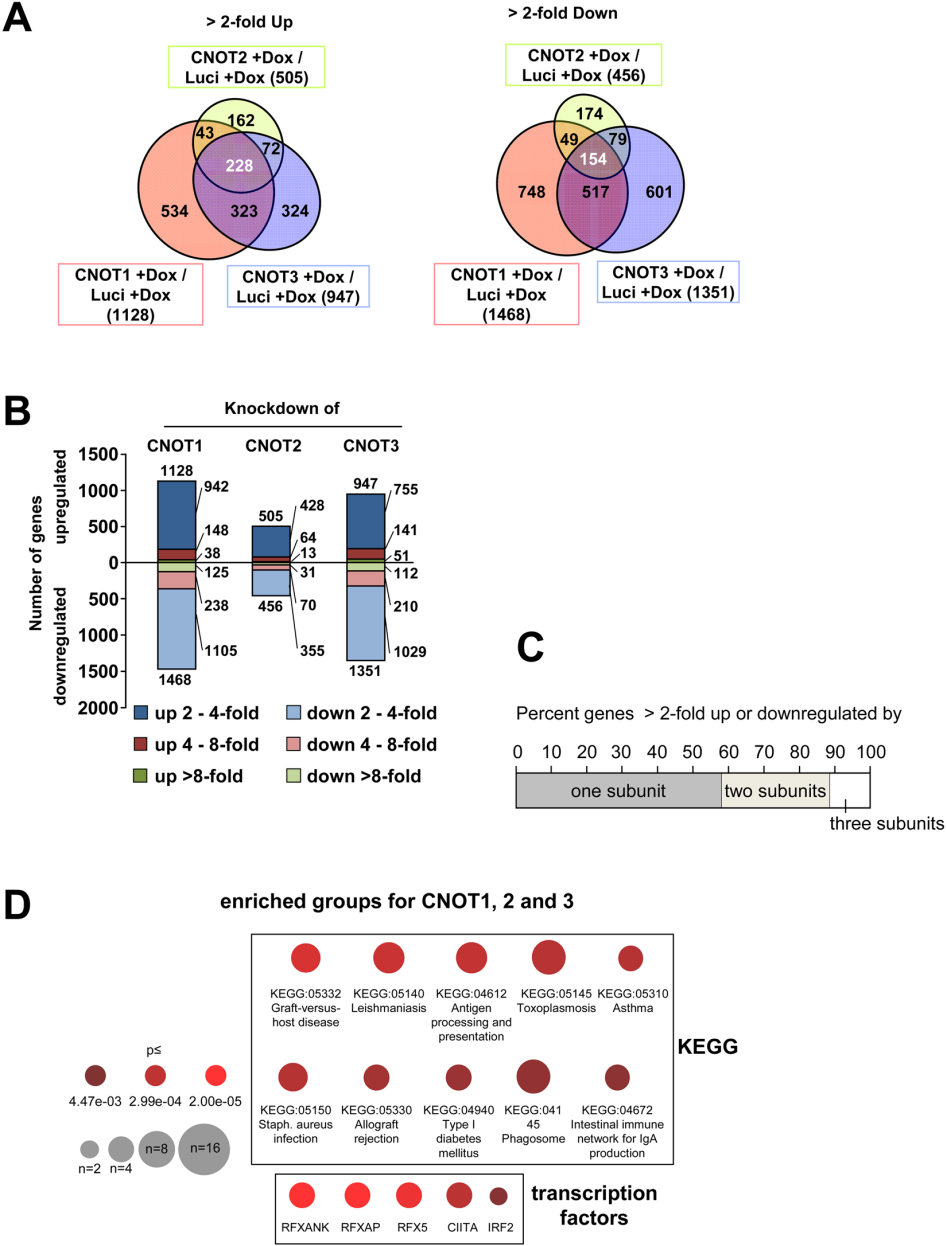
Analysis of CNOT1-3-dependent gene expression. (**A**) Cells were treated for 4 days with Dox and extracted RNA was used to determine transcriptomes using Agilent microarrays. CNOT-dependent genes were identified by comparing gene expression between pIND CNOT + Dox (lacking the respective CNOT subunits) versus pIND Luci + Dox (containing the respective CNOT subunits) to eliminate gene regulatory events attributable to Dox treatment. Venn diagrams show the distribution of genes upregulated or downregulated >2-fold, only genes with significant hybridization signals for all three conditions after quality control filtering are considered. (**B**) The genes identified in (**A**) were grouped according to the magnitude in gene expression change. (**C**) The graph shows the percentage of genes up- or downregulated >2-fold by one, two or the three CNOT subunits, the same filters as in A were applied. (**D**) Upper: All CNOT-dependent genes regulated >2-fold in either direction were subjected to KEGG pathway analysis of regulated gene sets as determined by a Fisher’s exact test for overrepresented groups. Lower: The potential transcription factors mediating the changes in the regulated genes were revealed using the TRRUST database. The size of the circle represents the number of genes in the group and the color represents the p-value of a Fisher’s exact Test.

We then performed DNA microarrays to identify CNOT-regulated changes in gene expression. To analyze the overlap between all three conditions, we discarded those genes which, based on quality filtering, did not have expression values for all three knock-downs. To reveal genes specifically regulated by CNOT subunits we compared genes affected upon knock-down of a CNOT subunit (pIND CNOT + Dox) with controls also treated with Dox but still containing endogenous CNOT (pIND Luci + Dox). Significantly upregulated genes (>2-fold) were detected for CNOT1 (1128), CNOT2 (505) and CNOT3 (947) (Fig. 2A). 228 genes were upregulated by all three CNOT subunits, indicating their broad relevance for the CCR4-NOT function. The analysis of downregulated genes allowed the identification of 154 commonly regulated genes (Fig. 2A and Supplementary Table 1). We also analyzed the distribution of the fold regulation under these conditions as displayed in Fig. 2B. To reveal a potential redundancy between the individual CNOT proteins, we determined the overlap between all genes (n = 4008) regulated by the individual subunits. The majority of genes (58%) was up- or downregulated >2-fold by only one subunit, while 31% and 11% were co-regulated by two or three subunits, respectively (Fig. 2C). This functional diversity is in agreement with the structural diversity of CCR4-NOT complexes^44^. We then performed an enrichment analysis of functional annotations using KEGG (Kyoto Encyclopedia of Genes and Genomes) pathways. The top 10 regulated KEGG pathways are displayed in Fig. 2D and mostly encompass settings with infectious or immunological relevance. All pathways share the regulation of MHC II genes (Fig. 2D). Detailed information about the pathway corresponding genes are given in Supplementary Table 2. Candidate transcription factors mediating the expression of CNOT-dependent target genes were suggested by the TRRUST (transcriptional regulatory relationships unravelled by sentence-based text-mining) database^45^ and include CIITA as well as members of the RFX transcription factor family (Fig. 2D). Similarly, also the GO (Gene Ontology) analysis of biological processes revealed the relevance of the CCR4-NOT complex for members for MHC II- related processes (Supplementary Fig. 2).

### CCR4-NOT components as regulators of MHC II expression

**Figure 3:**
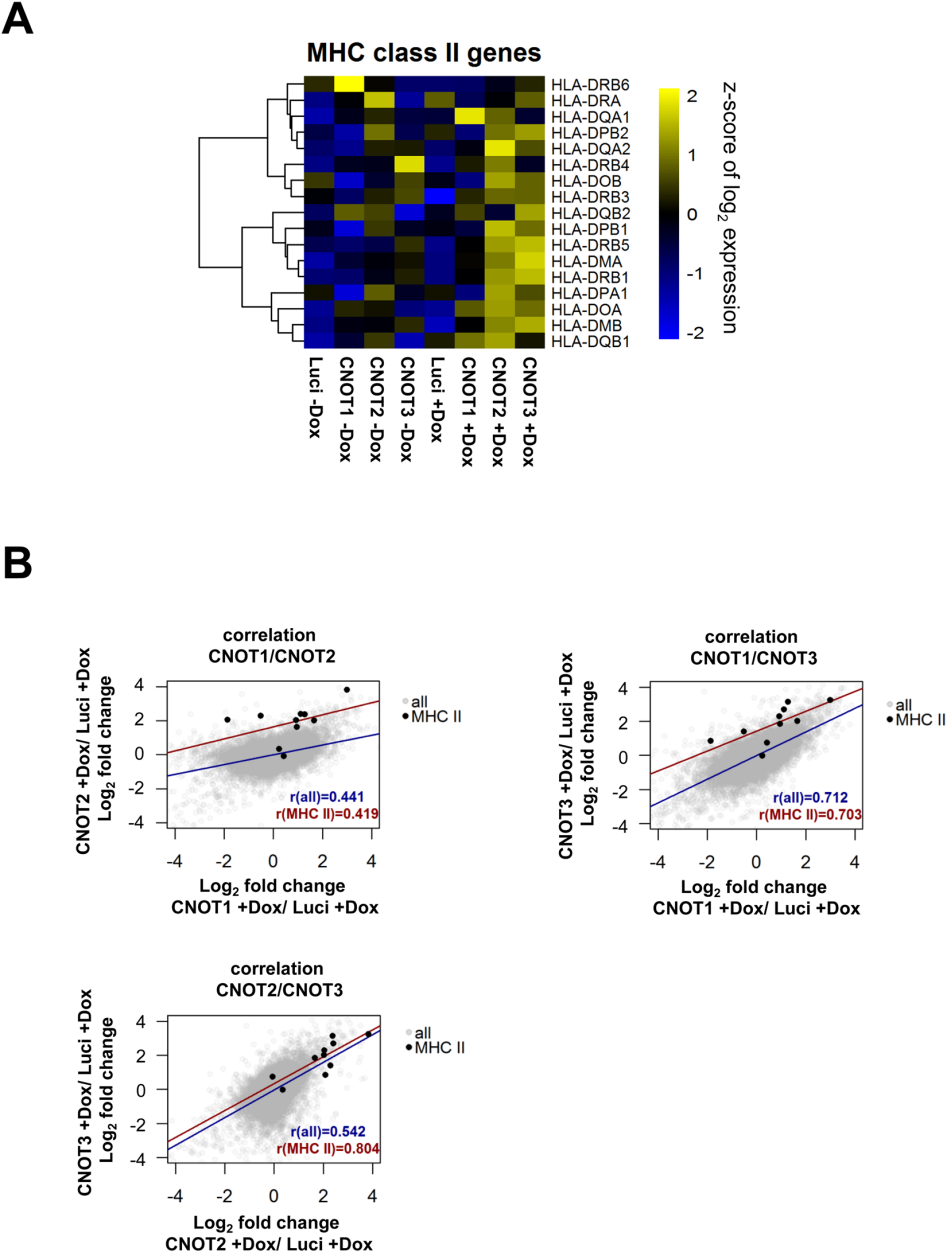
Analysis of CNOT1-3-dependent MHC II gene expression. (**A**) Heat map visualization of MHC II gene expression changes in the different samples as revealed by the microarrays. (**B**) Correlation of the fold changes (in log_2_ scale) in the respective CNOT knock-downs versus the control (pIND Luci + Dox). MHC II genes are marked with black dots. The regression line and r coefficient are shown for the MHC II genes (red) and all data points (blue).

**Figure 4:**
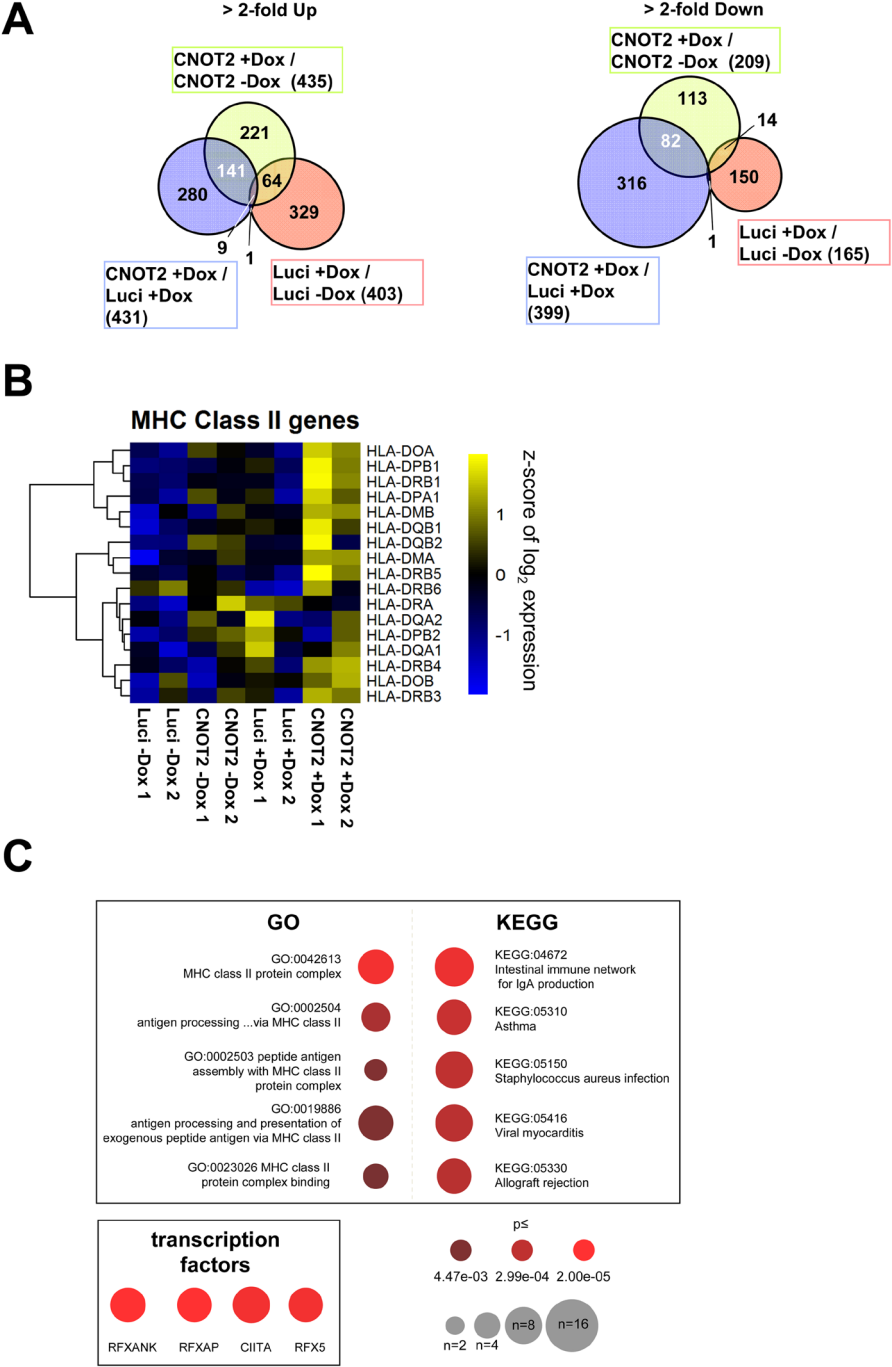
Analysis of CNOT2-dependent transcription programs. (**A**) The 293 T cells with a stably integrated pIND CNOT2 or a pIND Luci control plasmid remained untreated or were treated for 4 days with Dox, followed by analysis of gene expression using Agilent microarrays. The microarray data from the new experiment and from the previous experiment were analysed together using the Limma package of Bioconductor with the R software. Venn diagrams show the overlap of regulated genes more than 2-fold in each of the comparisons indicated. *Bona fide* CNOT2 target genes were identified by simultaneous comparison between pIND CNOT2 + Dox versus pIND CNOT2 -Dox and also between pIND CNOT2 + Dox versus pIND Luci + Dox. This procedure eliminates gene regulatory events attributable to Dox treatment (shown in red) or to the differences in the cell line background. Only genes with significant hybridization signals for all three conditions were considered. (**B**) The heat map visualizes a cluster analysis showing the expression level changes of the MHC class II genes in the different samples hybridized to the microarrays. (**C**) Upper: The 141 upregulated and 82 downregulated CNOT2 target genes identified in (**A**) were analyzed for overrepresentation in the KEGG and GO pathway databases using a Fisher’s exact test, the top 5 pathways are displayed. Lower: Candidate transcription factors mediating the transcriptional changes were revealed by the TRRUST database of transcription factors. The number of regulated genes within the pathways and their statistical relevance are displayed as in Fig. 2D.

**Figure 5:**
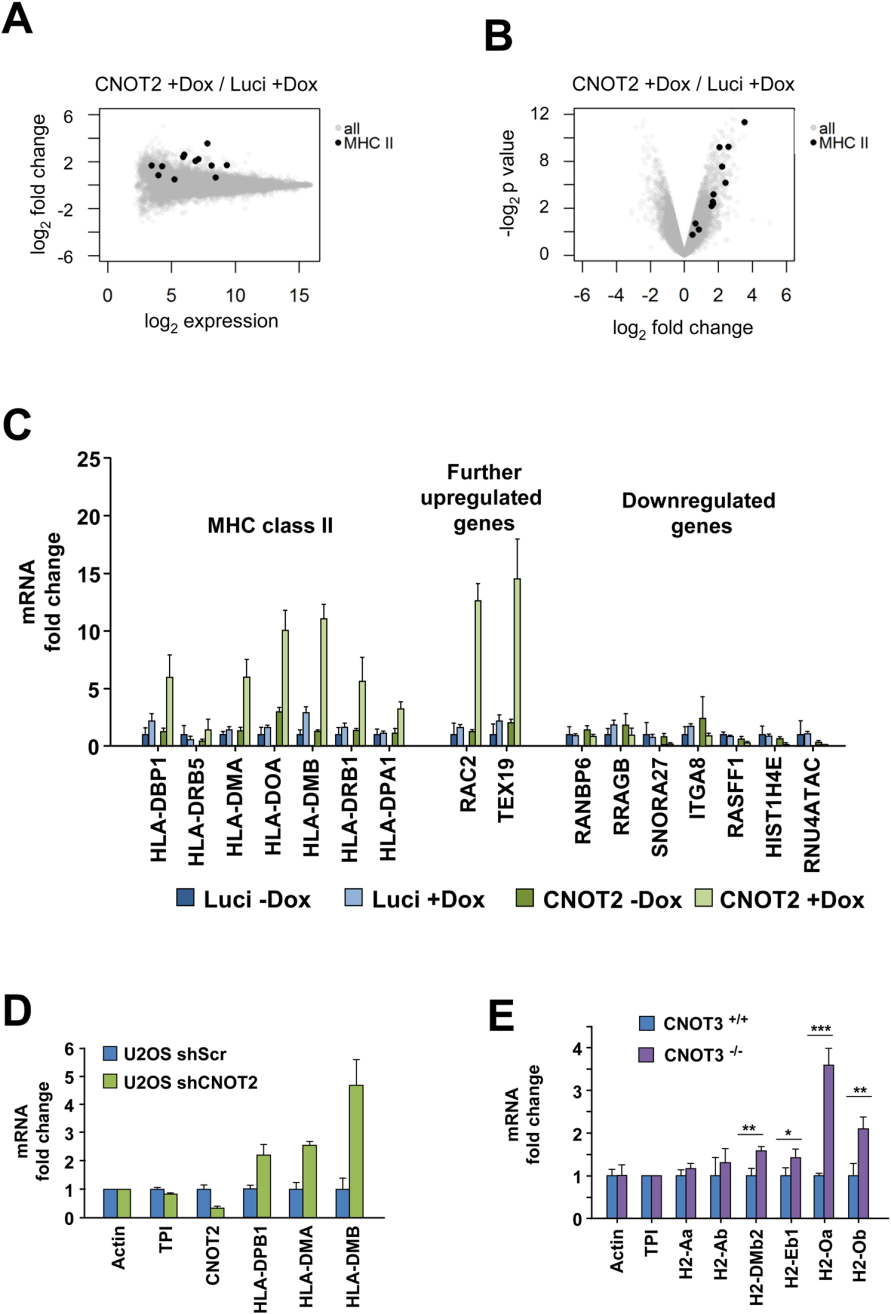
Analysis of CNOT2-dependent MHC II transcription. (**A**) The MA plot depicts log_2_ expression versus log_2_ fold change for all genes (grey) and MHC II genes (black). (**B**) The Volcano plot shows log_2_ fold changes versus log_2_ p-values for all genes and MHC II genes. (**C**) Human HEK-293T pIND Luci and pIND CNOT2 cells were treated for 4 days with Dox to downregulate endogenous CNOT2 and analyzed for expression of the indicated mRNA levels by RT-qPCR. All values were normalized to TPI, the gene expression in control was arbitrarily set as 1 in order to facilitate direct comparison of the data. The error bars represent the standard deviation of three biological replicates. (**D**) Human osteosarcoma U2OS cells were transfected with vectors directing the expression of a CNOT2-specific shRNAs or an unspecific control scrambled shRNA (shScr). Transfected cells were selected for two days in puromycin to eliminate untransfected cells and RT-qPCR was performed to quantitate mRNA levels of some MHC class II genes and to ensure the CNOT2 knockdown. The error bars represent the standard deviation of three biological replicates. (**E**) Mice were treated with tamoxifen to induce the knockout of CNOT3, followed by isolation of naïve macrophages from the bone marrow as specified in Materials and Methods. The mRNA levels of some MHC class II genes were determined by RT-qPCR, the error bars represent the standard deviation from three biological replicates.

The CNOT-dependent regulation of the expression levels of individual MHCII genes was then visualized in a heat map (Fig. 3A). Downregulation of either CNOT2 or CNOT3, and to a lesser extend of CNOT1, led to a consistent upregulation of these genes, as shown when comparing the distribution of the fold change upon CNOT1, 2 or 3 of MHC II members to all the genes in the microarray (Supplementary Fig. 3). The co-regulation of MHC II genes by CNOT2 and CNOT3 was also evident when plotting the fold change of each CNOT subunit depletion against the others for the ten MHC II genes with values for all conditions after filtering (Fig. 3B). This analysis showed the highest correlation coefficient for MHC II regulation between CNOT2 and CNOT3 (r = 0.804) and the second highest between CNOT1 and CNOT3 (r = 0.703). CNOT2 showed the strongest effects on derepression of MHC II genes thus prompting us to investigate the function of this subunit in further detail. A biological and technical replicate of the DNA microarray was performed to allow a statistical analysis of the data (Supplementary Fig. 4) and changes in CNOT2-dependent gene expression of all CNOT2 gene arrays are displayed in Fig. 4A. To eliminate possible effects by the Dox treatment or clonal differences between the various cell lines, we only considered those genes which were regulated upon the CNOT2 downregulation (pIND CNOT2 + Dox) compared with both controls pIND CNOT2 -Dox (to eliminate clonal differences) and pIND-Luci + Dox (to eliminate the effects of Dox treatment). This analysis revealed 141 upregulated and 82 downregulated *bona fide* CNOT2-dependent genes (Fig. 4A). A cluster analysis of the different microarray experiments also showed the consistent and specific upregulation of diverse MHC II genes upon CNOT2 knock-down compared to the various control samples, as well as the reproducibility between the replicate samples (Fig. 4B). The KEGG and GO analysis showed the prevalence of MHC II genes in the groups identified (Fig. 4C and Supplementary Fig. 5), while the candidate transcription factors mediating these effects are RFX transcription factor family members and CIITA. The magnitude of mRNA upregulation of MHC II genes in relation to all expressed genes of these cells was visualized by MA Plots (Fig. 5A). The prominent and statistically significant regulation of MHC II genes was also seen in a volcano plot analysis (Fig. 5B). The CNOT2-dependent regulation of representative MHC II and further target genes was recapitulated by RT-qPCR experiments (Fig. 5C). To reproduce these effects of downregulation of CNOT2 in a different cell line, we transfected cells to express a conventional shRNA and measured MHC II expression in U2OS cells. Also these RT-qPCR experiments revealed increased MHC II expression (Fig. 5D), showing that CNOT2-dependent MHC II regulation is not restricted to specific cell lines. Furthermore, also the abrogation of CNOT2 expression via CRISPR-Cas9 resulted in increased expression of selected MHC II genes (Supplementary Fig. 6). To test the effects of CNOT elimination on MHC II expression *in vivo*, we took advantage from the availability of a Cre recombinase-dependent CNOT3 conditional knock-out mouse model (K. Kuba and Y. Imai, unpublished). After tamoxifen-driven CNOT3 deletion, naïve macrophages and T cells were analyzed for expression of selected MHC II genes by RT-qPCR. Ablation of CNOT3 caused significant upregulation of 4 from 6 analyzed MHC II mouse genes in macrophages (Fig. 5E), but interestingly not in T cells (not shown).

### Mechanism of CNOT-dependent MHC II repression

**Figure 6:**
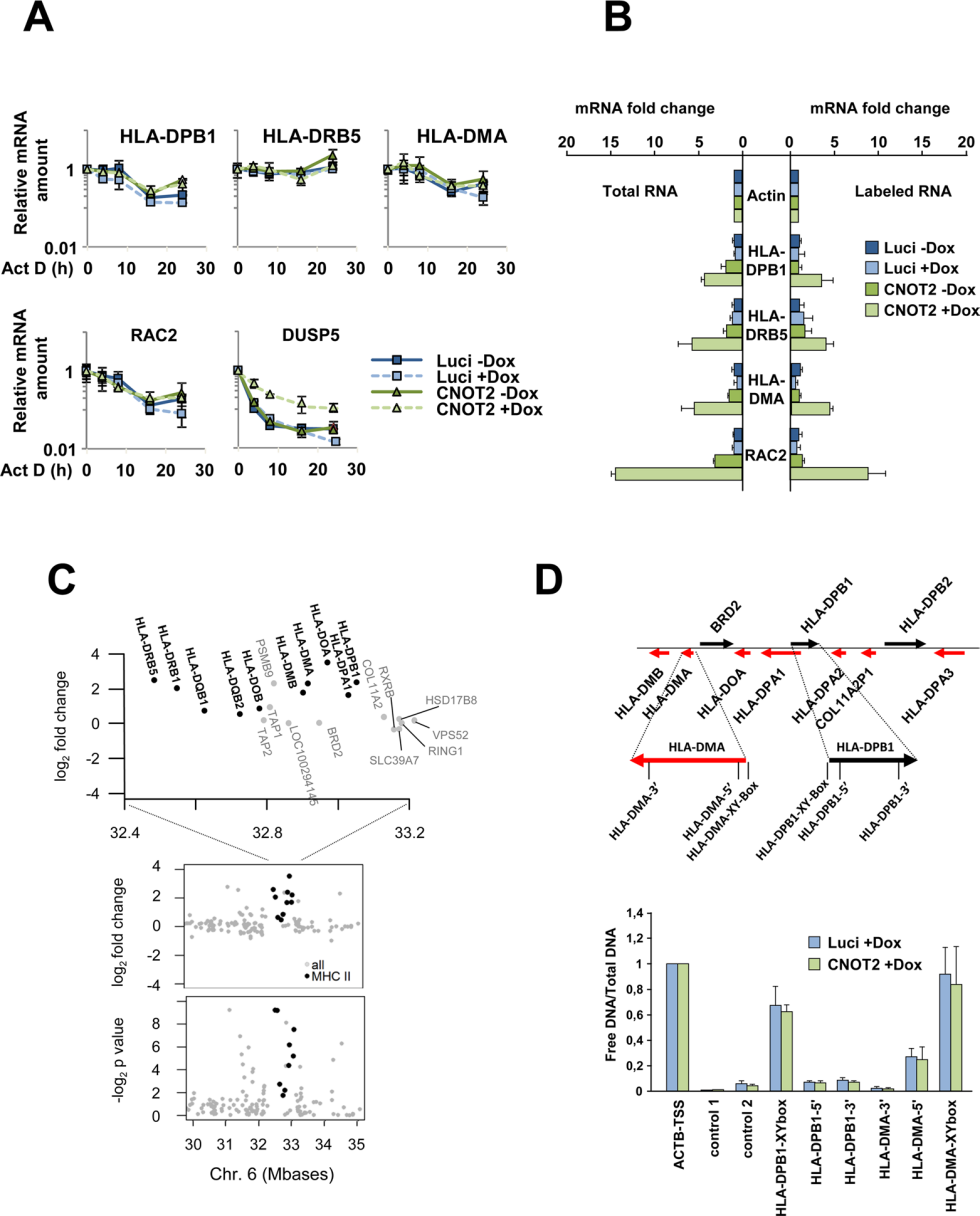
Mechanisms of CNOT2-dependent repression of MHC II transcription. (**A**) HEK-293T pIND Luci or pIND CNOT2 cells were incubated for 4 days in the presence of Dox to downregulate CNOT2. Subsequently the cells were treated with 1 μg/ml of Actinomycin D for the indicated times to block *de novo* transcription and relative mRNA levels of CNOT2 target genes were quantified by RT-qPCR and normalized to the Actin levels. To facilitate comparison, all mRNA levels in the controls not treated with Actinomycin D were set as 1. The error bars represent the standard deviation of three biological replicates. (**B**) HEK-293T pIND Luci or pIND CNOT2 cells were treated for 4 days with Dox to downregulate CNOT2 as shown. Subsequently 4-thiouridine was added for 20 min and RNA was isolated by Trizol extraction. One tenth of the RNA was used for cDNA synthesis and RT-qPCR, while the remaining RNA was biotinylated *in vitro* and purified via Streptavidin-coupled beads as specified in Materials and Methods. The purified RNA represents the *de novo* transcribed fraction which was further analyzed by cDNA synthesis and RT-qPCR. The results show changes in gene expression, error bars represent the standard deviation of three biological replicates. (**C**) The CNOT2-dependent changes at the MHC II locus on chromosome 6 are shown with p-value of the differential expression (lower panel) and as fold changes (upper panels). The position of the loci on chromosome 6 is indicated, the MHC class II genes are highlighted in bold. (**D**) Quantification of free DNA versus chromatinized DNA as determined by FAIRE in the depicted regions of the MHC class II locus. The upper part shows a schematic representation of the locus, the lower part displays the results with error bars representing standard deviations from two biological replicates. The transcription start site for the beta Actin gene (ACTB-TSS) was used as a positive control.

**Figure 7:**
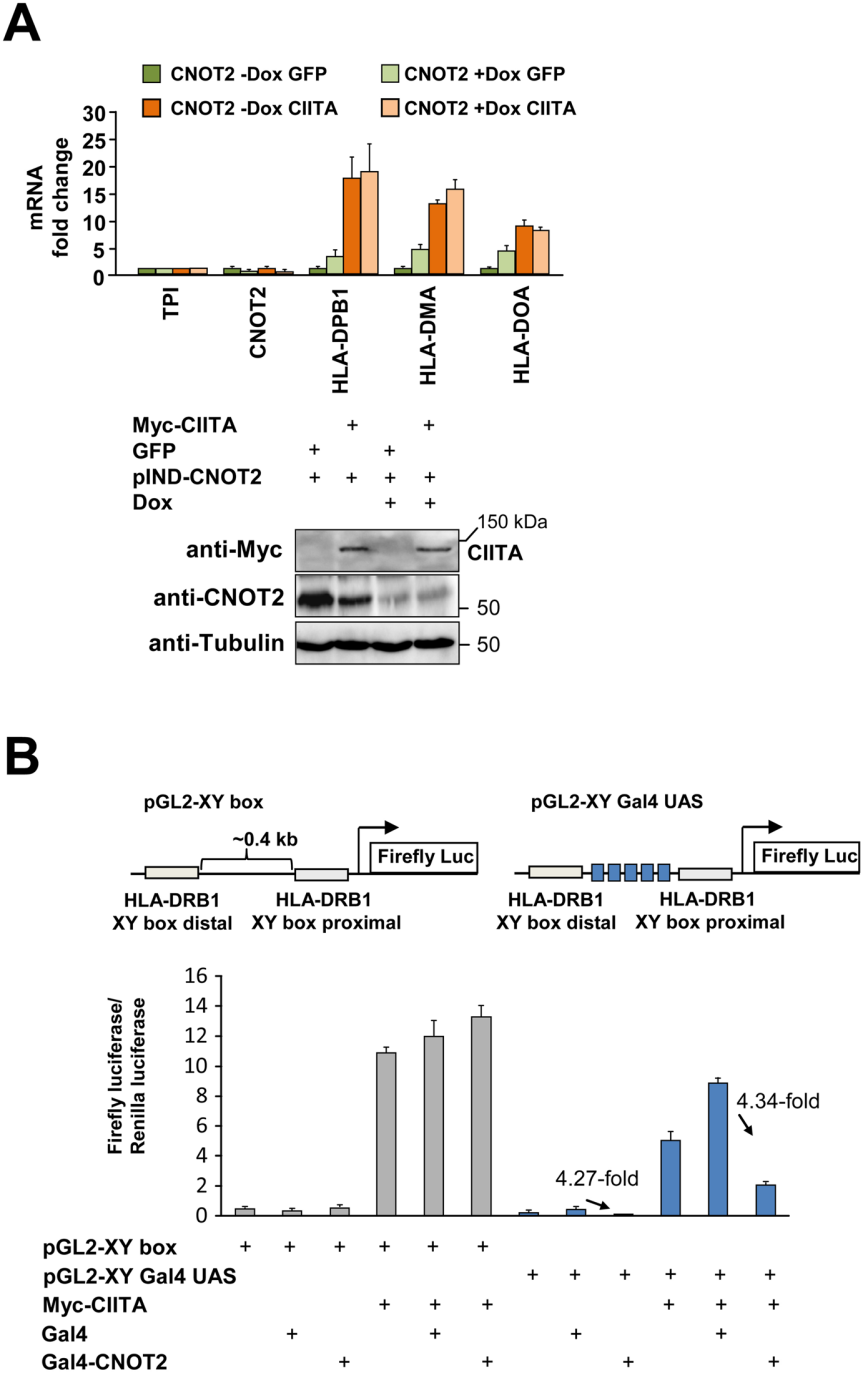
DNA tethering of CNOT2 mediates repression of MHC II transcription. (**A**) The HEK-293T cells with a stably integrated pIND CNOT2 plasmid were transfected to express either CIITA or as a control the GFP protein. Following Dox-mediated knock-down of CNOT2, one part of the cells was lysed for subsequent use by Western blot analysis (lower), while the other part was used to determine expression of endogenous MHC II genes by RT-qPCR. The error bars represent standard deviations from three biological replicates. (**B**) HEK-293T cells were transfected with two different reporter constructs: One construct encompasses the proximal and distal XY boxes from the HLA-DRB1 promoter (left), while the other reporter construct contains additionally 5 binding sites for the yeast Gal4 protein (blue boxes at the right site). These different reporter genes were cotransfected with expression vectors for CIITA, Gal4-CNOT2 or the Gal4 control. The graph shows firefly luciferase activity obtained from transfected plasmids with the indicated constructs, normalized to the *Renilla* luciferase control. Fold repression achieved by CNOT2-Gal4 expression compared to Gal4 alone is indicated in the graph. Error bars represent the standard deviation from three biological replicates.

It was then important to investigate the molecular mechanisms underlying CNOT-mediated regulation of MHC II genes. One obvious possibility is the CCR4-NOT-mediated regulation of mRNA stability, which has previously been described for many other genes^46–49^. To investigate this aspect, we measured mRNA decay rates of MHC II genes after CNOT2 depletion using Actinomycin D, an inhibitor of *de novo* transcription. CNOT2 depletion stabilized the mRNA of the DUSP5 control gene, but did not cause detectable changes in the mRNA stabilities of MHC II genes or RAC2, another CNOT2-dependent gene (Fig. 6A). As CNOT2 depletion did not produce a detectable effect on mRNA stabilities we then tested its impact on *de novo* transcription. To address this issue, freshly synthesized RNA was metabolically labeled using 4-thiouridine, which allows for its *in vitro* biotinylation and subsequent Streptavidin-mediated purification^50^. The comparative analysis of total mRNA and *de novo* synthesized mRNA showed that CNOT2 downregulation largely affects the mRNA synthesis steps (Fig. 6B). These results suggest a repressive role of the CNOT2 complex for the entire MHC II gene cluster on chromosome 6, as displayed in Fig. 6C. To study the potential effects of CNOT2 depletion on the chromatin structure of this locus, we queried the chromatin accessibility using the FAIRE technique^51^. These experiments allowed to detect open chromatin structures at the promoter regions of the MHC II locus, but failed to reveal any CNOT2-dependent alterations for the tested regions (Fig. 6D). The transcription regulator CIITA was identified as a candidate for CNOT2-mediated transcriptional effects (see Fig. 2D) and is a master regulator of gene expression from the MHC II locus^30, 52, 53^. To test its potential relevance for CNOT2-mediated MHC II gene regulation, HEK-293T cells with a stably integrated pIND CNOT2 plasmid were treated with Dox as shown, followed by transfection of either CIITA or the GFP control protein. Overexpression of CIITA caused a robust activation of the endogenous HLA-DPB1, HLA-DMA and HLA-DOA genes, but this stimulatory effect was not further augmented upon CNOT2 depletion (Fig. 7A). The lack of CNOT2-dependent effects on CIITA-driven gene expression was also seen in luciferase reporter assays. Expression of a luciferase reporter plasmid controlled by the CIITA-responsive proximal and distal XY boxes from the HLA-DRB1 gene was also not affected by CNOT2 knock-down (Supplementary Fig. 7). These data suggest that the CCR4-NOT complex does not contribute to expression of the CIITA-activated locus and is rather necessary to maintain the locus in its inactive state in cells not poised for MHC II expression. To directly test the effect of CNOT2 promoter recruitment on gene expression we used a reporter construct containing binding sites for the DNA-binding Gal4 protein between the two XY boxes of the reporter construct to allow tethering of a Gal4-CNOT2 fusion protein. HEK-293T cells were transfected with a luciferase reporter gene containing the XY boxes together with the Gal4 binding sites or a control lacking the Gal4 binding sites along with plasmids encoding CIITA, Gal4 or a Gal4-CNOT2 fusion protein. The analysis of luciferase expression showed that tethering of CNOT2 to the construct repressed transcription (Fig. 7B) both in the presence and absence of CIITA, in line with a repressive role of CNOT2 for *de novo* transcription of MHC II genes.

## Discussion

Here we identify components of the CCR4-NOT complex as novel regulators ensuring repression of the MHC II locus, as revealed by various loss-of-function models in cells and animals. Constitutive expression of MHC II genes is repressed in most cells and mostly restricted to APCs and TECs, while IFNγ triggers its expression also in epithelial, vascular, and connective tissue^24^. Unregulated or poor MHC II expression can cause autoimmune or infectious diseases and cancer, thus adequate regulation of MHC II expression is tightly controlled by a hierarchically organized network at all levels of gene expression^54, 55^. Expression of MHC II genes is also observed in some other physiological situations, for example in a subpopulation of human neural stem cells^56^. Increased expression can be also found in tumors such as hepatocellular carcinoma^57^, although in most cases cancer cells downregulate MHC II expression to avoid an anti-tumoral immune response^36, 58^. Intriguingly, also the expression of MHC class I genes can be restricted by mRNA deadenylation upon tethering of the CCR4-NOT complex via the RNA-binding protein MEX-3C^59^.

The repression of MHC II transcription is mediated by a variety of molecular mechanisms, but the exact target of CNOT-mediated repression remains to be identified. We did not see significant changes of chromatin accessibility at various MHC II loci using FAIRE, which does not exclude the possibility that other genomic loci in this area might show differences in chromatin organization. The repressing function of CNOT2 on transcription from the MHC II locus occurred after Gal4-dependent tethering of this protein. It is currently not clear whether CNOT2 (or CNOT1 and CNOT3) are associated with the DNA or chromatin and whether this is mediated by direct or indirect effects. A direct repression could be achieved by direct constitutive association of CNOT2 with chromatin at the MHC II locus. Such a mechanism has been described for CNOT1 and CNOT2 for repression of hormone-inducible genes, although CNOT2 chromatin recruitment occurred in an estradiol-inducible fashion^13^. Indirect effects of CNOT-mediated transcriptional repression could involve the association or regulation of transcription factors. Examples for such a mechanism are interactions of CNOT1 with estrogen receptor α, CNOT9 binding to c-Myb^20^ and CNOT2 interaction with ERG^60^.

An obvious candidate for CNOT2-mediated effects is the master regulator CIITA, but we do not favor a role of this transcriptional regulator for several reasons: (I) the epithelial cell lines used in this study do not contain significant CIITA levels (data not shown). (II) neither CIITA, nor components of the RFX complex (RFXANK, RFXAP and RFX5) and further coactivators of MHC II expression were upregulated by CNOT2 knock-down (Supplementary Fig. 8). (III) published CIITA target genes^61^ show only a minor overlap with CNOT2 target genes identified here and their expression is not significantly affected upon CNOT2 knock-down (data not shown). (IV) Published genomic binding sites of CIITA^62, 63^ do not control genes that are regulated by CNOT2 (Supplementary Fig. 9). (V) the knock-down of CNOT2 did not augment CIITA-triggered expression of MHC II genes as measured in reporter gene assays and for the endogenous genes. Thus, we anticipate that CNOT2-mediated effects on MHC II expression occur by one of the known CIITA-independent mechanisms^64, 65^. Upregulation of MHC II genes did not occur after knock-out of CNOT3 in T cells (data not shown) or after knock-down of CNOT2 in Raji B cell lymphoma cells (Supplementary Fig. 10). These findings would support a model where cells with high constitutive MHC II expression are not regulated by CNOT proteins, while cells with absent or inducible class II expression including naïve macrophages would be repressed by CNOT proteins^66^. Future studies should reveal the relative contribution of CNOT subunits for the regulation of class II expression in different cell types.

The systematic analysis of gene expression in cells lacking CNOT1, CNOT2 or CNOT3 revealed a set of jointly and individually regulated genes. However, the relative degree of conjointly gene expression cannot be precisely determined, as the elimination of one CCR4-NOT subunit can also lead to the destabilization of further members of this complex. Due to the limited availability of high quality antibodies, we could not determine the consequences of the knock-down of individual CCR4-NOT proteins on all complex members. It would probably not be surprising to see destabilization of additional CCR4-NOT complex members, as the elimination one protein often affects further components of a multi-protein complex^67, 68^. It will thus be relevant to keep this point in mind when interrogating existing knock-out or knock-down models for the CCR4-NOT complex. Even though the exact number of co-regulated genes could not be determined, our data indicate that a substantial fraction of target genes is regulated by individual CNOT proteins. Their function is further modulated by posttranslational modifications such as ubiquitination or phosphorylation^69^. While in yeast cells the CCR4-NOT complex is of relevance for the majority of genes^14^, its role in vertebrates seems to be less global and also controls cell- or organ-specific functions, as seen by the phenotype of existing knock-out animals. This specificity helps to explain the large diversity of cellular effects seen after knock-down of individual CCR4-NOT complex members. These effects range from impaired cell growth after interference with CNOT6L^70^ to reduced cell viability after knock-down of CNOT1 or CNOT2^3, 40^ or defects in mitotic progression after knock-down of CNOT3^71^.

This study also suggests a contribution of the CCR4-NOT complex for the regulation of the immune system. In support to this notion CCR4-NOT proteins also contribute to the regulation of MHC class I gene expression^59^ and the knock-out of mouse *Cnot3* leads to a block in early B cell development^11, 12^. It will thus be interesting to study the function of this multi-protein complex in infection models in the future.

## Materials and Methods

### Cell culture and transfection

Human embryonic kidney (HEK)-293T cells, its derivatives and U2OS cells were cultured in DMEM medium supplemented with 10% FCS, Glutamine, Penicillin and Streptomycin. Raji B cell lymphoma cells were cultured in RPMI medium supplemented with 10% FCS, Glutamine, Penicillin and Streptomycin. Transfection of adherent cells was done using linear polyethylenimine as described^72^. Raji suspension cells were electroporated using a Biorad electroporator. Briefly, 3 × 10^7^ cells were collected by centrifugation, washed with phosphate-buffered saline (PBS) and resuspended in 300 μl of RPMI medium without serum and antibiotics. 10 µg of DNA were mixed with the cells and transferred to a 4 mm electroporation cuvette. Cells were electroporated using an exponential decay pulse at 250 V and 950 μF. Cells were incubated for 10 min at room temperature and then transferred to a culture flask containing 10 ml of complete RPMI medium.

### Plasmids and Primers

This information is given in Supplementary Table 3.

### RNA extraction, RNA labeling, cDNA and RT-qPCR

RNA was extracted using TRIzol and cDNA was synthesized with SuperScript II and oligo-dT18 primers as described by the manufacturer (Invitrogen). RT-qPCR was performed using Thermo Fisher 2X SYBR Green Master Mix in a Applied Biosystems 7300 machine. Metabolic RNA labeling was performed as previously described^50^ with modifications. Briefly, the cell culture medium was supplemented with 200 μM 4-thiouridine (Sigma T4509), and cells were incubated for 20 min at 37 °C. Cells were then washed with PBS and RNA was extracted, followed by biotinylation of 50 µg of RNA by incubation in 500 μl of biotinylation medium (0.2 mg/ml EZ-Link Biotin-HPDP (Pierce, 21341), 10 mM Tris/HCl pH 7.5, 1 mM EDTA) for 1 h at room temperature. RNA was purified by chloroform extraction and precipitated with ethanol. Biotinylated RNA was purified using Streptavidin-coupled Dynabeads (Invitrogen 65001), washed three times with washing buffer (100 mM Tris/HCl, 10 mM EDTA, 1 M NaCl, 0.1% Tween-20) at 65 °C, and three times at room temperature. Elution was performed twice by incubation of labeled RNA for 10 min at room temperature with 100 µl of 100 mM DTT.

### Microarrays

RNA was extracted using the RNeasy kit from Qiagen and total RNA was amplified and Cy3-labeled using the LIRAK kit as described by the provider (Agilent) using 200 ng of total RNA. The Cy3-labeled aRNA was hybridized overnight to Agilent microarrays slides containing 60000 probes covering annotated genes and non-coding RNAs (Agilent Technologies, design ID 028005). Hybridization and subsequent washing and drying of the slides was performed following the Agilent hybridization protocol. The dried slides were scanned at 2 µm/pixel resolution using the InnoScan 900 (Innopsys, Carbonne, France). Image analysis was performed with Mapix 6.5.0 software and calculated values for all spots were saved as GenePix files. The microarray data are deposited in the GEO database GSE90474.

### Luciferase assays

Cells were seeded in 12 well plates and transfected with 100 ng of pCI-neo-*Renilla*-Luciferase as an internal control, 1 μg of the indicated firefly luciferase construct and 1 µg of each of the corresponding overexpression plasmids using linear polyethylenimine. The amount of transfected DNA was kept constant using empty vector where necessary. One day post-transfection the luciferase activity was determined using the DUAL Luciferase assay kit from Promega.

### Protein extraction and Western blotting

Cells were washed once with PBS and resuspended in NP-40 lysis buffer (20 mM Tris/HCl pH 7.5, 150 mM NaCl, 1 mM phenylmethylsulfonylfluoride, 10 mM NaF, 0.5 mM sodium orthovanadate, leupeptin (10 µg/ml), aprotinin (10 µg/ml), 1% (v/v) NP-40 and 10% (v/v) glycerol). After incubation for 20 min on ice, cells were centrifuged for 10 min at 13000 rpm to remove the insoluble fraction and the supernatant was transferred to a new tube. SDS sample buffer was added and the extracts were heated for 5 min at 95 °C, followed by separation of proteins using SDS-PAGE and Western blotting as previously described^73^. The following antibodies were used for immunoblotting: rabbit polyclonal anti-CNOT1 (kind gift of Dr. Elisa Izaurralde), mouse monoclonal (2191C2a) anti-CNOT2 (Santa Cruz), rabbit polyclonal anti-CNOT3 (self-made), mouse monoclonal (9E10) anti-Myc (Santa Cruz) and mouse monoclonal (Tub2.1) anti-Tubulin (Sigma Aldrich).

### Formaldehyde Assisted Isolation of Regulatory Elements (FAIRE)

FAIRE was performed as previously described with modifications^51^. Briefly, cells were crosslinked by adding formaldehyde to 1% final concentration directly to the cell culture medium and incubated 10 min at room temperature. Crosslinks were quenched by adding glycine to a final concentration of 0.125 M and incubated for 5 min at room temperature. Cells were washed 3 times with cold PBS and resuspended in 1 ml lysis buffer (1% SDS, 10 mM EDTA, 50 mM Tris HCl pH 8.1, 1 mM phenylmethylsulfonylfluoride, 10 mM NaF, 0.5 mM sodium orthovanadate, 10 µg/ml leupeptin, 10 µg/ml aprotinin). After 20 min of incubation on ice, samples were sonified using a COVARIS sonicator to reach an average DNA fragment size of ~300 bp. One aliquot of each sample was treated with 5 μl of proteinase K and incubated at 37 °C for 4 h, followed by an incubation at 65 °C for 6 h to reverse the crosslink, or left untreated. All samples were then extracted by phenol/chloroform and the free DNA contained in the upper phase was quantified by RT-qPCR. The fraction of free DNA is determined by dividing the amount of DNA obtained in the untreated sample by the amount obtained in the proteinase K-treated sample.

### Isolation of mouse primary naïve macrophages and T lymphocytes

*Cnot3* ^+/+^ or *Cnot3* ^fl/fl^ mice (Y. Imai and K. Kuba, unpublished) were crossed with mice expressing a fusion protein between Cre recombinase and the estrogen receptor (CreERT^2^) and injected with 1 mg tamoxifen on day 1, 2, 4 and 7 to induce the knock-out and analyzed at day 10^11^. Bone marrow cells were submitted for flow cytometry using antibodies against Mac-1 (BD Biosciences) to isolate macrophages. CD4^+^ T cells were purified by MACS from thymus via CD4^+^ beads (Miltenyi Biotec).

### Bioinformatic analysis and Statistics

Stored microarray data were evaluated using the R software and the Limma package^74^. Log mean spot signals were taken for further analysis. Background correction of data was done using the NormExp procedure on the negative control spots and quantile-normalized before averaging. Heat maps were produced using the pheatmap package. Proportional Venn diagrams were produced using the EulerAPE software^75^. Statistics (Mann-Whitney Rank, Fisher’s exact test, Wilcoxon signed rank or paired t-tests) were calculated using R, SigmaPlot11, GraphPadPrism 5.0 and MS EXCEL2010.

### Cell cycle analysis

The different cell cycle phases were measured by FACS after propidium iodide (PI) staining of the DNA content following the protocol described^76^. Cells were trypsinized, collected and washed with ice cold PBS. The cell pellet was resuspended in 500 μl of PBS and fixed by adding 4.5 ml of ice cold 70% ethanol. Cells were washed twice with PBS, and resuspended in PBS staining solution containing PI (20 μg/ml) and RNAse (0.2 mg/ml). Cells were incubated 30 min in the dark at room temperature, followed by analysis in a FACSCalibur device using the Cell Quest software from Becton Dickinson.

## Electronic supplementary material

Supplementary information

## References

[1] Haimovich G, Choder M, Singer RH, Trcek T (2013). The fate of the messenger is pre-determined: a new model for regulation of gene expression. Biochim Biophys Acta.

[2] Collart MA (2016). The Ccr4-Not complex is a key regulator of eukaryotic gene expression. Wiley Interdiscip Rev RNA.

[3] Ito K, Takahashi A, Morita M, Suzuki T, Yamamoto T (2011). The role of the CNOT1 subunit of the CCR4-NOT complex in mRNA deadenylation and cell viability. Protein Cell.

[4] Lau NC (2009). Human Ccr4-Not complexes contain variable deadenylase subunits. Biochem J.

[5] Maillet L, Tu C, Hong YK, Shuster EO, Collart MA (2000). The essential function of Not1 lies within the Ccr4-Not complex. J Mol Biol.

[6] Berthet C (2004). CCR4-associated factor CAF1 is an essential factor for spermatogenesis. Mol Cell Biol.

[7] Nakamura T (2004). Oligo-astheno-teratozoospermia in mice lacking Cnot7, a regulator of retinoid X receptor beta. Nat Genet.

[8] Morita M (2011). Obesity resistance and increased hepatic expression of catabolism-related mRNAs in Cnot3+/− mice. EMBO J.

[9] Watanabe C (2014). Stability of mRNA influences osteoporotic bone mass via CNOT3. Proc Natl Acad Sci USA.

[10] Neely GG (2010). A global *in vivo* Drosophila RNAi screen identifies NOT3 as a conserved regulator of heart function. Cell.

[11] Yang CY (2016). Interaction of CCR4-NOT with EBF1 regulates gene-specific transcription and mRNA stability in B lymphopoiesis. Genes Dev.

[12] Inoue T (2015). CNOT3 contributes to early B cell development by controlling Igh rearrangement and p53 mRNA stability. J Exp Med.

[13] Winkler GS, Mulder KW, Bardwell VJ, Kalkhoven E, Timmers HT (2006). Human Ccr4-Not complex is a ligand-dependent repressor of nuclear receptor-mediated transcription. EMBO J.

[14] Azzouz N, Panasenko OO, Colau G, Collart MA (2009). The CCR4-NOT complex physically and functionally interacts with TRAMP and the nuclear exosome. PLoS One.

[15] Kruk JA, Dutta A, Fu J, Gilmour DS, Reese JC (2011). The multifunctional Ccr4-Not complex directly promotes transcription elongation. Genes Dev.

[16] Kuzuoglu-Öztürk D (2016). miRISC and the CCR4-NOT complex silence mRNA targets independently of 43S ribosomal scanning. EMBO J.

[17] Peng W, Togawa C, Zhang K, Kurdistani SK (2008). Regulators of cellular levels of histone acetylation in Saccharomyces cerevisiae. Genetics.

[18] Mulder KW, Brenkman AB, Inagaki A, van den Broek NJ, Timmers HT (2007). Regulation of histone H3K4 tri-methylation and PAF complex recruitment by the Ccr4-Not complex. Nucleic acids research.

[19] Laribee RN (2007). CCR4/NOT complex associates with the proteasome and regulates histone methylation. Proc Natl Acad Sci USA.

[20] Haas M, Siegert M, Schurmann A, Sodeik B, Wolfes H (2004). c-Myb protein interacts with Rcd-1, a component of the CCR4 transcription mediator complex. Biochemistry.

[21] Deluen C (2002). The Ccr4-not complex and yTAF1 (yTaf(II)130p/yTaf(II)145p) show physical and functional interactions. Mol Cell Biol.

[22] Dutta A (2015). Ccr4-Not and TFIIS Function Cooperatively To Rescue Arrested RNA Polymerase II. Mol Cell Biol.

[23] Collart MA, Struhl K (1994). NOT1(CDC39), NOT2(CDC36), NOT3, and NOT4 encode a global-negative regulator of transcription that differentially affects TATA-element utilization. Genes Dev.

[24] Unanue ER, Turk V, Neefjes J (2016). Variations in MHC Class II Antigen Processing and Presentation in Health and Disease. Annu Rev Immunol.

[25] Malnati MS (1992). Processing pathways for presentation of cytosolic antigen to MHC class II-restricted T cells. Nature.

[26] Horton R (2004). Gene map of the extended human MHC. Nat Rev Genet.

[27] de Bakker PI (2006). A high-resolution HLA and SNP haplotype map for disease association studies in the extended human MHC. Nat Genet.

[28] Steimle V, Siegrist CA, Mottet A, Lisowska-Grospierre B, Mach B (1994). Regulation of MHC class II expression by interferon-gamma mediated by the transactivator gene CIITA. Science.

[29] Boss JM, Jensen PE (2003). Transcriptional regulation of the MHC class II antigen presentation pathway. Curr Opin Immunol.

[30] Ting JP, Trowsdale J (2002). Genetic control of MHC class II expression. Cell.

[31] Durand B (1997). RFXAP, a novel subunit of the RFX DNA binding complex is mutated in MHC class II deficiency. EMBO J.

[32] Masternak K (1998). A gene encoding a novel RFX-associated transactivator is mutated in the majority of MHC class II deficiency patients. Nat Genet.

[33] Steimle V (1995). A novel DNA-binding regulatory factor is mutated in primary MHC class II deficiency (bare lymphocyte syndrome). Genes Dev.

[34] Steimle V, Otten LA, Zufferey M, Mach B (1993). Complementation cloning of an MHC class II transactivator mutated in hereditary MHC class II deficiency (or bare lymphocyte syndrome). Cell.

[35] Handunnetthi L, Ramagopalan SV, Ebers GC, Knight JC (2010). Regulation of major histocompatibility complex class II gene expression, genetic variation and disease. Genes Immun.

[36] Mottok A (2015). Genomic Alterations in CIITA Are Frequent in Primary Mediastinal Large B Cell Lymphoma and Are Associated with Diminished MHC Class II Expression. Cell Rep.

[37] Quinn LL (2015). The Missing Link in Epstein-Barr Virus Immune Evasion: the BDLF3 Gene Induces Ubiquitination and Downregulation of Major Histocompatibility Complex Class I (MHC-I) and MHC-II. J Virol.

[38] Meerbrey KL (2011). The pINDUCER lentiviral toolkit for inducible RNA interference *in vitro* and *in vivo*. Proc Natl Acad Sci USA.

[39] Boland A (2013). Structure and assembly of the NOT module of the human CCR4-NOT complex. Nat Struct Mol Biol.

[40] Ito K (2011). CNOT2 depletion disrupts and inhibits the CCR4-NOT deadenylase complex and induces apoptotic cell death. Genes Cells.

[41] Mauxion F, Preve B, Seraphin B (2013). C2ORF29/CNOT11 and CNOT10 form a new module of the CCR4-NOT complex. RNA biology.

[42] Russell, P., Benson, J. D. & Denis, C. L. Characterization of mutations in NOT2 indicates that it plays an important role in maintaining the integrity of the CCR4-NOT complex. *J Mol Biol* **322**, 27–39, doi:S0022283602007076 [pii] (2002). 10.1016/s0022-2836(02)00707-6 12215412

[43] Temme C (2010). Subunits of the Drosophila CCR4-NOT complex and their roles in mRNA deadenylation. Rna.

[44] Winkler GS, Balacco DL (2013). Heterogeneity and complexity within the nuclease module of the Ccr4-Not complex. Front Genet.

[45] Han H (2015). TRRUST: a reference database of human transcriptional regulatory interactions. Sci Rep.

[46] Suzuki A, Niimi Y, Saga Y (2014). Interaction of NANOS2 and NANOS3 with different components of the CNOT complex may contribute to the functional differences in mouse male germ cells. Biol Open.

[47] Shi JX (2014). CNOT7/hCAF1 is involved in ICAM-1 and IL-8 regulation by tristetraprolin. Cell Signal.

[48] Kamon M (2014). Identification of Ccr4-not complex components as regulators of transition from partial to genuine induced pluripotent stem cells. Stem Cells Dev.

[49] Okada H, Schittenhelm RB, Straessle A, Hafen E (2015). Multi-functional regulation of 4E-BP gene expression by the Ccr4-Not complex. PLoS One.

[50] Dölken L (2008). High-resolution gene expression profiling for simultaneous kinetic parameter analysis of RNA synthesis and decay. Rna.

[51] Giresi PG, Kim J, McDaniell RM, Iyer VR, Lieb JD (2007). FAIRE (Formaldehyde-Assisted Isolation of Regulatory Elements) isolates active regulatory elements from human chromatin. Genome Res.

[52] Choi NM, Majumder P, Boss JM (2011). Regulation of major histocompatibility complex class II genes. Curr Opin Immunol.

[53] Zika E, Ting JP (2005). Epigenetic control of MHC-II: interplay between CIITA and histone-modifying enzymes. Curr Opin Immunol.

[54] van den Elsen PJ (2011). Expression regulation of major histocompatibility complex class I and class II encoding genes. Front Immunol.

[55] Wright KL, Ting JP (2006). Epigenetic regulation of MHC-II and CIITA genes. Trends Immunol.

[56] Vagaska B (2016). MHC-class-II are expressed in a subpopulation of human neural stem cells *in vitro* in an IFNgamma-independent fashion and during development. Sci Rep.

[57] Xie XW (2009). Expression of CIITA-related MHCII molecules in tumors linked to prognosis in hepatocellular carcinoma. Int J Oncol.

[58] Steidl C (2011). MHC class II transactivator CIITA is a recurrent gene fusion partner in lymphoid cancers. Nature.

[59] Cano F, Rapiteanu R, Sebastiaan Winkler G, Lehner PJ (2015). A non-proteolytic role for ubiquitin in deadenylation of MHC-I mRNA by the RNA-binding E3-ligase MEX-3C. Nat Commun.

[60] Rambout X (2016). The transcription factor ERG recruits CCR4-NOT to control mRNA decay and mitotic progression. Nat Struct Mol Biol.

[61] Nagarajan UM, Bushey A, Boss JM (2002). Modulation of gene expression by the MHC class II transactivator. J Immunol.

[62] Scharer CD (2015). Genome-wide CIITA-binding profile identifies sequence preferences that dictate function versus recruitment. Nucleic acids research.

[63] Wong D (2014). Genomic mapping of the MHC transactivator CIITA using an integrated ChIP-seq and genetical genomics approach. Genome biology.

[64] Villard J, Muhlethaler-Mottet A, Bontron S, Mach B, Reith W (1999). CIITA-induced occupation of MHC class II promoters is independent of the cooperative stabilization of the promoter-bound multi-protein complexes. Int Immunol.

[65] Zhou H, Su HS, Zhang X, Douhan J, Glimcher LH (1997). CIITA-dependent and -independent class II MHC expression revealed by a dominant negative mutant. J Immunol.

[66] Pai RK, Askew D, Boom WH, Harding CV (2002). Regulation of class II MHC expression in APCs: roles of types I, III, and IV class II transactivator. J Immunol.

[67] Bracken AP, Dietrich N, Pasini D, Hansen KH, Helin K (2006). Genome-wide mapping of Polycomb target genes unravels their roles in cell fate transitions. Genes Dev.

[68] Pasini D, Bracken AP, Jensen MR, Lazzerini Denchi E, Helin K (2004). Suz12 is essential for mouse development and for EZH2 histone methyltransferase activity. EMBO J.

[69] Rodriguez-Gil A (2016). HIPK family kinases bind and regulate the function of the CCR4-NOT complex. Mol Biol Cell.

[70] Morita M (2007). Depletion of mammalian CCR4b deadenylase triggers elevation of the p27Kip1 mRNA level and impairs cell growth. Mol Cell Biol.

[71] Takahashi A (2012). Involvement of CNOT3 in mitotic progression through inhibition of MAD1 expression. Biochem Biophys Res Commun.

[72] Saul VV, Niedenthal R, Pich A, Weber F, Schmitz ML (2015). SUMO modification of TBK1 at the adaptor-binding C-terminal coiled-coil domain contributes to its antiviral activity. Biochim Biophys Acta.

[73] Milanovic M, Kracht M, Schmitz ML (2014). The cytokine-induced conformational switch of nuclear factor kappaB p65 is mediated by p65 phosphorylation. Biochem J.

[74] Ritchie ME (2015). Limma powers differential expression analyses for RNA-sequencing and microarray studies. Nucleic acids research.

[75] Micallef L, Rodgers P (2014). eulerAPE: drawing area-proportional 3-Venn diagrams using ellipses. PLoS One.

[76] Riccardi C, Nicoletti I (2006). Analysis of apoptosis by propidium iodide staining and flow cytometry. Nat Protoc.

