# Supplementary material for: The CCR4-NOT complex contributes to repression of Major Histocompatibility Complex class II transcription
Source: Sci Rep. 2017 Jun 14;7:3547. doi: 10.1038/s41598-017-03708-7 (PMC5471237; doi:10.1038/s41598-017-03708-7)
Supplement: Supplementary file 1 — Supplementary information [file 41598_2017_3708_MOESM1_ESM.pdf]

Supplementary Material

The CCR4-NOT complex contributes to repression of Major Histocompatibility Complex class II transcription

Alfonso Rodríguez-Gil, Olesja Ritter, Vera V. Saul, Jochen Wilhelm, Chen-Yuan Yang, Rudolf Grosschedl, Yumiko Imai, Keiji Kuba, Michael Kacht, M. Lienhard Schmitz

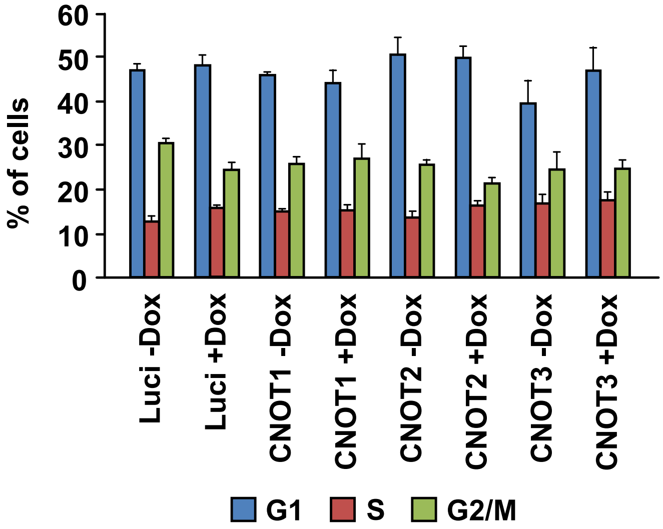

Supplementary Fig. 1. Effects of CNOT know-down on the cell cycle. Cells with a stably integrated pIND CNOT or a pIND Luci control plasmid were treated with Dox for 4 days or remained untreated. The cell cycle distribution was measured by FACS analysis after propidium iodide staining of the DNA. Error bars represent the standard deviation of three biological replicates.

Supplementary Table 1

pIND-CNOT1 AND 2 AND 3 +Dox / pIND-Luci +Dox, > 2 fold up	pIND-CNOT1 +Dox / pIND-Luci +Dox, > 2 fold up	pIND-CNOT2 +Dox / pIND-Luci +Dox, > 2 fold up	pIND-CNOT3 +Dox / pIND-Luci +Dox, > 2 fold up	pIND-CNOT1 AND 2 AND 3 +Dox / pIND-Luci +Dox, > 2 fold down	pIND-CNOT1 +Dox / pIND-Luci +Dox, > 2 fold down	pIND-CNOT2 +Dox / pIND-Luci +Dox, > 2 fold down	pIND-CNOT3 +Dox / pIND-Luci +Dox, > 2 fold down
ENST00000456813	ENST00000442269	ENST00000456813	ENST00000442269	Hs.577832	ENST00000372591	ENST00000435913	ENST00000372591
Hs.185688	ENST00000444663	ENST00000506335	ENST00000445031	Hs.662922	ENST00000413112	ENST00000507296	ENST00000423354
Hs.444913	ENST00000456813	Hs.185688	ENST00000456813	ABLIM2	ENST00000423354	ENST00000507992	ENST00000427317
Hs.446251	ENST00000540063	Hs.443430	Hs.111749	ACCSL	ENST00000425412	ENST00000540063	ENST00000435913
Hs.534931	Hs.111749	Hs.444913	Hs.185688	AGRN	ENST00000427317	Hs.344872	ENST00000442860
Hs.536338	Hs.178715	Hs.446251	Hs.444913	AMN1	ENST00000507992	Hs.536395	ENST00000506335
Hs.655804	Hs.185688	Hs.534931	Hs.446251	AMT	ENST00000512874	Hs.551860	ENST00000540063
Hs.677375	Hs.444913	Hs.536338	Hs.46423	ANKRD63	Hs.335413	Hs.567050	ENST00000546413
Hs.708976	Hs.446251	Hs.568467	Hs.467500	APBA2	Hs.373914	Hs.577832	Hs.335413
ABCC8	Hs.46423	Hs.655804	Hs.534931	ATP6V1C2	Hs.434403	Hs.625768	Hs.344872
ACAN	Hs.467500	Hs.677375	Hs.536338	BAAT	Hs.434604	Hs.646314	Hs.408455
ACOT1	Hs.474095	Hs.708976	Hs.640229	BPIFB1	Hs.453549	Hs.656848	Hs.434403
ACOT11	Hs.499594	Hs.712140	Hs.652196	BREA2	Hs.459590	Hs.662029	Hs.434604
ACVR2A	Hs.534931	Hs.730030	Hs.655804	C10orf128	Hs.472508	Hs.662922	Hs.498418
ACVRL1	Hs.536338	ABCA1	Hs.656851	C12orf74	Hs.560022	Hs.729655	Hs.534035
AP1AR	Hs.604039	ABCC8	Hs.659987	C17orf104	Hs.577832	ABLIM2	Hs.567050
APC2	Hs.631698	ACAN	Hs.677375	C20orf141	Hs.590653	ACCSL	Hs.577832
APH1B	Hs.640229	ACCS	Hs.708976	C2orf48	Hs.631805	ADD1	Hs.590653
AQP11	Hs.655804	ACOT1	Hs.712140	CCDC129	Hs.635171	AGAP11	Hs.631805
ARHGAP28	Hs.659987	ACOT11	Hs.720253	CCL27	Hs.637431	AGRN	Hs.635171
ARHGEF37	Hs.662412	ACTA2	Hs.720720	CD300A	Hs.655447	AHI1	Hs.662922
ARL6IP1	Hs.675446	ACVR2A	Hs.730762	CDH26	Hs.661155	AMN1	Hs.667258
ARPM1	Hs.677375	ACVRL1	ABCC6	CDKN2B-AS	Hs.662922	AMT	Hs.715044
ATL1	Hs.708976	AFAP1L2	ABCC8	CEP97	Hs.667258	ANKH	Hs.729467
ATP1A3	Hs.730762	ALOX5	ABHD3	CTSZ	Hs.669000	ANKRD13C	Hs.730425
B4GALNT1	ABCB6	ANK1	ACADSB	DCN	Hs.729231	ANKRD19P	Hs.731138
BAIAP2L2	ABCB8	ANKRD37	ACAN	DIO2	Hs.729596	ANKRD52	AASS
BATF2	ABCC4	ANXA1	ACAT2	DIRC1	Hs.731138	ANKRD63	ABCA1
BEX5	ABCC8	AP1AR	ACOT1	DKFZp686M1136	A1BG	APBA2	ABCA3
C14orf128	ABCD4	APC2	ACOT11	EML6	A4GALT	ARHGAP26	ABCD1
C14orf142	ABHD11	APH1B	ACTA1	ERBB4	ABCA3	ARHGEF1	ABCG1
C14orf149	ABHD3	APOC1	ACTR5	FADS3	ABCB1	ATAD5	ABHD1
C1orf182	ABHD4	APOE	ACVR2A	GADD45G	ABCD1	ATP6V1C2	ABHD8
C1R	ACADM	AQP11	ACVRL1	GDNF	ABCG1	ATXN7L1	ABL1
C21orf67	ACADSB	ARHGAP27	ACYP2	GNRHR2	ABCG4	BAAT	ABLIM2
C21orf90	ACAN	ARHGAP28	ADAM1	GPR1	ABHD8	BACE2	ACAD10
C3orf67	ACOT1	ARHGEF37	ADC	GSDMB	ABI2	BPIFB1	ACCN2
C6orf174	ACOT11	ARL6IP1	ADI1	H19	ABLIM2	BREA2	ACCSL
C6orf225	ACRC	ARPM1	AFF3	HTR2A	ABLIM3	C10orf128	ACOX1
CASD1	ACTA1	ASPHD1	AGA	IFI27	ACAD10	C10orf2	ACOX3
CC2D2A	ACTA2	ATF6B	AGFG2	IL10RA	ACBD3	C12orf74	ACVR2B
CCDC113	ACTR5	ATL1	AGPAT4	KAZN	ACCN2	C16orf79	ADAM11
CCDC96	ACTR8	ATP1A3	AIMP2	KCNC4	ACCN4	C17orf104	ADAMTS13
CD19	ACVR2A	ATP6V1G2	AK2	KIR3DL1	ACCSL	C18orf1	ADAMTS3
CHGA	ACVRL1	B3GALT4	AK8	KRTAP19-2	ACOT13	C18orf19	ADAMTS9
CMTM1	ACYP2	B4GALNT1	AKR7A2P1	LOC100128977	ACOXL	C1orf38	ADAP2
CNRIP1	ADAM1	BAIAP2L2	ALDOC	LOC100131107	ACP1	C1S	ADCY1
CPNE5	ADCK2	BATF2	ALKBH8	LOC100132832	ACVR1C	C20orf141	ADM
CREBL2	AGA	BEGAIN	ALX1	LOC100169752	ACVR2B	C2orf48	ADM2
CRIP3	AGFG2	BEX5	ALX4	LOC100287506	ACYP1	C2orf63	ADRA1B
CTSO	AGMAT	BIK	AMDHD2	LOC100288420	ADAM11	C2orf68	ADRB1
CUX2	AHSA1	BRPF3	ANKMY1	LOC100289137	ADAM15	C6orf124	ADSSL1
DENND2A	AIMP2	BST2	ANKRD34A	LOC100505478	ADAM17	C6orf162	AES
DISP2	AK2	BTG2	ANKRD37	LOC100505876	ADAMTS1	C7orf43	AGAP7
DLGAP3	AK8	C11orf52	ANP32D	LOC100506586	ADAMTS9	CAPN7	AGER
DLK2	ALDH5A1	C11orf63	AP1AR	LOC100507353	ADM2	CAPS	AGMAT
DNAJC30	ALDH6A1	C11orf70	APBA3	LOC100507363	ADPRHL2	CCDC129	AGPAT2
DNM1P46	ALKBH1	C14orf102	APC2	LOC100652741	ADRB1	CCL27	AGPAT5
DQX1	ALKBH8	C14orf128	APH1B	LOC100652769	AGAP7	CCNF	AGRN
DYRK3	AMDHD2	C14orf142	APLP1	LOC100653323	AGRN	CCNJL	AGSK1
ERAP2	AMMECR1	C14orf149	APOE	LOC202781	AGSK1	CD300A	AGXT2L2
ESPNL	ANGEL1	C14orf43	APOL1	LOC221442	AHSA2	CD86	AIF1L
EXOC6B	ANKMY1	C14orf79	APOL2	LOC256880	AIM1L	CDH26	AKIRIN2-AS1
FAM118B	ANKRD34A	C16orf11	AQP11	LOC340335	AKT3	CDKN2B-AS	AKT3
FAM122C	ANP32D	C16orf45	ARFGAP2	LOC348120	AKTIP	CENPT	ALDH1A3
FAM155A	ANTXR1	C17orf100	ARHGAP28	LOC390940	ALDH1A3	CEP97	ALOX5
FAM174B	AP1AR	C18orf56	ARHGEF37	LOC400657	ALOX5	CHIC1	AMN1
FARS2	APBB3	C1orf106	ARL6IP1	LOC402160	AMN1	CISH	AMT
FAT4	APC2	C1orf182	ARMC9	LOC439950	AMOT	CNOT2	ANK1
FLVCR1	APEX1	C1R	ARMCX5	LOC642924	AMT	CRCP	ANKH
GABBR2	APH1B	C21orf67	ARNT2	LOC727982	ANK1	CTSZ	ANKRD1
GAL3ST1	APLP1	C21orf90	ARNTL	LOC728802	ANKRA2	DCN	ANKRD10
GALC	APOLD1	C3orf67	ARPM1	MUCL1	ANKRD1	DDX46	ANKRD12
GBAP1	AQP11	C5orf35	ASGR1	NEIL2	ANKRD23	DDX52	ANKRD19P
GCC1	ARFGAP2	C5orf56	ASL	NHSL2	ANKRD33B	DEM1	ANKRD20A5P
GEMIN2	ARFGEF2	C6orf126	ASPHD2	OR5D14	ANKRD39	DIO2	ANKRD33B
GLT8D2	ARHGAP28	C6orf154	ASPRV1	PAPLN	ANKRD57	DIRC1	ANKRD57
GPER	ARHGEF37	C6orf170	ASTE1	PDLIM4	ANKRD63	DKFZp686M1136	ANKRD63
HDX	ARID1B	C6orf174	ASXL3	PDXDC2P	ANO5	DNMT3A	ANKRD9
HIST1H3J	ARID5B	C6orf225	ATG5	PIK3CD	ANXA3	EEPD1	ANKS6
HLA-DMA	ARL6IP1	C9orf103	ATL1	PLA2G4E	AP1S3	EML6	ANXA3
HLA-DOA	ARNT2	C9orf171	ATP1A3	PON3	APBA2	ENTPD7	AP1S1
HLA-DRB3	ARPM1	CAMK2N2	ATP6V1G2	POU4F3	APOBEC3C	ERBB4	AP3M2
HLA-DRB5	ASL	CAPG	AVEN	Q5SRJ3	AR	ERC2	APBA1
HOXC12	ASPRV1	CASD1	B3GALT4	RASSF3	ARC	FADS3	APBA2
IFI35	ASRGL1	CC2D2A	B4GALNT1	RGS12	ARFGAP3	FAM198A	APOBEC3B
IL17D	ASTE1	CCDC103	BAG1	RN5-8S1	ARHGAP11A	FAM81A	ARC
IL20RB	ATF7IP	CCDC113	BAG2	RNF223	ARHGAP19	FARP1	ARHGAP11A
INA	ATG13	CCDC153	BAIAP2L2	SDHAP1	ARHGAP8	FBXL2	ARHGAP19
ITGA1	ATG2A	CCDC88C	BATF2	SEC14L2	ARHGEF10L	FCRL5	ARHGAP5
ITGB4	ATG5	CCDC96	BBS1	SH2D6	ARHGEF16	FLJ33996	ARHGEF1
IZUMO1	ATL1	CCR10	BCCIP	SIGLEC15	ARID3B	FLNB	ARHGEF16
KCNA6	ATP1A3	CD19	BCKDHB	SLC46A2	ARPC1B	FLNC	ARHGEF9
KCNC3	ATP5S	CD24	BEND5	SLC5A9	ARPC2	FOXD4	ARNTL2
KCNN2	ATP6V0D1	CECR6	BET1	SMTNL2	ARRDC4	GADD45G	ARPC4-TTLL3
KCNRG	ATP6V1D	CENPBD1	BEX2	SNORA17	ASAH1	GCNT1	ARRDC1
KCTD18	ATXN7L3B	CGA	BEX5	SNORD12B	ASS1	GDNF	ASB6
KIAA0408	AVEN	CHGA	BFSP1	SP140	ATAD3C	GNAQ	ASS1
KRT222	B3GNT8	CMTM1	BMP2	SP8	ATF3	GNRHR2	ATF3
LBX2	B4GALNT1	CNRIP1	BRCC3	SREBF2	ATG12	GPATCH1	ATP6V1C2
LINGO3	B4GALT7	CPNE5	BRD8	SYT15	ATHL1	GPR1	ATP8A1
LOC100132147	BACE1	CPPED1	BRPF3	TBC1D5	ATL3	GPR35	ATP8B3
LOC100132356	BAG1	CREBL2	BST2	TCF24	ATM	GPR88	ATRN
LOC100233156	BAG5	CRIP2	C10orf114	TM6SF1	ATP10B	GSDMB	ATXN1
LOC100286925	BAI1	CRIP3	C10orf140	USP32	ATP1B2	GUCA1B	B3GALNT1
LOC100506302	BAIAP2L2	CRYGD	C11orf70	USP49	ATP6V1C2	GXYLT1	B3GNT4
LOC100507047	BATF2	CSPG4	C12orf26	VN1R2	ATP8A1	H19	B4GALT1
LOC100507437	BAZ1A	CTSO	C12orf4	XLOC_000182	ATP8B3	HAS3	BAAT
LOC100510710	BBS1	CUX2	C12orf45	XLOC_000670	ATXN1	HIST1H2AL	BARX1
LOC153546	BCKDHB	CYP26A1	C12orf53	XLOC_000776	B3GALNT1	HIST1H4A	BASP1
LOC170425	BCLAF1	DACT3	C14orf128	XLOC_001357	B4GALNT4	HIST1H4F	BBIP1
LOC282997	BEGAIN	DDX31	C14orf142	XLOC_001373	B4GALT1	HIST1H4H	BCL2L11
LOC401068	BEX2	DENND2A	C14orf149	XLOC_001453	BAAT	HIST1H4I	BHLHE40
LOC642366	BEX5	DES	C14orf159	XLOC_001515	BAHCC1	HIST1H4J	BHLHE41
LOC644145	BFSP1	DHX58	C14orf79	XLOC_001856	BARX1	HIST1H4K	BIK
LOC646999	BHLHB9	DIRC2	C15orf17	XLOC_002063	BASP1	HIST2H4B	BMF
LOC648987	BLCAP	DISP2	C16orf45	XLOC_002283	BAZ2A	HKR1	BMP4
LOC92659	BRCC3	DLGAP3	C17orf72	XLOC_002581	BBIP1	HNRNPA0	BMS1

Supplementary Table 1

pIND-CNOT1 AND 2 AND 3 +Dox / pIND-Luci +Dox, > 2 fold up	pIND-CNOT1 +Dox / pIND-Luci +Dox, > 2 fold up	pIND-CNOT2 +Dox / pIND-Luci +Dox, > 2 fold up	pIND-CNOT3 +Dox / pIND-Luci +Dox, > 2 fold up	pIND-CNOT1 AND 2 AND 3 +Dox / pIND-Luci +Dox, > 2 fold down	pIND-CNOT1 +Dox / pIND-Luci +Dox, > 2 fold down	pIND-CNOT2 +Dox / pIND-Luci +Dox, > 2 fold down	pIND-CNOT3 +Dox / pIND-Luci +Dox, > 2 fold down
LPIN2	BRF1	DLGAP5	C17orf85	XLOC_002900	BCL3	HOXA4	BOD1L
LRIF1	BRMS1	DLK2	C18orf21	XLOC_003165	BCL6	HTR2A	BPIFB1
LRRC56	BRMS1L	DNAJC30	C19orf51	XLOC_004452	BCR	IDUA	BRD3
MAPK8IP2	BTBD6	DNM1P46	C1orf182	XLOC_004680	BEND4	IFI27	BREA2
MARVELD2	BTBD7	DOC2A	C1orf53	XLOC_005051	BHLHE40	IL10RA	BSPRY
MBLAC2	C10orf114	DOK7	C1orf54	XLOC_005633	BHLHE41	IL17RC	BTG2
MDGA2	C10orf140	DQX1	C1orf55	XLOC_006419	BIK	IST1	C10orf128
METTL1	C11orf57	DYRK3	C1orf74	XLOC_006844	BMP2	ITPKB	C10orf82
MGC4473	C11orf84	EDNRA	C1R	XLOC_007855	BMS1	JRK	C11orf30
MTERF	C11orf95	EGFLAM	C20orf29	XLOC_008005	BPIFB1	JUNB	C11orf54
NEURL2	C12orf26	ELFN2	C21orf2	XLOC_008586	BREA2	KAZN	C11orf96
NHEJ1	C12orf32	ELL	C21orf56	XLOC_008652	BROX	KCNC4	C12orf57
NOS2	C12orf34	ENO1-AS1	C21orf67	XLOC_009451	BRWD3	KGFLP2	C12orf74
NOS3	C14orf101	EPCAM	C21orf90	XLOC_009628	BSPRY	KHK	C14orf33
NOVA2	C14orf109	ERAP2	C21orf91	XLOC_010167	BTN2A3P	KIAA0564	C15orf59
NR0B1	C14orf118	ESPNL	C22orf46	XLOC_011306	C10orf128	KIAA1841	C16orf61
NTRK2	C14orf126	EXOC6B	C2orf49	XLOC_011407	C10orf47	KIR3DL1	C16orf79
NUDT17	C14orf128	EYA1	C2orf74	XLOC_011984	C11orf30	KIRREL2	C17orf104
NUDT4	C14orf129	F8A2	C2orf81	XLOC_012338	C11orf54	KLHDC9	C17orf28
NYNRIN	C14orf133	FAM118B	C3orf14	XLOC_012678	C11orf96	KRTAP19-2	C18orf1
OLIG1	C14orf135	FAM122C	C3orf67	XLOC_013162	C12orf53	KRTAP9-6	C18orf19
PARP10	C14orf142	FAM150B	C5orf35	XLOC_013434	C12orf57	LAT	C18orf45
PAX1	C14orf149	FAM155A	C5orf56	XLOC_013449	C12orf74	LCAT	C19orf50
PCDHGA8	C14orf159	FAM174B	C6orf154	XLOC_013679	C15orf61	LIME1	C1orf133
PDE3B	C14orf167	FAM176A	C6orf174	XLOC_I2_000339	C16orf61	LINC00340	C1orf151-NBL1
PIP4K2C	C14orf21	FAM78A	C6orf225	XLOC_I2_003039	C17orf101	LOC100127891	C1orf201
PLEKHA4	C14orf43	FAM90A7	C6orf226	XLOC_I2_004168	C17orf104	LOC100128977	C1orf213
PLTP	C14orf45	FARS2	C7orf29	XLOC_I2_005997	C17orf28	LOC100129034	C1orf9
PNMT	C17orf59	FAT4	C7orf36	XLOC_I2_007585	C17orf48	LOC100130027	C1QL1
PPAP2B	C17orf72	FBLN5	C7orf74	XLOC_I2_011145	C17orf58	LOC100131000	C20orf141
PPEF1	C17orf85	FBXO2	C9orf100	XRRA1	C19orf55	LOC100131107	C20orf160
PRKCB	C19orf23	FCRLB	C9orf171	YPEL1	C1orf109	LOC100132249	C22orf43
PRSS35	C19orf51	FDXR	CA14	ZDHHC23	C1orf114	LOC100132832	C2CD2L
PSMB9	C1orf182	FLT3LG	CA2	ZNF141	C1orf133	LOC100169752	C2orf48
PTBP1	C1orf53	FLVCR1	CALML3	ZNF157	C1orf151-NBL1	LOC100287506	C2orf69
PTGDS	C1orf55	FLVCR1-AS1	CASD1	ZRANB1	C1orf159	LOC100287616	C3orf52
RAB39B	C1orf74	FNDC1	CBR1		C1orf201	LOC100288420	C5orf41
RAB3C	C1R	FOLR1	CC2D2A		C1orf213	LOC100289137	C6orf228
RANBP6	C20orf29	FOXN4	CCDC103		C1orf63	LOC100292905	C6orf26
RBFOX3	C21orf67	FUT8	CCDC113		C1orf86	LOC100293516	C6orf57
RCBTB2	C21orf90	FZD10	CCDC134		C1QL1	LOC100499489	C7orf10
RELL2	C22orf46	GABBR2	CCDC138		C1QTNF6	LOC100505478	C7orf43
RIIAD1	C3orf67	GAD1	CCDC150		C20orf141	LOC100505687	C7orf46
RTN1	C4orf42	GAL3ST1	CCDC153		C2CD4C	LOC100505876	C8orf40
SCARA3	C5orf34	GALC	CCDC58		C2orf48	LOC100506528	C8orf44
SCN4B	C6orf174	GALNT14	CCDC96		C3orf52	LOC100506586	C8orf47
SLC22A17	C6orf203	GBAP1	CCS		C4orf52	LOC100506661	C8orf58
SLC27A6	C6orf225	GCC1	CD19		C5orf32	LOC100506778	C9orf140
SLC37A1	C6orf226	GDF15	CDC73		C5orf41	LOC100507353	C9orf142
SNAI2	C7orf29	GEM	CDK5		C6orf124	LOC100507363	C9orf16
SPATA20	C7orf36	GEMIN2	CDS2		C6orf136	LOC100507473	C9orf3
SPEF1	C7orf49	GLT8D2	CEP112		C6orf26	LOC100507645	C9orf30
SPIN3	C7orf74	GNG4	CEP19		C6orf57	LOC100508995	C9orf5
SPINK5	C8orf33	GORAB	CFH		C7orf10	LOC100652738	C9orf69
SPTBN1	C8orf55	GPER	CHGA		C7orf46	LOC100652741	C9orf7
SSBP3	C9orf100	GSC	CHORDC1		C8orf44	LOC100652758	C9orf86
STARD8	C9orf103	GSTO2	CHRNA3		C8orf47	LOC100652769	CA8
STEAP1	C9orf116	GUCY1B2	CHST4		C8orf58	LOC100653030	CACNA1H
STEAP1B	C9orf125	HAUS4	CIRBP		C9orf46	LOC100653323	CACNA2D3
STXBP6	C9orf156	HDGFL1	CLCN7		C9orf72	LOC147804	CACNG6
TAP1	C9orf69	HDX	CLUAP1		CAB39L	LOC202781	CALCOCO1
TBC1D8B	C9orf91	HEY2	CMTM1		CACNA1H	LOC221442	CALHM2
THAP7-AS1	CA2	HIST1H2BK	CNRIP1		CACNA2D3	LOC256880	CALML4
TLCD1	CABLES2	HIST1H3J	COLEC11		CACNB3	LOC257152	CAPN7
TMEM173	CALM1	HLA-DMA	COLEC12		CACNB4	LOC283624	CAPS
TMEM22	CAMK2N2	HLA-DMB	COMMD2		CACNG6	LOC284408	CARHSP1
TNFAIP2	CASD1	HLA-DOA	COMMD9		CALCA	LOC285095	CASZ1
TNNI3	CBX5	HLA-DPA1	CORO1B		CALHM2	LOC286109	CCBL1
TNS1	CC2D2A	HLA-DPB1	CPNE5		CALML4	LOC340335	CCDC129
TOB2P1	CCDC113	HLA-DRB1	CREB3		CAMK1D	LOC348120	CCDC3
TP53I3	CCDC144A	HLA-DRB3	CREBL2		CAMK2G	LOC386758	CCDC6
TRPV2	CCDC43	HLA-DRB5	CRIP1		CAMK2N1	LOC390940	CCDC88C
TSPAN10	CCDC96	HMOX1	CRIP3		CAMTA1	LOC400657	CCL27
TSPAN12	CCND1	HOPX	CRYGD		CAP1	LOC400958	CCM2
TTC8	CD19	HOXC12	CRYM		CAPZB	LOC402160	CCNB1IP1
UBR5	CDC42SE2	HRASLS5	CRYZ		CASP9	LOC439950	CCNF
UFSP1	CDCA4	IFI35	CSPG4		CASZ1	LOC441666	CCNJ
UNC5C	CDH22	IL17D	CTPS2		CBFA2T2	LOC442028	CCNJL
VAV3	CDK19	IL20RB	CTSO		CBX7	LOC642924	CCNY
VAX1	CDO1	IL27RA	CUL4A		CCBL1	LOC644339	CD109
VAX2	CDS2	IL4I1	CUX2		CCDC107	LOC647946	CD24
VSIG10L	CECR6	INA	CWC25		CCDC109B	LOC727982	CD300A
WBSCR27	CENPB	INPP5D	CYB561		CCDC121	LOC728802	CD68
WIBG	CEP112	IRF8	CYB5B		CCDC129	LOC729444	CDAN1
WNT10B	CEP164	IRX4	CYB5D1		CCDC151	LOC730102	CDC14B
XLOC_000167	CHAMP1	ISG20	CYB5RL		CCL16	MAF1	CDC14C
XLOC_000175	CHGA	ITGA1	CYP2J2		CCL27	MBD5	CDC42
XLOC_000190	CHMP1B	ITGB4	DACH1		CCR10	MCM9	CDH1
XLOC_000822	CHMP4A	IZUMO1	DACT3		CD109	MDFIC	CDH23
XLOC_000883	CHMP6	KANK3	DBNL		CD300A	MDN1	CDH26
XLOC_002026	CHORDC1	KCNA6	DBP		CD302	MFSD10	CDH3
XLOC_005764	CHRAC1	KCNC3	DDRGK1		CD55	MFSD9	CDK10
XLOC_007348	CHST4	KCNG3	DDTL		CD68	MGC23284	CDK5RAP2
XLOC_007433	CHURC1	KCNIP3	DDX19B		CD70	MIR22HG	CDKN1A
XLOC_007769	CINP	KCNN2	DDX27		CD86	MOCS3	CDKN2B-AS
XLOC_009147	CKMT1A	KCNRG	DENND2A		CDAN1	MRGPRG	CDKN2D
XLOC_009509	CLCN7	KCTD18	DES		CDC42	MRPS25	CECR5-AS1
XLOC_011344	CLGN	KIAA0408	DGAT2		CDH26	MTRNR2L7	CELF2
XLOC_013301	CLN6	KIAA1257	DHCR24		CDK10	MUCL1	CELSR3
XLOC_014063	CLTB	KIF25	DHDH		CDK18	MYCNOS	CENPJ
XLOC_014397	CLUAP1	KIF3C	DHRS4		CDKN1A	MYPOP	CEP72
XLOC_I2_011649	CMTM1	KLC3	DHRS4L2		CDKN2B-AS	NACC1	CEP97
XLOC_I2_013267	CNRIP1	KLF2	DHX29		CDKN2D	NBPF1	CFL2
ZBTB25	COL5A1	KLK8	DHX34		CECR5-AS1	NCAPG	CGA
ZCWPW1	COMMD9	KLRG2	DHX37		CELF2	NEIL2	CGGBP1
ZNF251	COQ4	KRT222	DHX58		CELSR3	NFYA	CHIT1
ZNF280D	CPNE5	LAMP3	DHX9		CENPJ	NHSL2	CHMP4C
ZNF498	CREBBP	LBX2	DIDO1		CEP120	NISCH	CHRD
ZNF667	CREBL2	LDHD	DIEXF		CEP44	NSUN6	CHRM3
ZSWIM5	CRIP3	LENG9	DIS3L		CEP97	NT5DC2	CHST15
	CTAGE11P	LEPREL1	DISC1		CFLAR	NT5DC3	CHST8
	CTAGE5	LGALS1	DISP2		CHMP5	NUCB1	CLDND1
	CTPS2	LGALS3BP	DKK1		CHRD	ONECUT3	CLK3
	CTSL1P2	LIMS2	DKKL1		CHRM3	OR5D14	CLPB
	CTSL1P8	LINC00482	DLD		CIB2	P2RY1	CLU
	CTSO	LINGO1	DLEU2L		CITED4	PAPLN	CMPK1
	CUL4A	LINGO3	DLGAP3		CLASRP	PCSK6	CNOT3
	CUX2	LIPT2	DLK2		CLIC4	PDLIM4	CNPY3
	CWC25	LMO1	DLX5		CLIP2	PDXDC2P	CNTRL

Supplementary Table 1

pIND-CNOT1 AND 2 AND 3 +Dox / pIND-Luci +Dox, > 2 fold up	pIND-CNOT1 +Dox / pIND-Luci +Dox, > 2 fold up	pIND-CNOT2 +Dox / pIND-Luci +Dox, > 2 fold up	pIND-CNOT3 +Dox / pIND-Luci +Dox, > 2 fold up	pIND-CNOT1 AND 2 AND 3 +Dox / pIND-Luci +Dox, > 2 fold down	pIND-CNOT1 +Dox / pIND-Luci +Dox, > 2 fold down	pIND-CNOT2 +Dox / pIND-Luci +Dox, > 2 fold down	pIND-CNOT3 +Dox / pIND-Luci +Dox, > 2 fold down
	CXXC1	LOC100128737	DMD		CLSTN3	PFAS	COBL
	CYB561D1	LOC100132147	DNAJA1		CLU	PIK3CD	COBRA1
	CYB5B	LOC100132163	DNAJA1P5		CLYBL	PIP5K1C	COL12A1
	CYB5D1	LOC100132356	DNAJB4		CNOT1	PLA2G4E	COL13A1
	CYB5RL	LOC100144602	DNAJC30		CNPPD1	PON3	COL1A1
	CYBASC3	LOC100233156	DNAJC8		CNPY1	POU4F3	COL25A1
	CYP26A1	LOC100286925	DNM1P46		COA5	PPFIA3	COL2A1
	CYP2J2	LOC100287177	DNTTIP1		COBL	PPP1R3F	COL5A2
	DBNL	LOC100287482	DOCK4		COL25A1	PRRC2B	CORO2A
	DCAF11	LOC100506125	DPAGT1		COL5A2	Q5SRJ3	CR2
	DCAF4	LOC100506302	DPY19L1		COQ10B	Q7Z2X8	CRAT
	DDTL	LOC100507047	DPYSL4		CORO2A	QKI	CREM
	DDX19A	LOC100507437	DQX1		COTL1	RASSF3	CRLF1
	DDX19B	LOC100507458	DTNA		CPAMD8	RDH13	CRYAB
	DDX24	LOC100510710	DTWD1		CPEB1	RGS12	CRYBG3
	DDX28	LOC100630918	DYRK3		CPNE7	RHBDL1	CRYL1
	DEM1	LOC100653206	EAPP		CPS1	RN18S1	CSGALNACT2
	DENND2A	LOC143666	EEPD1		CR2	RN5-8S1	CSRNP1
	DEPTOR	LOC153546	EFCAB2		CRABP2	RNF222	CT45A1
	DGAT2	LOC170425	EFEMP1		CRADD	RNF223	CT45A5
	DGCR5	LOC255512	EGFLAM		CREB5	RNF41	CTDP1
	DHCR24	LOC282997	EHD2		CRHR1	RNU6ATAC	CTIF
	DHFR	LOC283663	EIF2B4		CRLF1	RPL13P5	CTNNAL1
	DHRS4	LOC388780	ELMO2		CRLF3	RPL36A-HNRNPH2	CTSB
	DHRS4L1	LOC400099	ELOVL2		CROCC	SCAND2	CTSC
	DHRS4L2	LOC401068	EMG1		CROCCP2	SCAND3	CTSZ
	DHRS7	LOC642366	ENOSF1		CRYAB	SCARNA7	CXXC5
	DHX29	LOC644145	EPHX2		CRYL1	SDHAP1	CYBA
	DHX37	LOC645638	ERAP2		CSGALNACT2	SEC14L2	CYFIP2
	DHX40	LOC646999	ERCC4		CSRNP1	SETD5	CYP27B1
	DHX9	LOC648987	ESPNL		CSRNP3	SH2D6	CYP2R1
	DIDO1	LOC730081	EXOC6B		CT45A1	SIGLEC15	DAAM2
	DIEXF	LOC92659	EXOSC3		CT45A5	SKIL	DCAKD
	DIRC2	LOXL4	EYA1		CTBS	SLC46A2	DCN
	DIS3L	LPIN2	FABP5		CTGF	SLC4A2	DDAH1
	DISP2	LRIF1	FAM114A2		CTSB	SLC4A7	DDB2
	DKFZP564C152	LRRC38	FAM117A		CTSC	SLC5A9	DDHD2
	DLD	LRRC56	FAM118B		CTSK	SLC7A5	DDX17
	DLGAP3	LRRC57	FAM122C		CTSZ	SLMAP	DDX51
	DLGAP5	LY6K	FAM123B		CTXN1	SMAD2	DEAF1
	DLK2	MAPK8IP2	FAM126A		CUL7	SMTNL2	DECR1
	DMKN	MARVELD2	FAM155A		CUL9	SNORA11E	DEFB110
	DNAAF2	MBLAC2	FAM174B		CXorf38	SNORA17	DENND1A
	DNAJB4	MDGA2	FAM176A		CYB5A	SNORA23	DFNB31
	DNAJC30	MESP2	FAM188A		CYBRD1	SNORA26	DHPS
	DNAJC8	METTL1	FAM203A		CYR61	SNORA56	DHX32
	DNM1P46	METTL4	FAM207A		DALRD3	SNORA71C	DICER1-AS
	DOC2A	MGC12488	FAM5C		DBF4B	SNORA74B	DIO2
	DOLK	MGC4473	FAM71E1		DBNDD2	SNORA80B	DIP2C
	DPYSL4	MICB	FAM90A7		DCAF10	SNORA84	DIRC1
	DQX1	MSX2P1	FARS2		DCAF8	SNORD105B	DKFZp686M1136
	DUSP16	MT1F	FAT4		DCLK1	SNORD12B	DLC1
	DYRK2	MTERF	FBXL12		DCN	SNORD17	DLG4
	DYRK3	MVP	FCRLB		DDAH2	SNORD18B	DLL1
	E2F1	MYO5C	FGF13		DDHD2	SNORD33	DLX4
	EAPP	MYOD1	FGFBP3		DDOST	SNORD4B	DMC1
	EEPD1	MYOF	FGFR1OP		DDX17	SNORD52	DNAJC14
	EGR1	NAGS	FKBP7		DDX26B	SNORD87	DNASE2
	EID2B	NEURL2	FLJ30901		DECR1	SOCS7	DOCK1
	EIF2B2	NHEJ1	FLJ35776		DEFB110	SOS1	DOK7
	EIF2S1	NHLH2	FLJ45340		DENND4C	SP140	DOLPP1
	EIF4G2	NKX2-4	FLRT3		DFNA5	SP8	DOT1L
	EIF6	NMU	FLT3LG		DFNB31	SPATA2	DPP4
	ELAC1	NOS2	FLVCR1		DHODH	SPINK7	DPY19L1P1
	ELMO2	NOS3	FLVCR1-AS1		DHPS	SPRR2D	DRD4
	ELOVL2	NOVA2	FNDC4		DHRS3	SREBF2	DSC3
	EMG1	NPAT	FNIP1		DIO2	SYT15	DUSP1
	EPB41L4B	NPY	FOXG1		DIP2A	TADA2B	DUSP22
	EPDR1	NR0B1	FOXO4		DIRC1	TATDN2	DUSP4
	EPHX2	NTRK2	FOXRED1		DIS3L2	TBC1D17	DUSP5
	ERAP2	NUDT17	FPGT		DKFZp686M1136	TBC1D3	DUSP8
	ERH	NUDT18	FUBP1		DLC1	TBC1D5	DVL1
	ESPNL	NUDT4	FUS		DLG4	TCEAL6	E2F2
	EXOC6B	NYNRIN	FZD8		DLL1	TCEANC	E2F8
	F8A1	ODF3B	GABBR2		DLX4	TCF24	ECE1
	F8A2	OLFML2A	GAL3ST1		DMC1	TM6SF1	EDA2R
	FABP5	OLIG1	GALC		DNAH14	TMEM115	EEF1A2
	FAM101B	OXCT1	GALNT1		DNAJB5	TMEM74	EEF2
	FAM118B	PACSIN1	GAMT		DNAJC24	TMEM95	EFHD1
	FAM120AOS	PAQR9	GATA4		DNAJC27	TNFAIP3	EGFL7
	FAM120C	PARP10	GBA		DNASE2	TONSL	EHMT1
	FAM122C	PAX1	GBAP1		DNM3	TRMT61B	EIF4G3
	FAM123B	PAX7	GCAT		DNMT3A	TXNDC17	ELF4
	FAM134B	PCDHGA8	GCC1		DOCK8	TXNRD1	ELK3
	FAM13A	PCK1	GEMIN2		DOK7	TYW1B	ELK4
	FAM155A	PCP2	GFER		DOT1L	UBA6	ELOVL1
	FAM155B	PDE3B	GIT2		DPM3	UBXN2B	ELOVL7
	FAM156B	PEX11A	GLOD4		DPP4	USP32	EMILIN2
	FAM158A	PHYHD1	GLRX2		DPY19L1P1	USP49	EMILIN3
	FAM174B	PIGB	GLT8D2		DUSP1	VN1R2	EML6
	FAM188A	PIGZ	GMFG		DUSP4	WHAMMP2	EMX1
	FAM192A	PIP4K2C	GMPPA		DUSP8	WHSC1	ENTPD4
	FAM203A	PLEKHA4	GNAS-AS1		DUSP9	XLOC_000152	EP400NL
	FAM91A1	PLTP	GNG11		DVL1	XLOC_000182	EPAS1
	FANCC	PMEL	GNPTG		DYNC1I2	XLOC_000441	EPPK1
	FARS2	PNMT	GORAB		E2F8	XLOC_000670	ERBB4
	FAS	PPAN	GPATCH4		EBLN2	XLOC_000776	ERC2
	FAT4	PPAP2B	GPER		ECEL1	XLOC_001320	ESRP2
	FBLN5	PPAPDC3	GPRASP2		EFEMP1	XLOC_001357	EXD3
	FBXL12	PPEF1	GPRC5B		EFEMP2	XLOC_001373	EXT1
	FBXO33	PRAMEF13	GRASP		EFR3B	XLOC_001453	EXTL3
	FBXO9	PRKCB	GRPEL1		EHBP1	XLOC_001515	EZH1
	FCF1	PRPH	GRWD1		EIF2C3	XLOC_001856	F3
	FEN1	PRSS35	GTF2E1		EIF3L	XLOC_002063	FA2H
	FER	PRSS8	GTF3C1		EIF4A2	XLOC_002140	FAAH2
	FERMT2	PSMB9	GUCY1B3		EIF4G3	XLOC_002283	FADS3
	FGF13	PSME1	GYG2		ELK3	XLOC_002581	FAM102A
	FGFR1OP	PTBP1	H2AFJ		EML2	XLOC_002616	FAM110C
	FKBP15	PTGDS	HCST		EML6	XLOC_002779	FAM114A1
	FKRP	PTX3	HDX		EMP3	XLOC_002900	FAM125B
	FLJ37798	RAB39B	HELQ		EMX1	XLOC_003165	FAM129B
	FLJ42627	RAB3C	HERPUD1		ENO2	XLOC_003405	FAM131A
	FLJ45248	RAC2	HEY2		ENSA	XLOC_004452	FAM131C
	FLJ45340	RANBP6	HHLA3		ENTPD4	XLOC_004680	FAM132A
	FLRT3	RBFOX3	HIST1H1D		EP400NL	XLOC_004774	FAM150B
	FLVCR1	RBM47	HIST1H2AB		EPAS1	XLOC_005051	FAM175B
	FNDC1	RCBTB2	HIST1H2AE		EPCAM	XLOC_005633	FAM185A
	FNIP1	RELL2	HIST1H3J		EPHA2	XLOC_005690	FAM189A1
	FNTB	RHEBL1	HLA-DMA		EPPK1	XLOC_005737	FAM189A2

Supplementary Table 1

pIND-CNOT1 AND 2 AND 3 +Dox / pIND-Luci +Dox, > 2 fold up	pIND-CNOT1 +Dox / pIND-Luci +Dox, > 2 fold up	pIND-CNOT2 +Dox / pIND-Luci +Dox, > 2 fold up	pIND-CNOT3 +Dox / pIND-Luci +Dox, > 2 fold up	pIND-CNOT1 AND 2 AND 3 +Dox / pIND-Luci +Dox, > 2 fold down	pIND-CNOT1 +Dox / pIND-Luci +Dox, > 2 fold down	pIND-CNOT2 +Dox / pIND-Luci +Dox, > 2 fold down	pIND-CNOT3 +Dox / pIND-Luci +Dox, > 2 fold down
	FOXD4	RHOT1	HLA-DMB		EPS8L1	XLOC_006144	FAM20B
	FOXG1	RIBC1	HLA-DOA		ERBB4	XLOC_006419	FAM26F
	FOXN4	RIIAD1	HLA-DPB1		ERLEC1	XLOC_006844	FAM43A
	FOXRED1	RINL	HLA-DRB1		ERLIN2	XLOC_007855	FAM46C
	FPGS	RNASE4	HLA-DRB3		ERMP1	XLOC_008000	FAM49A
	FRMD6	RNF43	HLA-DRB5		ERRFI1	XLOC_008005	FAM57B
	FTSJ2	RNFT1	HMOX1		ESRP2	XLOC_008586	FAM69B
	FUBP1	RPS6KL1	HNRNPM		EXD3	XLOC_008652	FAM72A
	FUS	RSPH3	HNRPDL		EZH1	XLOC_008984	FAM83G
	FUT1	RTN1	HOMEZ		F11R	XLOC_009114	FAM84B
	FUT8	S100A2	HOOK2		FA2H	XLOC_009451	FBLIM1
	FXN	S100A4	HOXC10		FADS3	XLOC_009628	FBLN2
	FZD8	SALL4	HOXC12		FAM110C	XLOC_010112	FBXW2
	FZR1	SARDH	HPSE		FAM114A1	XLOC_010167	FBXW5
	GABBR2	SCARA3	HSD17B10		FAM129A	XLOC_011306	FCRL5
	GABPB2	SCN4B	HSD17B14		FAM131A	XLOC_011407	FER1L4
	GAGE2B	SDSL	HSD3B7		FAM131C	XLOC_011645	FEZ1
	GAL3ST1	SEPP1	HSP90AB5P		FAM132A	XLOC_011984	FGF9
	GALC	SFN	HSPA8		FAM132B	XLOC_012338	FGFR1
	GALNT1	SFTA3	HSPH1		FAM150A	XLOC_012586	FGFRL1
	GALNT12	SH2D3A	HYAL3		FAM150B	XLOC_012678	FHIT
	GAS1	SH2D3C	HYLS1		FAM161A	XLOC_012829	FJX1
	GBA	SHROOM4	IFI35		FAM175A	XLOC_012848	FKBP8
	GBAP1	SLC10A4	IFIH1		FAM175B	XLOC_012895	FLJ31485
	GCC1	SLC16A2	IFIT1		FAM183A	XLOC_013154	FLJ31662
	GCLC	SLC16A4	IFRD1		FAM189A1	XLOC_013162	FLJ37035
	GEMIN2	SLC22A17	IFT46		FAM189A2	XLOC_013282	FLJ37453
	GFER	SLC25A21	IFT81		FAM20B	XLOC_013434	FLJ41350
	GFM1	SLC27A2	IGFBP5		FAM20C	XLOC_013449	FLJ42351
	GIT2	SLC27A6	IGFLR1		FAM26F	XLOC_013679	FLJ43315
	GLMN	SLC37A1	IGSF9		FAM46C	XLOC_013853	FLJ44342
	GLOD4	SLC8A1	IL17D		FAM49A	XLOC_013981	FLJ46906
	GLT8D2	SNAI2	IL20RB		FAM54B	XLOC_l2_000339	FLNB
	GNAS-AS1	SNORA43	ILKAP		FAM84B	XLOC_l2_000791	FOXF1
	GNG11	SNX18	INA		FAM89A	XLOC_l2_003039	FPGT-TNNI3K
	GNG4	SNX32	INPP4A		FANK1	XLOC_l2_003602	FSTL3
	GPER	SP5	INTS2		FARP1	XLOC_l2_004168	FYCO1
	GPHN	SPATA20	IQCE		FBLIM1	XLOC_l2_005997	FZD10
	GPR135	SPATA6	ISG20		FBLN1	XLOC_l2_006013	FZD5
	GPRASP2	SPDYC	ISL1		FBLN2	XLOC_l2_007585	FZD6
	GPRIN1	SPEF1	ITGA1		FBXO2	XLOC_l2_009501	G0S2
	GPT2	SPIN3	ITGA8		FBXO24	XLOC_l2_009572	GAB2
	GRPEL1	SPINK2	ITGB4		FBXO27	XLOC_l2_011145	GABARAPL1
	GSC	SPINK5	ITPKB		FER1L4	XLOC_l2_011415	GADD45A
	GTF2E1	SPON2	IZUMO1		FEZ2	XLOC_l2_012902	GADD45G
	GTF2H3	SPRYD7	JAKMIP1		FGF9	XLOC_l2_013001	GALE
	GTF2H4	SPTBN1	JAKMIP2		FHIT	XLOC_l2_013145	GALNT6
	GTF3C1	SRCRB4D	JAM2		FHL3	XLOC_l2_013242	GAS2L3
	GUCY1B2	SSBP3	JMJD1C		FKBP1B	XLOC_l2_013458	GAS6
	H2AFJ	SSPN	JMJD4		FKBP8	XLOC_l2_014504	GATA2
	H2AFX	ST6GALNAC2	KCNA6		FLI1	XLOC_l2_014505	GATA3
	H2AFY2	ST7-AS1	KCNC3		FLJ20444	XLOC_l2_014931	GATA5
	HAUS7	STAC2	KCNN2		FLJ31485	XRRA1	GATAD2B
	HCG18	STARD8	KCNRG		FLJ37453	YPEL1	GBA2
	HCST	STEAP1	KCP		FLJ42351	YWHAE	GBGT1
	HDGFL1	STEAP1B	KCTD1		FLJ44342	ZBTB16	GDF15
	HDX	STEAP2	KCTD18		FLJ45983	ZBTB41	GDNF
	HEATR3	STK10	KDM4D		FLNA	ZDHHC23	GFRA2
	HEATR5A	STXBP6	KHDC1		FLNC	ZKSCAN4	GJB2
	HIF1A	SULT2B1	KIAA0408		FLYWCH1	ZNF10	GLA
	HIST1H2AB	SUSD1	KIAA0415		FMNL2	ZNF136	GLDC
	HIST1H2AG	SYCE3	KIAA0753		FNBP1L	ZNF14	GLYR1
	HIST1H2AI	SYTL1	KIAA1009		FOXF1	ZNF141	GMEB1
	HIST1H2BC	TAC3	KIAA1737		FOXL1	ZNF157	GNRH1
	HIST1H2BF	TACC2	KIDINS220		FPGT-TNNI3K	ZNF160	GNRHR2
	HIST1H2BG	TAP1	KIF25		FREM2	ZNF177	GOLGA2
	HIST1H2BH	TBC1D8B	KIF5A		FRMD5	ZNF222	GOLGA2P5
	HIST1H2BI	TEKT2	KLF5		FSTL3	ZNF230	GOLGA4
	HIST1H2BK	TERT	KLK8		FTSJD2	ZNF234	GOLGA8A
	HIST1H2BM	TEX19	KLRG2		FUK	ZNF250	GOLGA8E
	HIST1H2BN	TFPI2	KRCC1		FXR1	ZNF264	GPC2
	HIST1H3D	TGFB1I1	KRT222		FXYD6	ZNF28	GPM6A
	HIST1H3F	THAP7-AS1	KRTAP19-1		FZD10	ZNF322	GPR1
	HIST1H3J	THAP8	KRTAP4-11		FZD6	ZNF34	GPR107
	HIST2H2BF	TLCD1	LACTB2		G0S2	ZNF343	GPR124
	HIST3H2BB	TLX2	LAMTOR3		GAB2	ZNF345	GPR64
	HLA-DMA	TMEM102	LBX2		GABARAPL1	ZNF35	GPSM1
	HLA-DOA	TMEM173	LCLAT1		GADD45A	ZNF41	GRAMD4
	HLA-DRB3	TMEM22	LCMT2		GADD45G	ZNF441	GRHL1
	HLA-DRB5	TMEM56-RWDD3	LDHC		GALE	ZNF468	GSDMB
	HNRNPM	TNFAIP2	LEO1		GALNS	ZNF497	GSN
	HNRPDL	TNNI3	LINC00338		GALNT6	ZNF519	GSR
	HOMEZ	TNS1	LINC00340		GANAB	ZNF532	GYLTL1B
	HOTAIR	TOB2P1	LINC00461		GAS6	ZNF585A	H19
	HOXA7	TP53I3	LINGO3		GATA3	ZNF585B	HABP4
	HOXC12	TRIM7	LMBR1		GATA5	ZNF594	HAGHL
	HPS6	TRPV2	LNP1		GATA6	ZNF600	HAND1
	HRAS	TSPAN10	LOC100128714		GBGT1	ZNF654	HAPLN2
	HS6ST2	TSPAN12	LOC100130093		GCA	ZNF670	HBEGF
	HSD17B10	TTC39A	LOC100130776		GDF15	ZNF678	HBZ
	HSPA8	TTC8	LOC100131262		GDNF	ZNF696	HCG11
	HSPH1	TUBB3	LOC100132147		GEM	ZNF710	HDAC5
	HTT	TXNRD2	LOC100132356		GFRA1	ZNF717	HDHD3
	HYAL3	TXNRD3	LOC100134937		GFRA2	ZNF761	HES2
	HYLS1	UBE2L6	LOC100144603		GGPS1	ZNF767	HES5
	IBA57	UBR5	LOC100233156		GGTLC2	ZNF782	HEXIM1
	IFI35	UFSP1	LOC100240735		GJB2	ZNF843	HIVEP2
	IFT46	UNC5C	LOC100286925		GK	ZNF85	HKR1
	IL17D	USH1G	LOC100287063		GLDC	ZRANB1	HMBOX1
	IL20RB	USP8	LOC100287482		GLI1	ZSCAN18	HMGA1P4
	IL27RA	VAV1	LOC100293962		GLS		HMGB3
	INA	VAV3	LOC100505481		GMCL1		HOOK3
	INPP5D	VAX1	LOC100505495		GMEB1		HOTAIRM1
	IPO13	VAX2	LOC100505554		GNB1		HOXB3
	IPO4	VSIG10L	LOC100505773		GNB4		HOXB5
	IQCE	WBSCR27	LOC100505908		GNE		HOXB6
	ISCA2	WIBG	LOC100506123		GNPDA2		HOXB8
	ITGA1	WNT10B	LOC100506125		GNRH1		HOXB9
	ITGA8	XLOC_000167	LOC100506161		GNRHR2		HOXD10
	ITGB4	XLOC_000175	LOC100506302		GOLGA2		HOXD8
	ITIH4	XLOC_000190	LOC100506392		GOLGA6L10		HP07349
	IZUMO1	XLOC_000743	LOC100506451		GOLGA8A		HRK
	JAKMIP1	XLOC_000822	LOC100506831		GPC2		HS6ST3
	JAM2	XLOC_000883	LOC100506848		GPD2		HSPA1A
	JDP2	XLOC_001532	LOC100507047		GPM6A		HSPB8
	JMJD4	XLOC_002026	LOC100507146		GPR1		HTR2A
	KCNA6	XLOC_005764	LOC100507437		GPR124		IDS
	KCNC3	XLOC_006544	LOC100507637		GPR137B		IER2
	KCNJ14	XLOC_007052	LOC100510710		GPR35		IER3

Supplementary Table 1

pIND-CNOT1 AND 2 AND 3 +Dox / pIND-Luci +Dox, > 2 fold up	pIND-CNOT1 +Dox / pIND-Luci +Dox, > 2 fold up	pIND-CNOT2 +Dox / pIND-Luci +Dox, > 2 fold up	pIND-CNOT3 +Dox / pIND-Luci +Dox, > 2 fold up	pIND-CNOT1 AND 2 AND 3 +Dox / pIND-Luci +Dox, > 2 fold down	pIND-CNOT1 +Dox / pIND-Luci +Dox, > 2 fold down	pIND-CNOT2 +Dox / pIND-Luci +Dox, > 2 fold down	pIND-CNOT3 +Dox / pIND-Luci +Dox, > 2 fold down
	KCNN2	XLOC_007348	LOC100652764		GRAMD1C		IER5
	KCNRG	XLOC_007433	LOC151534		GRAMD4		IFI27
	KCTD18	XLOC_007769	LOC153546		GRB14		IGF1R
	KDM3B	XLOC_009147	LOC170425		GREB1		IGF2
	KDM4D	XLOC_009509	LOC282997		GREB1L		IGFBP6
	KEAP1	XLOC_010495	LOC284939		GRHL1		IGFBP7
	KHDC1	XLOC_010952	LOC339803		GSDMB		IGFBPL1
	KIAA0317	XLOC_011305	LOC400236		GSR		IGIP
	KIAA0408	XLOC_011344	LOC400958		GYPC		IGSF21
	KIAA0415	XLOC_013301	LOC401068		H19		IL10RA
	KIAA0586	XLOC_014063	LOC401431		HAGHL		IL11
	KIAA0753	XLOC_014397	LOC440149		HAND1		IL15RA
	KIAA0922	XLOC_I2_000217	LOC642366		HBZ		IL17RD
	KIAA1009	XLOC_I2_001138	LOC642852		HDAC10		IL28RA
	KIAA1737	XLOC_I2_002433	LOC643072		HES2		ILF3
	KIAA1967	XLOC_I2_004371	LOC644145		HES4		IMMP2L
	KIF5A	XLOC_I2_010330	LOC644450		HES5		INF2
	KLC1	XLOC_I2_011204	LOC645195		HES6		INHBB
	KLHDC2	XLOC_I2_011649	LOC645638		HIST1H4F		INPP1
	KLHDC5	XLOC_I2_013267	LOC646778		HIST1H4I		INPP5D
	KRCC1	XLOC_I2_015632	LOC646999		HIST1H4J		INPP5E
	KRI1	ZBTB25	LOC648987		HIST1H4K		INSL3
	KRR1	ZCCHC12	LOC730236		HIST2H4B		INSR
	KRT222	ZCWPW1	LOC92659		HIVEP2		INVS
	KRT8	ZNF251	LOX		HLA-DPA1		IQSEC2
	KRT8P12	ZNF280D	LPHN3		HMBOX1		IRX4
	L3MBTL3	ZNF498	LPIN2		HMGCL		ISYNA1
	LARS2	ZNF667	LRIF1		HMHA1		ITGA6
	LBX2	ZNF837	LRP11		HNRNPU-AS1		ITGB1
	LFNG	ZSWIM5	LRRC3		HOMER3		ITPR3
	LGMN		LRRC40		HOOK3		ITPRIP
	LINC00094		LRRC56		HOTAIRM1		ITPRIPL2
	LINC00174		LRRC61		HOXA4		JAG2
	LINC00338		LRRIQ3		HOXB3		JPH3
	LINC00461		LRTOMT		HOXB5		JUN
	LINC00476		LSM14B		HOXB6		JUNB
	LINGO1		LSS		HOXB8		JUND
	LINGO3		LTN1		HOXB9		KATNAL1
	LIPE		LXN		HOXD10		KAZALD1
	LIPT2		LY6G6D		HOXD9		KAZN
	LMBR1		LYAR		HPCAL1		KBTBD11
	LNP1		LZTFL1		HSD17B1		KCNC4
	LOC100128361		MAGI2-AS3		HSPA1A		KCNJ18
	LOC100128788		MAGIX		HSPB8		KGFLP2
	LOC100129129		MAP1A		HSPG2		KHDRBS3
	LOC100130093		MAP2K6		HTR2A		KHNYN
	LOC100130776		MAPK8IP2		human		KIAA0146
	LOC100131262		MAPRE2		ICAM2		KIAA0182
	LOC100132147		MARVELD2		ICAM4		KIAA0226
	LOC100132356		MAT2B		ICAM5		KIAA1191
	LOC100132815		MAVS		ID3		KIAA1919
	LOC100134937		MBLAC1		IDH2		KIF13B
	LOC100144603		MBLAC2		IDS		KIF20B
	LOC100233156		MDGA2		IER2		KIFC2
	LOC100240735		MDP1		IER3		KIR3DL1
	LOC100286925		MECOM		IER5L		KLF11
	LOC100287063		MED17		IFI27		KLF4
	LOC100289341		MED20		IFI6		KLF6
	LOC100293962		MEF2C		IFITM1		KLF9
	LOC100505487		MEIS1		IFITM2		KLHL17
	LOC100505495		METTL1		IFITM3		KLHL24
	LOC100505554		METTL4		IFITM4P		KLHL5
	LOC100506302		METTL7B		IGF1R		KLK1
	LOC100506411		MFSD1		IGF2BP2		KREMEN2
	LOC100506451		MFSD5		IGFBP6		KRTAP19-2
	LOC100506465		MGC12488		IGFBPL1		LAMA3
	LOC100506659		MGC4473		IGIP		LAMB2P1
	LOC100506694		MIRLET7BHG		IGSF21		LAMC3
	LOC100506714		MIS18BP1		IGSF9B		LATS2
	LOC100506848		MKLN1		IKZF2		LBH
	LOC100506930		MOK		IL10RA		LCAT
	LOC100507047		MPZL3		IL11		LCE1D
	LOC100507437		MRPL1		IL15		LCN15
	LOC100507739		MRPL2		IL15RA		LCOR
	LOC100510710		MRPL21		IL17RD		LCORL
	LOC100630918		MRPL46		IL28RA		LEAP2
	LOC100652764		MSRA		IMMP2L		LGALS3
	LOC100652843		MSTO1		IMMT		LIMS2
	LOC100652897		MTBP		INSL3		LINC00085
	LOC100652987		MTERF		IQSEC2		LINC00115
	LOC100653004		MTG1		IRF2BP2		LINC00294
	LOC100653149		MUM1		IRF2BPL		LINC00339
	LOC148189		MUM1L1		IRX4		LMX1B
	LOC153546		MVK		ISYNA1		LOC100128242
	LOC158435		MYO5C		ITGA6		LOC100128822
	LOC170425		MYOF		ITGAV		LOC100128977
	LOC282997		NAGPA		ITGB1		LOC100129034
	LOC284939		NAP1L2		ITPKA		LOC100129461
	LOC386758		NAP1L3		ITPKB		LOC100129550
	LOC400027		NAPRT1		ITPR1		LOC100130015
	LOC400236		NDC80		JAK3		LOC100130027
	LOC401068		NDNL2		JHDM1D		LOC100130691
	LOC401431		NEAT1		JUN		LOC100131107
	LOC440149		NEK1		KALRN		LOC100131564
	LOC642366		NEK3		KANK1		LOC100132163
	LOC644145		NEURL2		KATNAL1		LOC100132832
	LOC644450		NEXN		KAZALD1		LOC100132909
	LOC644656		NFKB1		KAZN		LOC100169752
	LOC644727		NHEJ1		KBTBD10		LOC100270746
	LOC645676		NKX3-1		KCMF1		LOC100272228
	LOC646762		NOL6		KCNB2		LOC100287437
	LOC646778		NOS2		KCNC4		LOC100287506
	LOC646999		NOS3		KCNS3		LOC100288420
	LOC648987		NOVA1		KCTD19		LOC100289026
	LOC728613		NOVA2		KDELR3		LOC100289137
	LOC728903		NPHP1		KDM3A		LOC100289187
	LOC729013		NPHP3		KGFLP1		LOC100292905
	LOC729860		NR0B1		KHDRBS3		LOC100505478
	LOC92659		NRAS		KIAA0090		LOC100505601
	LOC93622		NRG2		KIAA0182		LOC100505641
	LOX		NTHL1		KIAA0226		LOC100505648
	LPCAT1		NTRK2		KIAA0319L		LOC100505679
	LPCAT2		NUDT16P1		KIAA0913		LOC100505730
	LPHN3		NUDT17		KIAA0930		LOC100505771
	LPIN2		NUDT4		KIAA1244		LOC100505876
	LRIF1		NUP37		KIAA1462		LOC100505894
	LRP10		NUPR1		KIF13B		LOC100505912
	LRP11		NXF1		KIF1A		LOC100506130
	LRR1		NYNRIN		KIF20B		LOC100506251

Supplementary Table 1

pIND-CNOT1 AND 2 AND 3 +Dox / pIND-Luci +Dox, > 2 fold up	pIND-CNOT1 +Dox / pIND-Luci +Dox, > 2 fold up	pIND-CNOT2 +Dox / pIND-Luci +Dox, > 2 fold up	pIND-CNOT3 +Dox / pIND-Luci +Dox, > 2 fold up	pIND-CNOT1 AND 2 AND 3 +Dox / pIND-Luci +Dox, > 2 fold down	pIND-CNOT1 +Dox / pIND-Luci +Dox, > 2 fold down	pIND-CNOT2 +Dox / pIND-Luci +Dox, > 2 fold down	pIND-CNOT3 +Dox / pIND-Luci +Dox, > 2 fold down
	LRRC3		OBFC2A		KIFC2		LOC100506268
	LRRC56		OLIG1		KIR3DL1		LOC100506518
	LRRC61		OSGEP		KLF12		LOC100506543
	LRRIQ3		OTUD1		KLF2		LOC100506548
	LRRN2		PACS2		KLF6		LOC100506586
	LRTOMT		PARP10		KLF7		LOC100506662
	LY6G6D		PAX1		KLHDC9		LOC100506710
	MAEA		PAX3		KLHL17		LOC100506778
	MAGOHB		PBX1		KLHL24		LOC100506866
	MAPK1IP1L		PBX2		KLHL5		LOC100506950
	MAPK8IP2		PCDH7		KLHL7		LOC100507153
	MAPRE2		PCDH9		KLK1		LOC100507331
	MARCH5		PCDHGA8		KRTAP19-1		LOC100507353
	MARVELD1		PCYT2		KRTAP19-2		LOC100507363
	MARVELD2		PDE3B		KRTAP20-2		LOC100507376
	MAX		PDE6D		KRTAP4-3		LOC100507420
	MBD3		PDGFRL		KRTAP9-6		LOC100507468
	MBLAC1		PELI3		LAMB2P1		LOC100507508
	MBLAC2		PEPD		LANCL1		LOC100508120
	MBNL1		PER2		LARP1B		LOC100508995
	MBTPS2		PEX13		LATS2		LOC100652741
	MCPH1		PHACTR3		LBH		LOC100652758
	MDGA2		PHF12		LCORL		LOC100652769
	MDM2		PIBF1		LGALS3		LOC100652913
	MDP1		PIGB		LGALS9C		LOC100653021
	MECOM		PIGG		LHX1		LOC100653030
	MED16		PIGW		LIAS		LOC100653206
	MED20		PIH1D2		LIFR		LOC100653323
	MED6		PIN1P1		LIMCH1		LOC113230
	MEIS2		PIP4K2C		LIMK1		LOC149351
	METTL1		PIR		LIMS1		LOC200830
	METTL17		PLA2G7		LINC00115		LOC202781
	METTL7B		PLAGL1		LINC00173		LOC203274
	MFAP3		PLEKHA4		LINC00294		LOC221442
	MFSD1		PLEKHG4		LINC00340		LOC254057
	MFSD5		PLIN2		LMCD1		LOC256880
	MGAT2		PLIN3		LMTK3		LOC284219
	MGAT5B		PLTP		LOC100127983		LOC284408
	MGC4473		PMP22		LOC100128242		LOC284628
	MIRLET7BHG		PNMA5		LOC100128977		LOC285095
	MIS18BP1		PNMA6C		LOC100129387		LOC286052
	MKLN1		PNMAL1		LOC100130015		LOC286109
	MMP9		PNMT		LOC100130691		LOC286161
	MOK		POLR1C		LOC100131107		LOC340335
	MORC4		POLR2C		LOC100131434		LOC348120
	MPZL3		POLR3C		LOC100131564		LOC388780
	MRM1		POP1		LOC100131607		LOC389493
	MRP63		PPAN		LOC100132163		LOC389765
	MRPL1		PPAP2B		LOC100132832		LOC390940
	MRPL52		PPEF1		LOC100169752		LOC400550
	MRPS7		PPHLN1		LOC100272228		LOC400657
	MSL3		PPP3CA		LOC100287082		LOC401127
	MSRA		PRKCB		LOC100287437		LOC402160
	MSTO1		PRLHR		LOC100287506		LOC439950
	MTERF		PRMT5		LOC100288092		LOC440288
	MTFP1		PRPF3		LOC100288420		LOC440944
	MTG1		PRPSAP2		LOC100289137		LOC441666
	MTHFD1		PRR19		LOC100292909		LOC441736
	MUDENG		PRSS35		LOC100293704		LOC642311
	MUM1		PSMB9		LOC100505478		LOC642826
	MYBBP1A		PSMC4		LOC100505601		LOC642924
	MYOD1		PSME1		LOC100505616		LOC644277
	NAA25		PSMG1		LOC100505648		LOC645321
	NADKD1		PSRC1		LOC100505679		LOC645722
	NAGPA		PTBP1		LOC100505695		LOC647979
	NAP1L2		PTGDS		LOC100505730		LOC727721
	NAP1L3		PWP1		LOC100505771		LOC727982
	NAT10		PYCRL		LOC100505787		LOC728052
	NDC80		RAB36		LOC100505869		LOC728558
	NDNL2		RAB39B		LOC100505876		LOC728730
	NDRG2		RAB3C		LOC100505894		LOC728743
	NEK1		RAB9B		LOC100505905		LOC728802
	NEK9		RABL3		LOC100505912		LOC728978
	NEURL2		RABL5		LOC100505937		LOC729041
	NFKB1		RANBP6		LOC100506130		LOC729444
	NGDN		RARS		LOC100506295		LOC84856
	NHEJ1		RASIP1		LOC100506334		LOC96610
	NIN		RBFOX3		LOC100506421		LONRF2
	NIP7		RBKS		LOC100506543		LONRF3
	NKX3-1		RBM33		LOC100506548		LOXL2
	NOBOX		RBM45		LOC100506586		LOXL4
	NOL8		RBM8A		LOC100506710		LRP12
	NOP14		RCBTB2		LOC100506748		LRP8
	NOS2		RCOR3		LOC100506866		LRRC26
	NOS3		RELL2		LOC100506870		LRRC8A
	NOVA1		RFX2		LOC100506950		LRRC8E
	NOVA2		RGAG4		LOC100506985		LRRFIP1
	NPAT		RGS20		LOC100507062		LRSAM1
	NPFF		RHEB		LOC100507153		LZIC
	NPTX2		RIBC1		LOC100507173		LZTS1
	NR0B1		RIIAD1		LOC100507284		MAFB
	NRXN2		RIMBP3		LOC100507331		MAFK
	NSD1		RIOK2		LOC100507353		MAMDC4
	NT5DC1		RLN2		LOC100507363		MAN2A2
	NTRK2		RNF138P1		LOC100507376		MAP3K13
	NUBPL		RNFT2		LOC100507412		MAP3K14
	NUDT16		RNU11		LOC100507420		MAP3K15
	NUDT16P1		ROPN1L		LOC100507421		MAP6D1
	NUDT17		RPAP2		LOC100507473		MAP7
	NUDT4		RPL22L1		LOC100507475		MAP7D2
	NUMB		RRP15		LOC100507645		MAPK4
	NYNRIN		RRP7B		LOC100507918		MAPK8IP1
	OGDHL		RRP9		LOC100652741		MAPT
	OLIG1		RSG1		LOC100652769		MARCH2
	ORC5		RSPH3		LOC100653021		MARCH8
	OSBPL2		RTDR1		LOC100653257		MAST2
	OSGEP		RTN1		LOC100653323		MASTL
	OTUD1		RWDD2B		LOC144481		MAZ
	PACS2		S100A4		LOC202781		MBP
	PACSIN1		S100P		LOC221442		MBTPS1
	PAQR4		SARS2		LOC256880		MCCC1
	PAQR9		SCAMP5		LOC283174		MCF2L-AS1
	PARP10		SCAND1		LOC284219		MCM9
	PAX1		SCARA3		LOC284513		MCOLN3
	PAX9		SCLT1		LOC284757		MDM4
	PCBD2		SCN4B		LOC340335		MEF2BNB
	PCDH9		SEMA3C		LOC348120		MEGF9
	PCDHGA8		SEPP1		LOC388588		METRNL
	PCSK7		SERPINI1		LOC388780		MFAP3L

Supplementary Table 1

pIND-CNOT1 AND 2 AND 3 +Dox / pIND-Luci +Dox, > 2 fold up	pIND-CNOT1 +Dox / pIND-Luci +Dox, > 2 fold up	pIND-CNOT2 +Dox / pIND-Luci +Dox, > 2 fold up	pIND-CNOT3 +Dox / pIND-Luci +Dox, > 2 fold up	pIND-CNOT1 AND 2 AND 3 +Dox / pIND-Luci +Dox, > 2 fold down	pIND-CNOT1 +Dox / pIND-Luci +Dox, > 2 fold down	pIND-CNOT2 +Dox / pIND-Luci +Dox, > 2 fold down	pIND-CNOT3 +Dox / pIND-Luci +Dox, > 2 fold down
	PCYT2		SESN2		LOC389634		MFI2
	PDE3B		SETD6		LOC390940		MFSD6
	PELI3		SEZ6L2		LOC400128		MGAT4A
	PEPD		SFPQ		LOC400657		MGC12982
	PEX6		SFTA3		LOC400958		MGC16121
	PGBD2		SFXN2		LOC401022		MGC23284
	PGP		SH2B2		LOC402160		MGC2752
	PHF12		SH3BGR		LOC439911		MIR221
	PHYHD1		SH3PXD2B		LOC439950		MLXIPL
	PIGQ		SHROOM4		LOC440300		MMP15
	PIGW		SIK3		LOC497257		MOB3B
	PIH1D2		SIRPA		LOC642311		MOSPD1
	PILRA		SLC13A4		LOC642924		MOXD1
	PIN1P1		SLC16A4		LOC644339		MST1
	PIP4K2C		SLC22A17		LOC645722		MTHFSD
	PITPNA		SLC25A13		LOC727982		MTUS1
	PKD2		SLC25A46		LOC728061		MUCL1
	PLA2G7		SLC27A6		LOC728730		MXD3
	PLEKHA4		SLC30A1		LOC728802		MXRA8
	PLSCR1		SLC35D1		LOC730102		MYCL1
	PLTP		SLC35E3		LOC96610		MYLIP
	PNMA5		SLC37A1		LOH12CR1		MYO1F
	PNMA6C		SLC38A7		LONRF3		MYOM2
	PNMT		SLC39A6		LOXL2		MYT1
	PNN		SLC50A1		LPIN1		MZF1
	PNPLA7		SLC7A11		LPPR3		MZT2A
	POC1A		SLC7A8		LRP1		NANOS3
	POLA1		SLC8A1		LRP8		NANP
	POLE4		SLC9A9		LRRC36		NAT6
	POLR3C		SMG6		LRRFIP1		NCAM1
	POM121		SMOX		LRSAM1		NCS1
	POMT2		SMPDL3B		LY6K		ND6
	PPAP2B		SNAI2		LYPD1		NDRG1
	PPARD		SNAP29		LZIC		NEDD4L
	PPEF1		SNAR-F		LZTS1		NEFL
	PPHLN1		SNAR-G1		MAD2L2		NEFM
	PPME1		SNAR-G2		MAF		NEIL2
	PPP1R13B		SNORA28		MAFB		NEK6
	PPP2R1A		SNORA33		MAFF		NELF
	PRKACB		SNORA45		MAFK		NELL2
	PRKCB		SNORA48		MAN2A2		NFATC1
	PRLHR		SNORA62		MAN2B1		NFIC
	PRMT5		SNORA67		MAOA		NGFR
	PRPF3		SNORD12C		MAP3K13		NHLH2
	PRR19		SNX4		MAP3K14		NHLRC2
	PRSS35		SNX8		MAP3K15		NHSL2
	PSMA6		SOAT1		MAP7D2		NINJ1
	PSMB9		SPAG16		MAPK14		NIPA1
	PSMC1		SPARC		MAPK4		NIPAL1
	PSMC4		SPATA20		MAPT		NIPSNAP3A
	PSMC6		SPATA5L1		MARCH2		NISCH
	PTBP1		SPATA6		MARCH8		NKX6-2
	PTGDS		SPATS2L		MAST3		NNT
	PYCRL		SPEF1		MASTL		NOTCH1
	R3HCC1		SPIN3		MBD5		NOXA1
	RAB15		SPINK5		MBP		NPDC1
	RAB36		SPOCD1		MCEE		NPNT
	RAB39B		SPRYD4		MCF2L-AS1		NPTX1
	RAB3C		SPTBN1		MCL1		NPY
	RAB9B		SPTBN4		MCU		NR1D2
	RABL3		SRD5A3		MDM4		NRARP
	RAD51D		SRP19		MED14		NSL1
	RALGAPA1		SRPX		MEGF8		NT5DC3
	RANBP10		SRSF12		MERTK		NT5M
	RANBP17		SSBP3		MESP1		NTN4
	RANBP6		SSPN		METRN		NTNG2
	RASEF		SSR3		METRNL		NUCB1
	RBFOX3		SSX2IP		METTL10		NUP210
	RBM33		ST6GALNAC3		MEX3A		NUP62CL
	RCBTB2		ST7L		MFAP2		OBFC1
	RCC1		STAC2		MFAP3L		OCA2
	RCOR1		STAG1		MFGE8		OCEL1
	RDH10		STARD8		MFI2		ODF3B
	RELL2		STAT6		MFSD10		ODZ1
	REM2		STEAP1		MFSD2A		ONECUT1
	REXO1L1		STEAP1B		MFSD6		ONECUT3
	RFX2		STK33		MGAT4A		OPA1
	RFX7		STK36		MGC16121		OR5D14
	RGS20		STXBP6		MICAL2		OR5L2
	RHEB		SUGT1		MIR221		OR7E14P
	RIIAD1		SUGT1P1		MIR22HG		OTUD7B
	RIMBP3		SULT1A2		MKL1		OVGP1
	RMI1		SULT2B1		MKNK2		P2RY1
	RNASEH2B		SYBU		MLLT11		PABPC4L
	RNF121		SYNCRIP		MLLT3		PACRGL
	RNF138P1		SYNRG		MOSPD1		PAK2
	RNF20		TAC3		MPP3		PALM2
	RNF31		TACC2		MRC2		PAN2
	RNMTL1		TAP1		MSH5		PANK4
	RNU11		TAPBP		MSRB3		PAPLN
	ROPN1L		TBC1D8B		MST1		PARD6G
	RPL22L1		TCEAL6		MT1B		PASK
	RPP30		TCP1		MT1E		PAX7
	RPP40		TEKT2		MT1H		PBK
	RPS6KA4		TERC		MT1L		PBX3
	RPS6KL1		TERT		MT1X		PCDH10
	RPTOR		TEX19		MT2A		PCIF1
	RPUSD2		TFPI2		MTL5		PCK1
	RRP9		TFPT		MTUS1		PCYT1A
	RRS1		THAP10		MUCL1		PDCD2
	RTN1		THAP7-AS1		MXI1		PDCL
	RWDD2B		THAP8		MXRA8		PDGFA
	SAFB2		THBS4		MYOM2		PDIK1L
	SAP30		THTPA		MYT1		PDLIM1
	SAP30BP		TLCD1		MZF1		PDLIM3
	SART1		TMEM102		MZT2A		PDLIM4
	SBDSP1		TMEM145		NANP		PDS5B
	SCAF4		TMEM165		NAT6		PDXDC2P
	SCAMP5		TMEM173		NBAS		PDXP
	SCARA3		TMEM19		NCAM1		PFKFB3
	SCARNA9		TMEM198		NCBP2		PFKP
	SCLT1		TMEM199		NDRG1		PHLDA3
	SCN4B		TMEM22		NDRG3		PHPT1
	SDAD1		TMEM234		NDUFC2		PHYHD1
	SDSL		TMEM45A		NEFL		PI15
	SEL1L		TMPO		NEFM		PIDD
	SERPINH1		TNFAIP2		NEIL2		PIGL
	SESN2		TNNI3		NEURL		PIGX
	SETD3		TNS1		NFATC1		PIK3CD

Supplementary Table 1

pIND-CNOT1 AND 2 AND 3 +Dox / pIND-Luci +Dox, > 2 fold up	pIND-CNOT1 +Dox / pIND-Luci +Dox, > 2 fold up	pIND-CNOT2 +Dox / pIND-Luci +Dox, > 2 fold up	pIND-CNOT3 +Dox / pIND-Luci +Dox, > 2 fold up	pIND-CNOT1 AND 2 AND 3 +Dox / pIND-Luci +Dox, > 2 fold down	pIND-CNOT1 +Dox / pIND-Luci +Dox, > 2 fold down	pIND-CNOT2 +Dox / pIND-Luci +Dox, > 2 fold down	pIND-CNOT3 +Dox / pIND-Luci +Dox, > 2 fold down
	SETD6		TOB2P1		NFE2L3		PIM3
	SEZ6L2		TP53BP1		NGEF		PKIB
	SFPQ		TP53I3		NGFR		PKN1
	SGMS2		TP53RK		NHLH2		PKN3
	SH3PXD2B		TRA2A		NHSL2		PLA2G16
	SHB		TRIM2		NIPA1		PLA2G4E
	SIK3		TRMT1L		NKX6-2		PLAC1
	SIPA1L1		TRPV1		NOTCH3		PLAT
	SIRPA		TRPV2		NPM3		PLAU
	SIVA1		TSPAN10		NPNT		PLCE1
	SLC13A3		TSPAN12		NPY		PLD1
	SLC16A14		TTC12		NR1D2		PLEKHA1
	SLC16A9		TTC18		NR2C2		PLK2
	SLC22A17		TTC25		NR5A2		PLP2
	SLC23A2		TTC26		NRIP3		PLS1
	SLC25A13		TTC27		NRN1		PLS3
	SLC25A21		TTC39C		NRXN3		PLXND1
	SLC25A46		TTC8		NSL1		PMAIP1
	SLC27A6		TUBA8		NSUN3		PMEPA1
	SLC29A3		TUBGCP5		NTNG2		PON3
	SLC2A6		U2SURP		NUDT2		POU4F3
	SLC35D1		UBAC2-AS1		OAZ3		PPARG
	SLC36A1		UBE2L6		OBFC1		PPM1J
	SLC37A1		UBR5		OBSL1		PPP1R14C
	SLC38A10		UFSP1		OCEL1		PPP1R26
	SLC38A7		UGGT2		ODF3B		PPP1R3F
	SLC6A15		ULBP2		ODZ1		PPP3CB
	SLC7A1		UNC5C		OR5D14		PQLC3
	SLC7A11		USP14		OR5L2		PRAMEF13
	SLC7A8		UTP14A		OSBPL10		PREX1
	SLC9A9		UTP15		OSGEPL1		PRKCH
	SLITRK5		UTP20		OTUD3		PRPH
	SMCHD1		VAV3		OTUD7B		PRR11
	SMEK1		VAX1		PABPC3		PRR25
	SMG6		VAX2		PACRGL		PRRC2B
	SMOX		VPS18		PAFAH2		PRSS12
	SNAI2		VPS33A		PAIP2B		PRUNE2
	SNAP29		VPS52		PALM2		PSAP
	SNAR-F		VSIG10L		PAPLN		psiTPTE22
	SNAR-G1		VTA1		PAQR5		PTAR1
	SNAR-G2		WBSCR27		PARD6B		PTBP3
	SNAR-H		WDR46		PARP8		PTCD3
	SNORA28		WDR61		PAX7		PTCH1
	SNORA33		WDR73		PBK		PTGES2
	SNORA47		WDR78		PBXIP1		PTH2
	SNORA62		WDR81		PC		PTPN21
	SNORA67		WIBG		PCBP4		PTPN3
	SNORA76		WNT10B		PCGF1		PTPRF
	SNRPB2		WTAP		PCK1		PTPRJ
	SNUPN		XLOC_000167		PDE4DIP		PURA
	SNX18		XLOC_000175		PDGFA		PVRL4
	SNX4		XLOC_000190		PDK1		PVT1
	SOBP		XLOC_000505		PDK4		PWWP2B
	SORD		XLOC_000822		PDLIM3		Q5SRJ3
	SPATA20		XLOC_000883		PDLIM4		Q7Z2X8
	SPDYC		XLOC_001532		PDPN		QRFPR
	SPEF1		XLOC_001699		PDSS2		QSOX2
	SPIN3		XLOC_001775		PDXDC2P		R3HDM2
	SPINK5		XLOC_002026		PFKFB3		RAB11FIP1
	SPOCD1		XLOC_002652		PFN4		RAB14
	SPRYD4		XLOC_002921		PHF13		RAB37
	SPRYD7		XLOC_004677		PHLDB1		RAB38
	SPTBN1		XLOC_005764		PI15		RAB3A
	SPTLC2		XLOC_007348		PI4KAP1		RABGAP1
	SRD5A3		XLOC_007433		PI4KAP2		RAD23A
	SRSF8		XLOC_007635		PIAS1		RAD23B
	SSBP3		XLOC_007769		PIGF		RALGPS1
	SSH1		XLOC_008079		PIGL		RASGEF1B
	SSX2IP		XLOC_009147		PIGX		RASL10A
	ST7L		XLOC_009233		PIK3CA		RASSF3
	STAG1		XLOC_009509		PIK3CD		RASSF4
	STARD8		XLOC_011344		PIM3		RBAK
	STAT6		XLOC_011676		PITPNC1		RBBP5
	STEAP1		XLOC_012142		PKDCC		RBFA
	STEAP1B		XLOC_013301		PKIB		RBP1
	STK10		XLOC_013973		PKN1		RBP7
	STXBP5		XLOC_014063		PLA2G16		RBPJ
	STXBP6		XLOC_014288		PLA2G4A		RBPMS
	SUGT1		XLOC_014397		PLA2G4E		RDM1
	SULF1		XLOC_l2_002433		PLAG1		RDX
	SUPT16H		XLOC_l2_003992		PLAT		RETSAT
	SURF2		XLOC_l2_006862		PLAU		RGS12
	SURF6		XLOC_l2_008151		PLCD1		RGS16
	SUSD1		XLOC_l2_009273		PLCE1		RHOB
	SYNCRIP		XLOC_l2_009442		PLEKHA2		RHOBTB2
	SYNGR4		XLOC_l2_011584		PLEKHB1		RHOC
	SYNJ2BP		XLOC_l2_011649		PLEKHG5		RIBC2
	SYNRG		XLOC_l2_013267		PLK2		RIC8B
	SYT17		XLOC_l2_013480		PLP2		RIMS3
	TAP1		XLOC_l2_015632		PLS3		RLTPR
	TAPBP		YLPM1		PMAIP1		RN5-8S1
	TBC1D8B		ZBTB25		PMEL		RNF122
	TBXAS1		ZC3H13		PMM1		RNF19A
	TCP1		ZCWPW1		PMS1		RNF208
	TERC		ZDHHC14		POLR3G		RNF223
	THAP7-AS1		ZFPL1		PON3		RNU6ATAC
	THBS4		ZKSCAN5		POU4F3		RPH3AL
	THEM4		ZMYM3		PPAPDC1B		RPIA
	THRA		ZNF174		PPARA		RPL22
	THTPA		ZNF19		PPARG		RPRML
	TIGD2		ZNF23		PPIE		RPS6KB1
	TLCD1		ZNF251		PPIL3		RUSC2
	TMEM104		ZNF252		PPM1J		RXRA
	TMEM110		ZNF271		PPP1CB		SAP25
	TMEM145		ZNF280D		PPP1R12B		SAT1
	TMEM165		ZNF283		PPP1R18		SATB1
	TMEM173		ZNF425		PPP2R5B		SCAI
	TMEM181		ZNF498		PQLC3		SCARA5
	TMEM19		ZNF512B		PRAMEF13		SDC4
	TMEM198		ZNF608		PRDM16		SDHAP1
	TMEM199		ZNF667		PREPL		SEC14L2
	TMEM216		ZNF688		PRKAB2		SEC16A
	TMEM22		ZNF70		PRKAR1A		SEC31B
	TMEM234		ZSCAN5A		PRKCE		SEL1L3
	TMEM5		ZSWIM5		PRKCH		SELO
	TMEM56-RWDD3				PROS1		SEMA4C
	TMEM63C				PRPF4B		SEMA4D
	TMPO				PRR11		SEMA4G
	TMX1				PRR5-ARHGAP8		SENP5

Supplementary Table 1

pIND-CNOT1 AND 2 AND 3 +Dox / pIND-Luci +Dox, > 2 fold up	pIND-CNOT1 +Dox / pIND-Luci +Dox, > 2 fold up	pIND-CNOT2 +Dox / pIND-Luci +Dox, > 2 fold up	pIND-CNOT3 +Dox / pIND-Luci +Dox, > 2 fold up	pIND-CNOT1 AND 2 AND 3 +Dox / pIND-Luci +Dox, > 2 fold down	pIND-CNOT1 +Dox / pIND-Luci +Dox, > 2 fold down	pIND-CNOT2 +Dox / pIND-Luci +Dox, > 2 fold down	pIND-CNOT3 +Dox / pIND-Luci +Dox, > 2 fold down
	TNFAIP2				PRRT2		SEPN1
	TNNI3				PRSS12		SEPT7L
	TNS1				PRSS30P		SERP2
	TNS3				PRSS8		SERPINB1
	TOB2P1				PRUNE2		SERTAD2
	TOMM40				PSD3		SERTAD3
	TOP1				psiTPTE22		SERTAD4
	TOR1A				PTCD3		SFRP1
	TP53				PTH2		SFXN3
	TP53I3				PTP4A3		SGK196
	TRA2A				PTPN14		SGPL1
	TRAF4				PTPN18		SH2D3C
	TRAPPC6A				PTPRD		SH2D4A
	TRIAP1				PUSL1		SH2D6
	TRIM14				PXDN		SH3BP4
	TRIP11				Q29HP5		SH3GLB2
	TRMT1L				Q5SRJ3		SH3YL1
	TRMT61A				Q6P4E4		SHISA2
	TRPV1				QPCT		SHISA3
	TRPV2				RAB11FIP1		SHISA8
	TSPAN10				RAB11FIP5		SIGLEC15
	TSPAN12				RAB26		SIRT4
	TSR1				RAB37		SIX4
	TTC12				RAB3A		SKI
	TTC18				RAB3IP		SKIL
	TTC4				RAB4B		SKP2
	TTC8				RAB6B		SLC12A4
	TTF1				RAD23A		SLC13A3
	TTPAL				RALB		SLC15A4
	TUB				RALGDS		SLC16A10
	TUBA3D				RASA4		SLC16A5
	TUBA8				RASGEF1B		SLC22A23
	TUBGCP5				RASGRP2		SLC25A23
	TXNDC16				RASL10A		SLC25A25
	U2SURP				RASSF3		SLC25A27
	UAP1L1				RAVER1		SLC25A37
	UBAC2-AS1				RBAK-LOC389458		SLC25A43
	UBE2Q1				RBBP5		SLC27A1
	UBQLN1				RBM11		SLC33A1
	UBQLN2				RBM20		SLC35B2
	UBR5				RBMS2		SLC35F1
	UBR7				RBP1		SLC39A11
	UCKL1				RBP7		SLC46A1
	UFSP1				RBPJ		SLC46A2
	UFSP2				RBPMS		SLC4A7
	UGGT2				RCAN3		SLC5A9
	UMPS				RDM1		SLC6A6
	UNC5C				REEP2		SLC7A2
	UNG				RER1		SMC1A
	URB1				RET		SMC2
	URM1				RFTN1		SMCR6
	UTP14A				RGMA		SMTNL2
	UTP15				RGS10		SNAP91
	UTP20				RGS12		SNHG6
	VAV1				RGS16		SNORA17
	VAV3				RGS9BP		SNORA43
	VAX1				RHCE		SNORA56
	VAX2				RHOB		SNORA65
	VMAC				RHOBTB2		SNORD12B
	VPS18				RHOC		SNORD19
	VRK1				RHOQ		SNORD29
	VSIG10L				RHOU		SNORD31
	VTI1A				RIMS3		SNORD36A
	WARS				RINL		SNORD36C
	WBSCR27				RIPK4		SNORD58A
	WDR46				RN18S1		SNX1
	WDR73				RN5-8S1		SNX22
	WDR74				RND2		SNX30
	WDR78				RND3		SOCS7
	WDR81				RNF122		SOX6
	WDR85				RNF123		SP140
	WDR89				RNF182		SP5
	WIBG				RNF19A		SP8
	WNT10B				RNF19B		SPATA18
	WTAP				RNF2		SPINT2
	XLOC_000167				RNF208		SPON1
	XLOC_000175				RNF215		SQRDL
	XLOC_000190				RNF223		SREBF2
	XLOC_000822				RNF43		SRPK3
	XLOC_000883				RNPEPL1		SSBP4
	XLOC_000895				ROGDI		SSH2
	XLOC_002026				RORA		SSTR3
	XLOC_002643				RPH3AL		ST3GAL1
	XLOC_002652				RPL22		ST3GAL4
	XLOC_002921				RPL23AP32		ST6GALNAC6
	XLOC_005081				RPS6KA1		STAU2
	XLOC_005617				RRAS		STK32C
	XLOC_005764				RSC1A1		STMN3
	XLOC_006025				RSRC2		STON1-GTF2A1L
	XLOC_007052				RTN4R		STX17
	XLOC_007348				RUNX1		STXBP1
	XLOC_007433				RUNX1T1		STYX
	XLOC_007769				RUSC2		SV2A
	XLOC_008033				RXRB		SYDE2
	XLOC_009147				S100A10		SYK
	XLOC_009509				S100A11		SYN2
	XLOC_010495				SAMD10		SYNM
	XLOC_011344				SAP25		SYT15
	XLOC_011515				SAT1		TAF5L
	XLOC_013282				SATB1		TAGLN2
	XLOC_013301				SBF1		TAS1R3
	XLOC_014063				SBF1P1		TBC1D1
	XLOC_014288				SBF2		TBC1D16
	XLOC_014397				SBNO1		TBC1D3
	XLOC_l2_001971				SCAI		TBC1D5
	XLOC_l2_003602				SCAPER		TBC1D9
	XLOC_l2_004222				SCARA5		TC2N
	XLOC_l2_006718				SCARNA12		TCF24
	XLOC_l2_009273				SCARNA14		TCP11L1
	XLOC_l2_009442				SCARNA20		TCTEX1D2
	XLOC_l2_011649				SCML1		TDRD3
	XLOC_l2_013267				SCUBE1		TECPR2
	XLOC_l2_013480				SDHAP1		TFAP2A
	XLOC_l2_015451				SDHB		TFCP2L1
	XLOC_l2_015491				SEC14L2		TGFBR3
	XLOC_l2_015585				SEC24D		THAP6
	XPO5				SEL1L3		THBD
	ZBTB2				SELO		TIMP3
	ZBTB24				SEMA3F		TJP2

Supplementary Table 1

pIND-CNOT1 AND 2 AND 3 +Dox / pIND-Luci +Dox, > 2 fold up	pIND-CNOT1 +Dox / pIND-Luci +Dox, > 2 fold up	pIND-CNOT2 +Dox / pIND-Luci +Dox, > 2 fold up	pIND-CNOT3 +Dox / pIND-Luci +Dox, > 2 fold up	pIND-CNOT1 AND 2 AND 3 +Dox / pIND-Luci +Dox, > 2 fold down	pIND-CNOT1 +Dox / pIND-Luci +Dox, > 2 fold down	pIND-CNOT2 +Dox / pIND-Luci +Dox, > 2 fold down	pIND-CNOT3 +Dox / pIND-Luci +Dox, > 2 fold down
	ZBTB25				SEMA4C		TLX2
	ZC3H14				SEMA4G		TM6SF1
	ZC3H4				SENP7		TMCC1
	ZCWPW1				SEPN1		TMED4
	ZDHHC14				SEPT3		TMEM101
	ZER1				SERHL2		TMEM132E
	ZFHX2				SERP2		TMEM187
	ZFP112				SERPINB1		TMEM203
	ZFP41				SERPINF1		TMEM229B
	ZFYVE1				SERPINI1		TMEM38B
	ZFYVE26				SESTD1		TMEM52
	ZHX2				SFN		TMEM8B
	ZHX3				SFRP1		TMOD1
	ZKSCAN5				SFTA3		TMX3
	ZMIZ2				SFTPA2		TNFRSF10A
	ZNF17				SFXN3		TNFRSF10B
	ZNF174				SGK196		TNFRSF11A
	ZNF180				SGMS1		TNFRSF12A
	ZNF189				SGPP2		TNFRSF25
	ZNF23				SGSM3		TNK2
	ZNF251				SH2D3C		TOR1B
	ZNF252				SH2D4A		TP53I11
	ZNF26				SH2D6		TP73-AS1
	ZNF271				SH3BGRL3		TPD52L1
	ZNF280D				SH3GL2		TPPP
	ZNF283				SH3YL1		TRAF2
	ZNF300				SHD		TRAK1
	ZNF324				SHISA2		TRIB1
	ZNF324B				SHISA8		TRIM32
	ZNF326				SIGIRR		TRIM38
	ZNF362				SIGLEC15		TRIM61
	ZNF425				SLC10A4		TRIM7
	ZNF462				SLC16A4		TRIM8
	ZNF480				SLC16A5		TRPM4
	ZNF498				SLC22A23		TSC1
	ZNF512B				SLC25A27		TSHZ1
	ZNF551				SLC25A30		TSIX
	ZNF577				SLC25A35		TTC28-AS1
	ZNF594				SLC25A37		TTC39A
	ZNF605				SLC25A43		TUBA4A
	ZNF618				SLC27A1		TUBB2B
	ZNF667				SLC35B2		TUBBP5
	ZNF688				SLC35E2B		TUSC3
	ZNF707				SLC35F3		TWIST2
	ZNF71				SLC35F5		TXNDC17
	ZNF75A				SLC39A7		TXNL4A
	ZNF75D				SLC45A3		TYSND1
	ZNF765				SLC46A2		U2AF1L4
	ZNF773				SLC5A9		UBE2V2
	ZNF776				SLC7A2		UBE3B
	ZNF777				SLC7A5		UGCG
	ZNF837				SLIT2		UPP1
	ZNF839				SLIT3		URGCP
	ZNRF1				SMAD2		USP2
	ZSCAN5A				SMCR6		USP32
	ZSWIM1				SMTNL2		USP41
	ZSWIM3				SNAI1		USP49
	ZSWIM5				SNAPC3		USP53
					SNCG		UVRAG
					SNORA12		VAMP8
					SNORA17		VAT1
					SNORA57		VAV2
					SNORD105B		VN1R2
					SNORD11		VPS13A
					SNORD12B		VTRNA1-3
					SNORD19		VWA5B2
					SNORD26		WDYHV1
					SNORD27		WHAMMP2
					SNORD31		WNK2
					SNORD41		WNT11
					SNORD43		WNT3
					SNORD48		WNT6
					SNORD52		WT1
					SNORD56		XIAP
					SNORD58A		XLOC_000011
					SNORD76		XLOC_000152
					SNORD8		XLOC_000182
					SNORD80		XLOC_000441
					SNORD89		XLOC_000670
					SNX24		XLOC_000735
					SOCS2		XLOC_000776
					SOCS5		XLOC_001357
					SOS1		XLOC_001373
					SOX6		XLOC_001453
					SP140		XLOC_001515
					SP5		XLOC_001856
					SP8		XLOC_002063
					SP9		XLOC_002140
					SPAG4		XLOC_002283
					SPARC		XLOC_002581
					SPATA2L		XLOC_002603
					SPEN		XLOC_002746
					SPINK2		XLOC_002900
					SPINK7		XLOC_003165
					SPINT2		XLOC_003405
					SPRED2		XLOC_003870
					SPRR2D		XLOC_003912
					SREBF2		XLOC_004263
					SRPK3		XLOC_004452
					SSBP4		XLOC_004680
					SSFA2		XLOC_005051
					ST13		XLOC_005225
					ST3GAL1		XLOC_005461
					ST3GAL5		XLOC_005633
					STAMBPL1		XLOC_006336
					STARD5		XLOC_006390
					STIM2		XLOC_006419
					STK3		XLOC_006513
					STK32C		XLOC_006721
					STK40		XLOC_006753
					STON1		XLOC_006844
					STON1-GTF2A1L		XLOC_006994
					STX7		XLOC_007054
					STX8		XLOC_007275
					SUCLG2		XLOC_007467
					SV2A		XLOC_007556
					SYCE3		XLOC_007855
					SYK		XLOC_008000
					SYNDIG1		XLOC_008001

Supplementary Table 1

pIND-CNOT1 AND 2 AND 3 +Dox / pIND-Luci +Dox, > 2 fold up	pIND-CNOT1 +Dox / pIND-Luci +Dox, > 2 fold up	pIND-CNOT2 +Dox / pIND-Luci +Dox, > 2 fold up	pIND-CNOT3 +Dox / pIND-Luci +Dox, > 2 fold up	pIND-CNOT1 AND 2 AND 3 +Dox / pIND-Luci +Dox, > 2 fold down	pIND-CNOT1 +Dox / pIND-Luci +Dox, > 2 fold down	pIND-CNOT2 +Dox / pIND-Luci +Dox, > 2 fold down	pIND-CNOT3 +Dox / pIND-Luci +Dox, > 2 fold down
					SYNGR1		XLOC_008005
					SYT1		XLOC_008015
					SYT15		XLOC_008149
					TACC1		XLOC_008185
					TAF1B		XLOC_008479
					TAGLN2		XLOC_008542
					TBC1D1		XLOC_008586
					TBC1D5		XLOC_008652
					TBX19		XLOC_008935
					TCEAL6		XLOC_009006
					TCF20		XLOC_009199
					TCF24		XLOC_009451
					TDRD3		XLOC_009628
					TDRKH		XLOC_009920
					TEF		XLOC_010167
					TEKT2		XLOC_010533
					TFAP2A		XLOC_011226
					TFPI		XLOC_011248
					TGFB1I1		XLOC_011306
					TGFBR3		XLOC_011407
					THAP3		XLOC_011518
					THBD		XLOC_011765
					THBS3		XLOC_011819
					THOC5		XLOC_011950
					TIMP3		XLOC_011984
					TKTL1		XLOC_012338
					TLX2		XLOC_012503
					TM6SF1		XLOC_012505
					TMBIM1		XLOC_012670
					TMC7		XLOC_012678
					TMCO3		XLOC_012905
					TMED9		XLOC_013005
					TMEM101		XLOC_013162
					TMEM132E		XLOC_013434
					TMEM18		XLOC_013449
					TMEM191A		XLOC_013615
					TMEM191B		XLOC_013679
					TMEM219		XLOC_l2_000297
					TMEM27		XLOC_l2_000339
					TMEM52		XLOC_l2_000864
					TMEM59L		XLOC_l2_001206
					TMEM8B		XLOC_l2_001890
					TMEM91		XLOC_l2_001952
					TMSB4X		XLOC_l2_002076
					TMUB1		XLOC_l2_002469
					TNFRSF10A		XLOC_l2_003039
					TNFRSF10D		XLOC_l2_003293
					TNFRSF12A		XLOC_l2_004168
					TNFRSF21		XLOC_l2_004706
					TNFRSF25		XLOC_l2_005997
					TNK2		XLOC_l2_006013
					TNKS2		XLOC_l2_007034
					TPD52L1		XLOC_l2_007585
					TPK1		XLOC_l2_008221
					TPM2		XLOC_l2_009281
					TPPP3		XLOC_l2_009316
					TPRG1L		XLOC_l2_011145
					TPTE		XLOC_l2_012925
					TRAF3IP2-AS1		XLOC_l2_013001
					TRAK1		XLOC_l2_013131
					TRAPPC3		XLOC_l2_013458
					TRIM38		XLOC_l2_013845
					TRIM58		XLOC_l2_014098
					TRIM66		XLOC_l2_014505
					TRIM69		XLOC_l2_014549
					TRMT11		XLOC_l2_014602
					TSC22D3		XLOC_l2_014697
					TSIX		XLOC_l2_015148
					TSPAN18		XLOC_l2_015196
					TSPO		XLOC_l2_015351
					TSPYL2		XLOC_l2_015641
					TTC21A		XPNPEP3
					TTC28-AS1		XRRA1
					TTC32		YBX2
					TTLL1		YPEL1
					TUBB2B		ZADH2
					TUBB4A		ZBED6
					TUBD1		ZBTB34
					TUSC3		ZBTB4
					TWIST2		ZBTB42
					TXNL4A		ZBTB43
					UBA2		ZBTB44
					UBA6		ZBTB47
					UBE2E1		ZBTB6
					UBE2R2		ZBTB7B
					UBXN7		ZCCHC24
					ULBP1		ZCCHC6
					UNC5B		ZDHHC12
					UPP1		ZDHHC2
					USP2		ZDHHC21
					USP31		ZDHHC23
					USP32		ZFP161
					USP41		ZFP37
					USP48		ZFYVE9
					USP49		ZIC5
					UTF1		ZMAT3
					UTS2R		ZNF100
					UVRAG		ZNF121
					VAMP8		ZNF136
					VANGL2		ZNF14
					VAT1		ZNF141
					VLDLR		ZNF157
					VN1R2		ZNF160
					VRK2		ZNF169
					VWA5B2		ZNF222
					VWDE		ZNF226
					WASF2		ZNF230
					WDFY1		ZNF234
					WDSUB1		ZNF248
					WDYHV1		ZNF25
					WIPF3		ZNF253
					WIPI1		ZNF254
					WNT6		ZNF256
					WRAP73		ZNF257
					WTIP		ZNF264
					XIAP		ZNF28
					XLOC_000011		ZNF284
					XLOC_000048		ZNF295
					XLOC_000101		ZNF333

Supplementary Table 1

pIND-CNOT1 AND 2 AND 3 +Dox / pIND-Luci +Dox, > 2 fold up	pIND-CNOT1 +Dox / pIND-Luci +Dox, > 2 fold up	pIND-CNOT2 +Dox / pIND-Luci +Dox, > 2 fold up	pIND-CNOT3 +Dox / pIND-Luci +Dox, > 2 fold up	pIND-CNOT1 AND 2 AND 3 +Dox / pIND-Luci +Dox, > 2 fold down	pIND-CNOT1 +Dox / pIND-Luci +Dox, > 2 fold down	pIND-CNOT2 +Dox / pIND-Luci +Dox, > 2 fold down	pIND-CNOT3 +Dox / pIND-Luci +Dox, > 2 fold down
					XLOC_000182		ZNF345
					XLOC_000527		ZNF382
					XLOC_000670		ZNF426
					XLOC_000743		ZNF429
					XLOC_000776		ZNF433
					XLOC_001097		ZNF441
					XLOC_001228		ZNF451
					XLOC_001257		ZNF468
					XLOC_001320		ZNF484
					XLOC_001357		ZNF493
					XLOC_001373		ZNF497
					XLOC_001453		ZNF516
					XLOC_001515		ZNF519
					XLOC_001609		ZNF524
					XLOC_001699		ZNF532
					XLOC_001856		ZNF555
					XLOC_002063		ZNF564
					XLOC_002211		ZNF592
					XLOC_002283		ZNF600
					XLOC_002581		ZNF621
					XLOC_002603		ZNF625
					XLOC_002616		ZNF652
					XLOC_002746		ZNF653
					XLOC_002900		ZNF672
					XLOC_003165		ZNF684
					XLOC_003870		ZNF703
					XLOC_003912		ZNF704
					XLOC_004452		ZNF709
					XLOC_004680		ZNF710
					XLOC_005051		ZNF714
					XLOC_005225		ZNF727
					XLOC_005633		ZNF738
					XLOC_005737		ZNF780A
					XLOC_006144		ZNF782
					XLOC_006419		ZNF79
					XLOC_006844		ZNF792
					XLOC_006994		ZNF823
					XLOC_007054		ZNF880
					XLOC_007222		ZNF98
					XLOC_007275		ZRANB1
					XLOC_007855		ZSCAN18
					XLOC_008001		ZSCAN2
					XLOC_008005		ZXDB
					XLOC_008015		
					XLOC_008149		
					XLOC_008185		
					XLOC_008542		
					XLOC_008586		
					XLOC_008652		
					XLOC_008935		
					XLOC_008982		
					XLOC_009114		
					XLOC_009181		
					XLOC_009199		
					XLOC_009451		
					XLOC_009628		
					XLOC_010112		
					XLOC_010167		
					XLOC_010275		
					XLOC_010377		
					XLOC_011248		
					XLOC_011305		
					XLOC_011306		
					XLOC_011373		
					XLOC_011407		
					XLOC_011645		
					XLOC_011682		
					XLOC_011819		
					XLOC_011950		
					XLOC_011984		
					XLOC_012338		
					XLOC_012503		
					XLOC_012505		
					XLOC_012670		
					XLOC_012678		
					XLOC_012848		
					XLOC_012905		
					XLOC_012991		
					XLOC_013005		
					XLOC_013108		
					XLOC_013162		
					XLOC_013434		
					XLOC_013449		
					XLOC_013615		
					XLOC_013679		
					XLOC_013981		
					XLOC_013985		
					XLOC_l2_000010		
					XLOC_l2_000339		
					XLOC_l2_000636		
					XLOC_l2_000791		
					XLOC_l2_000864		
					XLOC_l2_001592		
					XLOC_l2_001890		
					XLOC_l2_002469		
					XLOC_l2_003039		
					XLOC_l2_003293		
					XLOC_l2_004168		
					XLOC_l2_004706		
					XLOC_l2_004854		
					XLOC_l2_005020		
					XLOC_l2_005793		
					XLOC_l2_005997		
					XLOC_l2_007585		
					XLOC_l2_009281		
					XLOC_l2_009316		
					XLOC_l2_011043		
					XLOC_l2_011145		
					XLOC_l2_011415		
					XLOC_l2_011987		
					XLOC_l2_012023		
					XLOC_l2_012902		
					XLOC_l2_013131		
					XLOC_l2_014098		
					XLOC_l2_014602		
					XLOC_l2_014697		
					XLOC_l2_015178		
					XLOC_l2_015201		
					XLOC_l2_015562		

Supplementary Table 1

pIND-CNOT1 AND 2 AND 3 +Dox / pIND-Luci +Dox, > 2 fold up	pIND-CNOT1 +Dox / pIND-Luci +Dox, > 2 fold up	pIND-CNOT2 +Dox / pIND-Luci +Dox, > 2 fold up	pIND-CNOT3 +Dox / pIND-Luci +Dox, > 2 fold up	pIND-CNOT1 AND 2 AND 3 +Dox / pIND-Luci +Dox, > 2 fold down	pIND-CNOT1 +Dox / pIND-Luci +Dox, > 2 fold down	pIND-CNOT2 +Dox / pIND-Luci +Dox, > 2 fold down	pIND-CNOT3 +Dox / pIND-Luci +Dox, > 2 fold down
					XLOC_l2_015641		
					XPNPEP3		
					XRRA1		
					YBX2		
					YIPF4		
					YPEL1		
					YPEL2		
					ZBTB16		
					ZBTB17		
					ZBTB5		
					ZC3H12B		
					ZC3H6		
					ZDHHC2		
					ZDHHC21		
					ZDHHC23		
					ZFAND2B		
					ZKSCAN4		
					ZMAT3		
					ZMYM2		
					ZMYM6		
					ZMYND11		
					ZNF141		
					ZNF157		
					ZNF182		
					ZNF193		
					ZNF205		
					ZNF224		
					ZNF226		
					ZNF449		
					ZNF460		
					ZNF513		
					ZNF516		
					ZNF524		
					ZNF642		
					ZNF643		
					ZNF692		
					ZNF703		
					ZNF774		
					ZNF862		
					ZRANB1		
					ZRANB3		

Supplementary Table 2

**KEGG pathways**	**MHC II genes are in bold, Log$_2$ fold change in CNOT2 knock-down: -2.0/-1.5/-1.0/-0.5/0/0.5/1.0/1.5/2.0**
**KEGG:4940** Type I diabetes mellitus	CD28, CD80, CD86, CPE, FAS, FASLG, GAD1, GAD2, GZMB, HLA-A, HLA-B, HLA-C, **HLA-DMA, HLA-DMB, HLA-DOA, HLA-DOB, HLA-DPA1, HLA-DPB1, HLA-DQA1, HLA-DQA2, HLA-DQB1, HLA-DRA, HLA-DRB1, HLA-DRB3, HLA-DRB4, HLA-DRB5,** HLA-E, HLA-F, HLA-G, HSPD1, ICA1, IFNG, IL12A, IL12B, IL1A, IL1B, IL2, INS, LOC105369230, LTA, PRF1, PTPRN, PTPRN2, TNF
**KEGG:4145** Phagosome	(Hs.387679), (Hs.511743), ACTB, ACTG1, ATP6AP1, ATP6V0A1, ATP6V0A2, ATP6V0A4, ATP6V0B, ATP6V0C, ATP6V0D1, ATP6V0D2, ATP6V0E1, ATP6V0E2, ATP6V1A, ATP6V1B1, ATP6V1B2, ATP6V1C1, ATP6V1C2, ATP6V1D, ATP6V1E1, ATP6V1E2, ATP6V1F, ATP6V1G1, ATP6V1G2, ATP6V1G3, ATP6V1H, C1R, C3, CALR, CANX, CD14, CD209, CD36, CLEC4M, CLEC7A, COLEC11, COLEC12, COMP, CORO1A, CTSL, CTSS, CYBA, CYBB, DYNC1H1, DYNC1I1, DYNC1I2, DYNC1LI1, DYNC1LI2, DYNC2H1, EEA1, FCAR, FCGR1A, FCGR2A, FCGR2B, FCGR2C, FCGR3A, FCGR3B, HGS, HLA-A, HLA-B, HLA-C, **HLA-DMA, HLA-DMB, HLA-DOA, HLA-DOB, HLA-DPA1, HLA-DPB1, HLA-DQA1, HLA-DQA2, HLA-DQB1, HLA-DRA, HLA-DRB1, HLA-DRB3, HLA-DRB4, HLA-DRB5,** HLA-E, HLA-F, HLA-G, ITGA2, ITGA5, ITGAM, ITGAV, ITGB1, ITGB2, ITGB3, ITGB5, LAMP1, LAMP2, LOC102723407, LOC105369230, M6PR, MARCO, MBL2, MPO, MRC1, MRC2, MSR1, NCF1, NCF2, NCF4, NOS1, NOX1, NOX3, OLR1, PIK3C3, PIKFYVE, PLA2R1, RAB5A, RAB5B, RAB5C, RAB7A, RAB7B, RAC1, RILP, SCARB1, SEC22B, SEC61A1, SEC61A2, SEC61B, SEC61G, SFTPA1, SFTPA2, SFTPD, STX12, STX18, STX7, TAP1, TAP2, TCIRG1, TFRC, THBS1, THBS2, THBS3, THBS4, TLR2, TLR4, TLR6, TUBA1A, TUBA1B, TUBA1C, TUBA3C, TUBA3D, TUBA3E, TUBA4A, TUBA8, TUBAL3, TUBB, TUBB1, TUBB2A, TUBB2B, TUBB3, TUBB4A, TUBB4B, TUBB6, TUBB8, VAMP3
**KEGG:5330** Allograft rejection	CD28, CD40, CD40LG, CD80, CD86, FAS, FASLG, GZMB, HLA-A, HLA-B, HLA-C, **HLA-DMA, HLA-DMB, HLA-DOA, HLA-DOB, HLA-DPA1, HLA-DPB1, HLA-DQA1, HLA-DQA2, HLA-DQB1, HLA-DRA, HLA-DRB1, HLA-DRB3, HLA-DRB4, HLA-DRB5,** HLA-E, HLA-F, HLA-G, IFNG, IL10, IL12A, IL12B, IL2, IL4, IL5, LOC102723407, LOC105369230, PRF1, TNF
**KEGG:5310** Asthma	CCL11, CD40, CD40LG, EPX, FCER1A, FCER1G, **HLA-DMA, HLA-DMB, HLA-DOA, HLA-DOB, HLA-DPA1, HLA-DPB1, HLA-DQA1, HLA-DQA2, HLA-DQB1, HLA-DRA, HLA-DRB1, HLA-DRB3, HLA-DRB4, HLA-DRB5,** IL10, IL13, IL3, IL4, IL5, IL9, LOC102723407, LOC105369230, MS4A2, PRG2, RNASE3, TNF
**KEGG:05332** Graft-versus-host disease	CD28, CD80, CD86, FAS, FASLG, GZMB, HLA-A, HLA-B, HLA-C, **HLA-DMA, HLA-DMB, HLA-DOA, HLA-DOB, HLA-DPA1, HLA-DPB1, HLA-DQA1, HLA-DQA2, HLA-DQB1, HLA-DRA, HLA-DRB1, HLA-DRB3, HLA-DRB4, HLA-DRB5,** HLA-E, HLA-F, HLA-G, IFNG, IL1A, IL1B, IL2, IL6, KIR2DL1, KIR2DL2, KIR2DL3, KIR2DL5A, KIR3DL1, KIR3DL2, KLRC1, KLRD1, LOC105369230, PRF1, TNF
**KEGG:05140** Leishmaniasis	C3, CR1, CYBA, ELK1, FCGR1A, FCGR2A, FCGR2C, FCGR3A, FCGR3B, FOS, **HLA-DMA, HLA-DMB, HLA-DOA, HLA-DOB, HLA-DPA1, HLA-DPB1, HLA-DQA1, HLA-DQA2, HLA-DQB1, HLA-DRA, HLA-DRB1, HLA-DRB3, HLA-DRB4, HLA-DRB5,** IFNG, IFNGR1, IFNGR2, IL10, IL12A, IL12B, IL1A, IL1B, IL4, IRAK1, IRAK4, ITGA4, ITGAM, ITGB1, ITGB2, JAK1, JAK2, JUN, LOC102723407, LOC105369230, MAP3K7, MAPK1, MAPK11, MAPK12, MAPK13, MAPK14, MAPK3, MARCKSL1, MYD88, NCF1, NCF2, NCF4, NFKB1, NFKBIA, NFKBIB, NOS2, PRKCB, PTGS2, PTPN6, RELA, STAT1, TAB1, TAB2, TGFB1, TGFB2, TGFB3, TLR2, TLR4, TNF, TRAF6
**KEGG:04612** Antigen processing and presentation	B2M, CALR, CANX, CD4, CD74, CD8A, CD8B, CIITA, CREB1, CTSB, CTSL, CTSS, HLA-A, HLA-B, HLA-C, **HLA-DMA, HLA-DMB, HLA-DOA, HLA-DOB, HLA-DPA1, HLA-DPB1, HLA-DQA1, HLA-DQA2, HLA-DQB1, HLA-DRA, HLA-DRB1, HLA-DRB3, HLA-DRB4, HLA-DRB5,** HLA-E, HLA-F, HLA-G, HSP90AA1, HSP90AB1, HSPA1A, HSPA1B, HSPA1L, HSPA2, HSPA4, HSPA5, HSPA6, HSPA8, IFI30, IFNG, KIR2DL1, KIR2DL2, KIR2DL3, KIR2DL4, KIR2DL5A, KIR2DS1, KIR2DS2, KIR2DS3, KIR2DS4, KIR2DS5, KIR3DL1, KIR3DL2, KIR3DL3, KLRC1, KLRC2, KLRC3, KLRC4, KLRD1, LGMN, LOC105369230, NFYA, NFYB, NFYC, PDIA3, PSME1, PSME2, PSME3, RFX5, RFXANK, RFXAP, TAP1, TAP2, TAPBP, TNF
**KEGG:05145** Toxoplasmosis	AKT1, AKT2, AKT3, ALOX5, BAD, BCL2, BCL2L1, BIRC2, BIRC3, BIRC7, BIRC8, CASP3, CASP8, CASP9, CCR5, CD40, CD40LG, CHUK, CIITA, CYCS, GNAI1, GNAI2, GNAI3, GNAO1, **HLA-DMA, HLA-DMB, HLA-DOA, HLA-DOB, HLA-DPA1, HLA-DPB1, HLA-DQA1, HLA-DQA2, HLA-DQB1, HLA-DRA, HLA-DRB1, HLA-DRB3, HLA-DRB4, HLA-DRB5,** HSPA1A, HSPA1B, HSPA1L, HSPA2, HSPA6, HSPA8, IFNG, IFNGR1, IFNGR2, IKBKB, IKBKG, IL10, IL10RA, IL10RB, IL12A, IL12B, IRAK1, IRAK4, IRGM, ITGA6, ITGB1, JAK1, JAK2, LAMA1, LAMA2, LAMA3, LAMA4, LAMA5, LAMB1, LAMB2, LAMB3, LAMB4, LAMC1, LAMC2, LAMC3, LDLR, LOC105369230, LY96, MAP2K3, MAP2K6, MAP3K7, MAPK1, MAPK10, MAPK11, MAPK12, MAPK13, MAPK14, MAPK3, MAPK8, MAPK9, MYD88, NFKB1, NFKBIA, NFKBIB, NOS2, PDPK1, PIK3CA, PIK3CB, PIK3CD, PIK3CG, PIK3R1, PIK3R2, PIK3R3, PIK3R5, PPIF, RELA, SOCS1, STAT1, STAT3, TAB1, TAB2, TGFB1, TGFB2, TGFB3, TLR2, TLR4, TNF, TNFRSF1A, TRAF6, TYK2, XIAP
**KEGG:05150** Staphylococcus aureus infection	C1QA, C1QB, C1QC, C1R, C1S, C2, C3, C3AR1, C4A, C4B, C5, C5AR1, CFB, CFD, CFH, CFI, DSG1, FCAR, FCGR1A, FCGR2A, FCGR2B, FCGR2C, FCGR3A, FCGR3B, FGG, FPR1, FPR2, FPR3, **HLA-DMA, HLA-DMB, HLA-DOA, HLA-DOB, HLA-DPA1, HLA-DPB1, HLA-DQA1, HLA-DQA2, HLA-DQB1, HLA-DRA, HLA-DRB1, HLA-DRB3, HLA-DRB4, HLA-DRB5,** ICAM1, IL10, ITGAL, ITGAM, ITGB2, KRT10, LOC102723407, LOC105369230, MASP1, MASP2, MBL2, PLG, PTAFR, SELP, SELPLG
**KEGG:04672** Intestinal immune network for IgA production	AICDA, CCL25, CCL28, CCR10, CCR9, CD28, CD40, CD40LG, CD80, CD86, CXCL12, CXCR4, **HLA-DMA, HLA-DMB, HLA-DOA, HLA-DOB, HLA-DPA1, HLA-DPB1, HLA-DQA1, HLA-DQA2, HLA-DQB1, HLA-DRA, HLA-DRB1, HLA-DRB3, HLA-DRB4, HLA-DRB5,** ICOS, ICOSLG, IL10, IL15, IL15RA, IL2, IL4, IL5, IL6, ITGA4, ITGB7, LOC102723407, LOC102723996, LOC105369230, LTBR, MADCAM1, MAP3K14, PIGR, TGFB1, TNFRSF13B, TNFRSF13C, TNFRSF17, TNFSF13, TNFSF13B

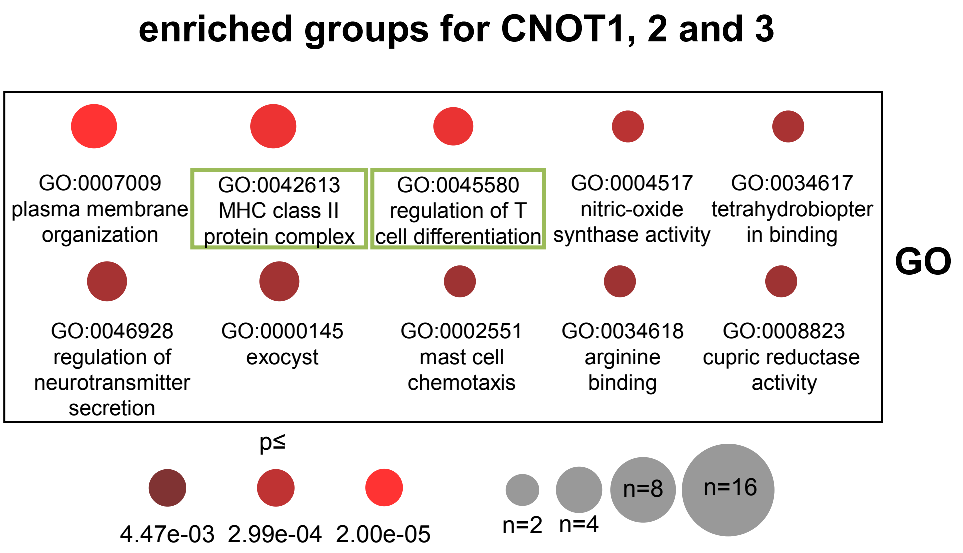

Supplementary Fig. 2. GO groups enriched after knock-down of CNOT1, CNOT2 or CNOT3. All CNOT-dependent genes regulated in either direction >2-fold were subjected to GO analysis of regulated gene sets as determined by a Fisher's exact test for overrepresented groups. The names of the groups containing MHC class II genes are highlighted by a green box.

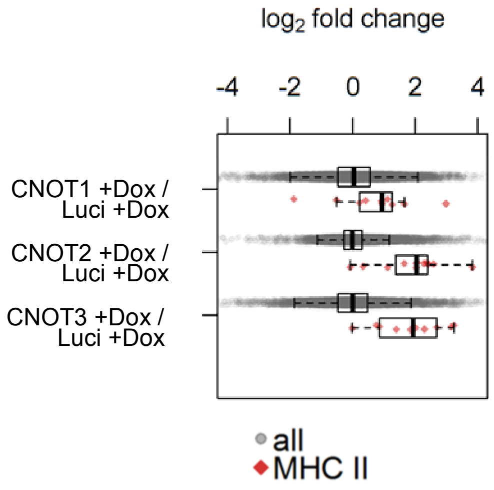

Supplementary Fig. 3. MHC II genes are upregulated upon knock-down of CNOT2 and 3, and to a lesser extend of CNOT1. Box plots showing the distribution of the fold change upon CNOT1, 2 and 3 (pIND CNOT +Dox) compared to the pIND Luci control of all genes in the array (grey dots) or MHC II genes (red dots).

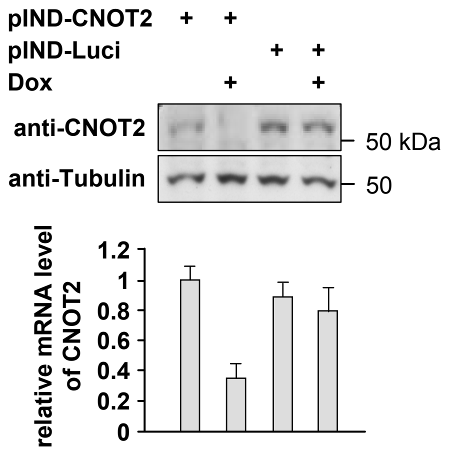

Supplementary Fig. 4. Efficient CNOT2 knock-down in the samples used for microarrays. HEK-293T cells with a stably integrated pIND CNOT2 or a pIND Luci were treated for 4 days with Dox. One aliquot of the cells was used for RNA extraction and DNA microarray analysis, while the remaining cells were lysed and tested for CNOT2 protein levels by immunoblotting as shown (upper). The lower part shows the relative expression of CNOT2 mRNA as revealed by the DNA microarray.

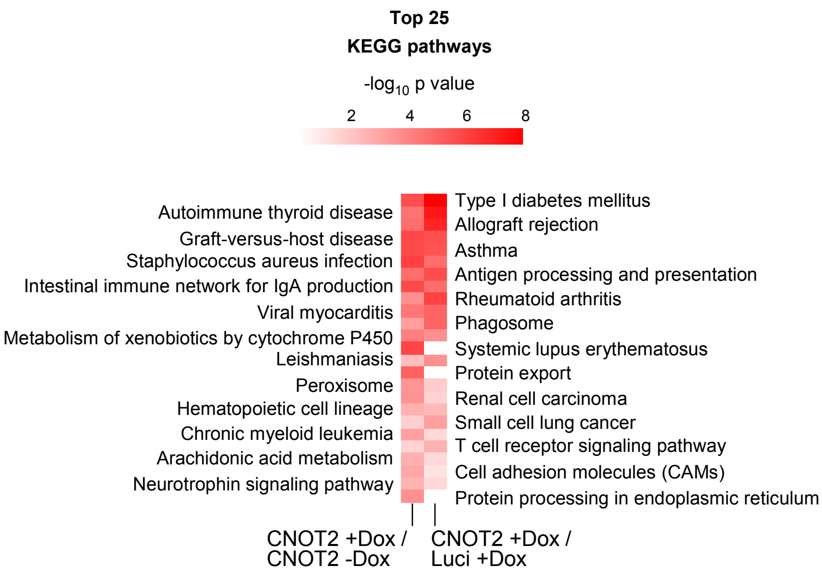

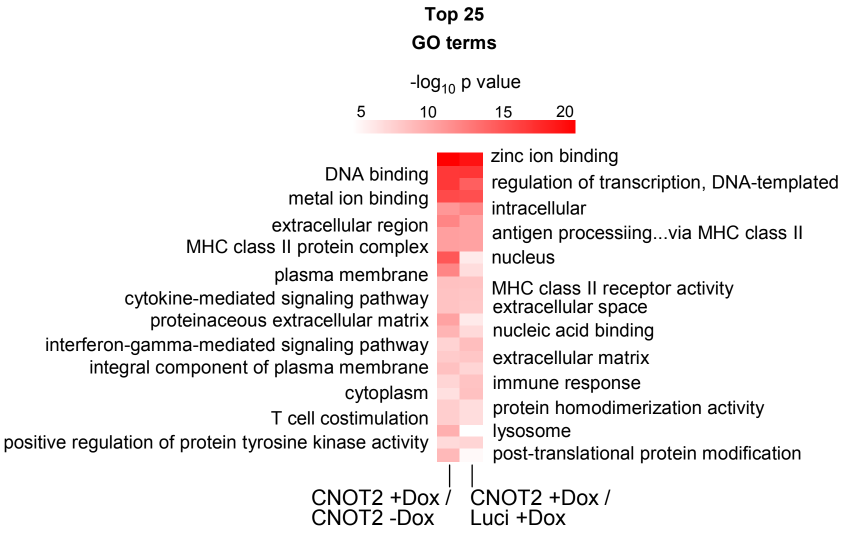

Supplementary Fig. 5. Gene set test analysis of the CNOT2 knock-down microarray data. The microarray data were evaluated by a moderate t test for significance of the changes and the data were ranked according to the t statistics. The gene set test algorithm from the Limma package was used to identify significantly enriched groups using the KEEG and GO databases. Heat maps show the 25 most significantly enriched KEGG pathways and GO terms according to the p-value obtained.

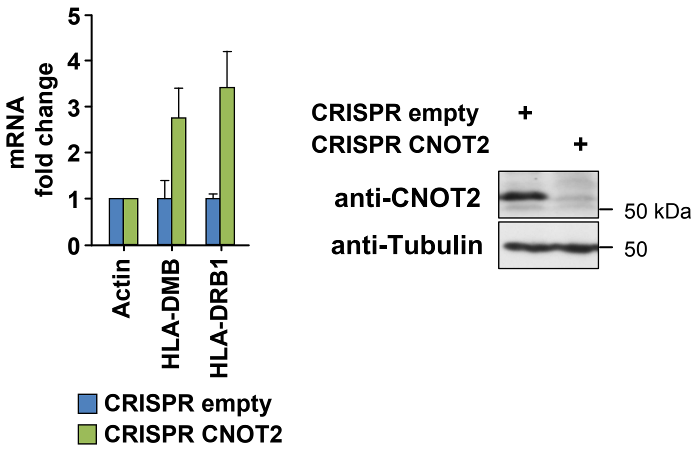

Supplementary Fig. 6. MHC II genes are upregulated upon CRISPR-Cas9 mediated deletion of CNOT2. The previously described cells with a CNOT2 indel mutation (68) were analyzed for expression of MHC II genes by RT-qPCR (left) or by Western blotting for absent expression of the CNOT2 protein. The error bars represent the standard deviation of three biological replicates.

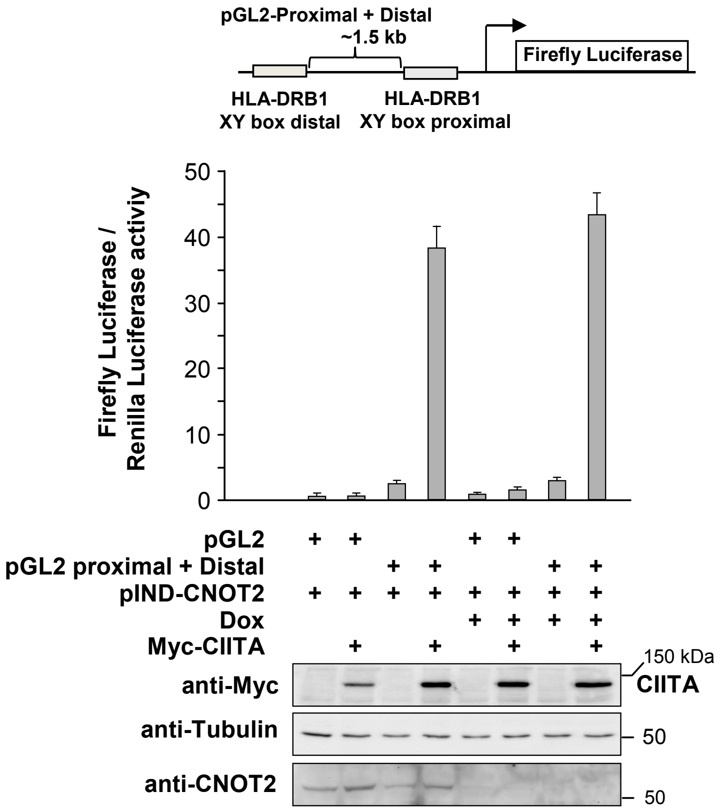

Supplementary Fig. 7. CIITA-driven gene expression is not affected by CNOT2 knock-down. HEK-293T cells with a stably integrated pIND CNOT2 were treated with Dox for 3 days or left untreated. They were then transfected with a *Renilla* luciferase and a firefly luciferase reporter construct encompassing the proximal and distal XY boxes from the HLA-DRB1 promoter or the pGL2 firefly luciferase control gene along with an expression vector for CIITA. Cells were lysed after 1 day and either tested for expression of firefly and *Renilla* luciferase activity (upper) or by Western blotting for CNOT2 knock-down and CIITA expression (lower). Firefly luciferase activity was normalized to the *Renilla* luciferase control, error bars represent the standard deviation of three biological replicates.

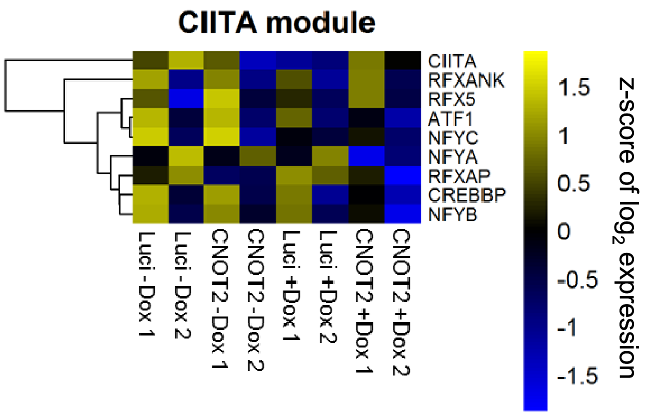

Supplementary Fig. 8. Transcription factors regulating MHC II expression (termed the CIITA module) are not upregulated upon CNOT2 knock-down. The microarray data were analysed to visualize changes in the expression of transcription factors constituting the CIITA transcriptional module. A heat map depicting relative expression changes in the different samples is shown.

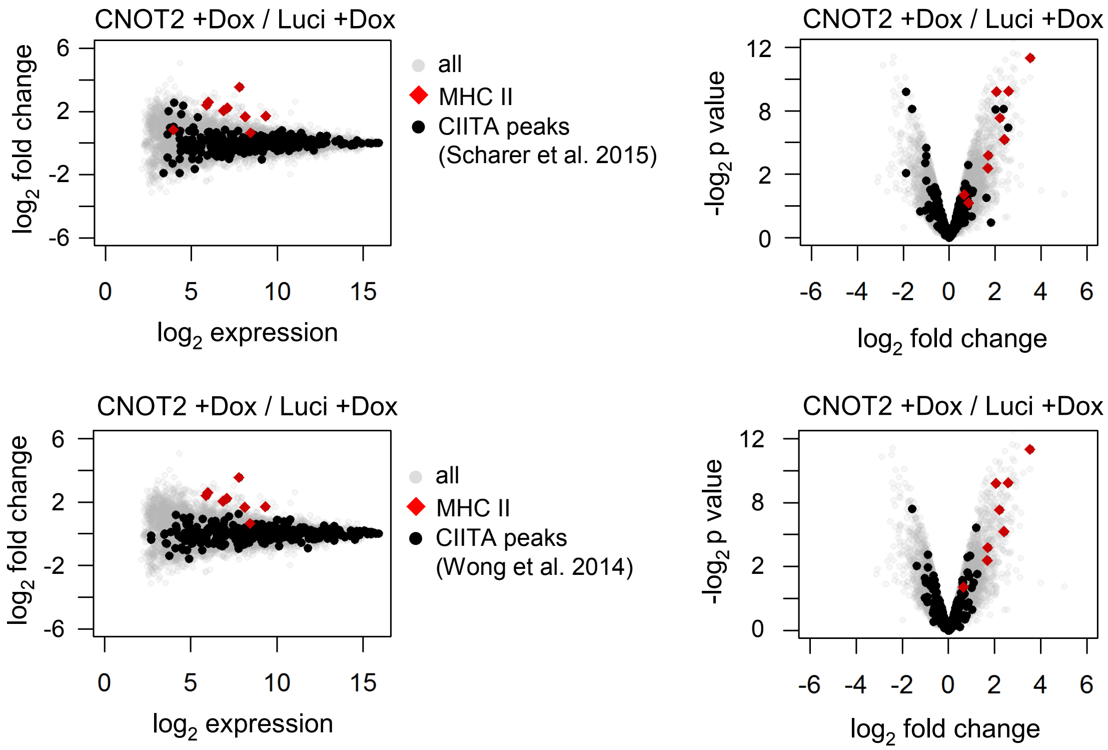

Supplementary Fig. 9. The upper panels show an MA plot (left) and Volcano plot (right) of the mRNA level changes after CNOT2 knock-down for CIITA bound genes (as revealed in a ChIP-Seq study: Scharer et al. (2015) PMID: 25753668) indicated in black. MHC-II genes are highlighted in red. The lower panels also show the MA plot (left) and Volcano plot (right) after CNOT2 knock-down for CIITA bound genes, but the genomic CIITA binding sites were determined in a separate ChIP-Seq study (Wong et al. (2014) PMID:25366989).

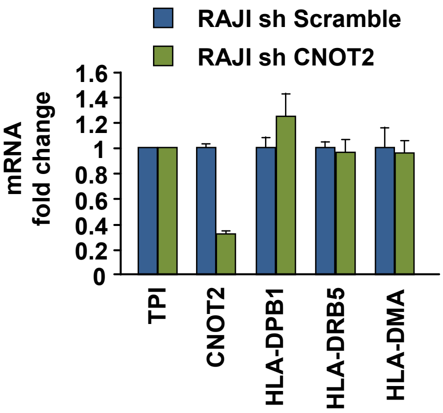

Supplementary Fig. 10. CNOT2 knock-down does not affect the expression of MHC II in CIITA positive Raji B cells. Raji B cell lymphoma cells were transfected with shRNA constructs against CNOT2 or an unspecific control. Cells were treated for two days with puromycin to eliminate untransfected cells and RT-qPCR was performed to determine the CNOT2 knock-down efficiency and expression of selected MHC II genes. Gene expression was normalized to TPI levels, error bars represent the standard deviation of three biological replicates.

Supplementary Table 3. Plasmids

Plasmid Name	Reference	Notes
pX459 (pSpCas9(BB)-2A-Puro)	1	
pCRISPR-CNOT2-3	3	sgRNA against CNOT2 cloned into pX459
pINDUCER10	2	
pINDUCER10-miR Luci	this study	shRNA against Firefly Luciferase cloned into pINDUCER10 using a PCR as described in (2), with the oligo miR-Luci-template as template
pINDUCER10-miR CNOT1	this study	shRNA against CNOT1 cloned into pINDUCER10 using a PCR as described in (2), with the oligo miR-CNOT1-template as template
pINDUCER10-miR CNOT2	this study	shRNA against CNOT2 cloned into pINDUCER10 using a PCR as described in (2), with the oligo miR-CNOT2-template as template
pINDUCER10-miR CNOT3	this study	shRNA against CNOT3 cloned into pINDUCER10 using a PCR as described in (2), with the oligo miR-CNOT3-template as template
pSUPER-Puro shScr	3	
pSUPER-Puro shCNOT2	3	
pmyc-CIITA	4	
pGFP-CNOT2	5	
pCI neo Renilla-Luci	6	
pGL2-Basic	PROMEGA	
pHD-Kan		for subcloning
pGL2-XYbox proximal	this study	A PCR of genomic DNA using the primers BglII-XYbox-DRB1-prox-F and HindIII-XYbox-DRB1-prox-R was cloned in the SmaI site of pHD-KAN, and from there, subcloned with BglII into the BglII site of pGL2-Basic, including a 1.5 kb fragment of pHD-Kan upstream of the XY box.
pGL2-XYbox distal	this study	A PCR of genomic DNA using the primers MluI-XYbox-DRB1-distal-F and XhoI-XYbox-DRB1-distal-R was cloned in the SmaI site of pHD-KAN, and from there, subcloned with MluI and XhoI into pGL2-Basic
pGL2-XYbox proximal + distal	this study	A PCR of genomic DNA using the primers MluI-XYbox-DRB1-distal-F and XhoI-XYbox-DRB1-distal-R was cloned in the SmaI site of pHD-KAN, and from there, subcloned with MluI and XhoI into pGL2-XYbox proximal.
pGL2-XYbox	this study	The plasmid pGL2-XYbox proximal + distalkbp was digested with BglII and religated, deleting a 1.2 KB fragment.
pGL2—XYbox-5X-Gal4-UAS	this study	The oligos 5xGal4-UAS-F and 5xGal4-UAS-R were annealed and cloned into the plasmid pGL2-XYbox proximal + distal 400 bp digested with XhoI and BglII.

References

1. Ran,F.A., Hsu,P.D., Wright,J., Agarwala,V., Scott,D. a and Zhang,F. (2013) Genome engineering using the CRISPR-Cas9 system. *Nat. Protoc.* , **8**, 2281–308.

2. Meerbrey,K.L., Hu,G., Kessler,J.D., Roarty,K., Li,M.Z., Fang,J.E., Herschkowitz,J.I., Burrows,A.E., Ciccia,A., Sun,T., *et al.* (2011) The pINDUCER lentiviral toolkit for inducible RNA interference in vitro and in vivo. *Proc. Natl. Acad. Sci. U. S. A.* , **108**, 3665–70.
3. Rodriguez-Gil,A., Ritter,O., Hornung,J., Stekman,H., Krüger,M., Braun,T., Kremmer,E., Kracht,M. and Schmitz,M.L. (2016) HIPK family kinases bind and regulate the function of the CCR4-NOT complex. *Mol. Biol. Cell* , **27**, 1969–80.

4. Kanazawa,S., Okamoto,T. and Peterlin,B.M. (2000) Tat competes with CIITA for the binding to P-TEFb and blocks the expression of MHC class II genes in HIV infection. *Immunity* , **12**, 61–70.
5. Albert,T.K., Lemaire,M., van Berkum,N.L., Gentz,R., Collart,M. and Timmers,H.T. (2000) Isolation and characterization of human orthologs of yeast CCR4-NOT complex subunits. *Nucleic Acids Res* , **28**, 809–817.
6. Pillai,R.S., Bhattacharyya,S.N., Artus,C.G., Zoller,T., Cougot,N., Basyuk,E., Bertrand,E. and Filipowicz,W. (2005) Inhibition of translational initiation by Let-7 MicroRNA in human cells. Science, 309, 1573–6.

Table 3: Primers

qPCR Primers	Sequence 5´to 3´
Actin-qPCR-f	TCCCTGGAGAAGAGCTACGA
Actin-qPCR-r	AGGAAGGAAGGCTGGAAGAG
APOE-qPCR-F	GACTGGCCAATCACAGGCA
APOE-qPCR-R	CGCAGGTAATCCCAAAAGCG
C1orf56-qPCR-F	TGGAAACGGGTCAGGATTGG
C1orf56-qPCR-R	GAAGTGGCCATTACCTCTGGT
CIITA-qPCR-F	CATCCTTGGGGAAGCTGAGG
CIITA-qPCR-R	AGGTAGCCACCTTCTAGGGG
CNOT2-qPCR-F	TTGGAATGATTGGCCTGTTA
CNOT2-qPCR-R	CGCAAATTTGGGGTAGAGAT
DUSP5-qPCR-F	CTGAATGTCTCCCGACGGAC
DUSP5-qPCR-R	CAGGACCTTGCCTCCCTTTT
HIST1H4E-qPCR-F	ACAGGGACGCACTCTTTACG
HIST1H4E-qPCR-R	TGGGAAGTCGAGATGCTGAG
ITGA8-qPCR-F	CTGCTGGGGAGTTTACTGGG
ITGA8-qPCR-R	ATGCCATCTGTTCTCCCGTG
RANBP6-qPCR-F	ATCCAAGCTGTATGGTGCGG
RANBP6-qPCR-R	GACAAAAGCCGTCGTAGCAG
RASSF1-qPCR-F	GCGGGTACGGCTATGGG
RASSF1-qPCR-R	AAGGTCAGGTGTCTCCCACT
RNU4ATAC-qPCR-F	TTCTTGGGGTTGCGCTACTG
RNU4ATAC-qPCR-R	AGCTCTAGTTGATGCGGGTG
RRAGB-qPCR-F	AAATCTGGGGCCGAGAATGG
RRAGB-qPCR-R	CGACGTGTGTCTCTGGCAAT
SNORA27-qPCR-F	TGGTCATTCAAGTGGGGCAA
SNORA27-qPCR-R	ACAGGATACAGACAAACACCCA
TEX19-qPCR-F	GGTGCCCACATGAACAGAGA
TEX19-qPCR-R	GGATGAAGGGGACAAGGAGC
TPI-qPCR-F	GGACTCGGAGTAATCGCCTG
TPI-qPCR-R	TGTTGGGGTGTTGCAGTCTT
mouseH2-Aa-F	CCTCTGTGGAGGTGAAGACG
mouseH2-Aa-R	TGTGTACTGGCCAATGTCTCC
mouseH2-Ab1-F	TTTGCTTTCTGAAGGGGGCA
mouseH2-Ab1-R	TCGCCCATGAACTGGTACAC
mouseH2-Dma-F	AGAAGTCAGGAGCTGTGCTG
mouseH2-Dma-R	TCCCGTCCTGGCAGAATAGA
mouseH2-DMb2-F	GTCTACACCTGCGTGGTTCA
mouseH2-DMb2-R	GGATCCGGGGAGAGGAGTG
mouseH2-Eb1-F	ACGGTGTGCAGACACAACTA
mouseH2-Eb1-R	GGCTGTGTCTTTGTGGGGTA
mouseH2-Eb2-F	CTGATGATGCTGACCCCTCC
mouseH2-Eb2-R	CAGCCTTCAACTGCTCCAGA
mouseH2-Oa-F	TGAGTCCCCGAGGAGTAAGG
mouseH2-Oa-R	GAGTGGGCGAAGTCTCCAAA
mouseH2-Ob-F	GGCTCTGTTGGTGAACCTCA
mouseH2-Ob-R	TGGTGAAGTAACAGTCCGCC
mouseActin-F	GTCCACACCCGCCACCAGTTCG
mouseActin-R	GGAATACAGCCCGGGGAGCATCGTC
mouseTPI-F	TGGTCCTGGCCTATGAACCT
mouseTPI-R	TTTCAGCCATCCCCGGA

ChIP/FAIRE Primers	
ACTB-ChIP-TSS-F-1	AAAGGCAACTTTCGGAACGG
ACTB-ChIP-TSS-R-1	TTCCTCAATCTCGCTCTCGC
ChIP neg control-2-F	TAGGACAGGCAGATGGGTCA
ChIP neg control-2-R	GCTGGCTCCTGGATTGTTCA
ChIP neg control 1 F	ATGGTTGCCACTGGGGATCT
ChIP neg control 1 R	TGCCAAAGCCTAGGGGAAGA
HLA-DMA-3'-F	TGGGGACCTAGTTAGGGAGC
HLA-DMA-3'-R	AGATCCATGGGAGGAGGCTT
HLA-DMA-5'-F	GGAGAGAACAATCTCCGCTTCA
HLA-DMA-5'-R	AGCTGCTATGTGTGGTTGGT
HLA-DMA-XYbox-F	CATCAGTCACTGGGGAGACG
HLA-DMA-XYbox-R	GCTTCCCAGCCCAGTTACAT
HLA-DPB1-3'-F	CCAGCCTAGGGTGAATGTTT
HLA-DPB1-3'-R	GCCTGGGTAGAAATCCGTCA
HLA-DPB1-5'-F	GCGTGTTCATGTCTGCATCC
HLA-DPB1-5'-R	TGATCCTCAGAGCCTGGACA
HLA-DPB1-XYbox-F	GTCCAATCCCAGGGTCACAG
HLA-DPB1-XYbox-R	TGAAAAGAGCTGCAGTCAGGA

XYS box Cloning Primers	
BglII-XYbox-DRB1-prox-F	GGAGATCTCTTGTGAACACATCACTGAC
HindIII-XYbox-DRB1-prox-R	CAAAGCTTGCTGGAGAACAGGACAGGAC
MluI-XYbox-DRB1-distal-F	CCACGCGTCACATTTTGCTTTACTCTGC
XhoI-XYbox-DRB1-distal-R	TACTCGAGCAGATGTGCATTAGAACTGG
5xGal4-UAS-F	TCGAGCGGAGTACTGTCCTCCGAGCGGAGTACTGTCCTCCGAGCGGAGTACTGTCCTCCGAGCGGAGTACTGTCCTCCGAGCGGAGTACTGTCCTCCGAGAGA
5xGal4-UAS-R	GATCTCTCGGAGGACAGTACTCCGCTCGGAGGACAGTACTCCGCTCGGAGGACAGTACTCCGCTCGGAGGACAGTACTCCGCTCGGAGGACAGTACTCCGC

miRNA Primers	
miRE-Xho-fw	TGAACTCGAGAAGGTATATTGCTGTTGACAGTGAGCG
miRE-EcoOligo-rev	TCTCGAATTCTAGCCCCTTGAAGTCCGAGGCAGTAGGC

miR-CNOT1-template	TGCTGTTGACAGTGAGCGcggcttccaagatatagcaataTAGTGAAGCCACAGATGTAtattgctatatcttggaagccaTGCCTACTGCCTCGGA
miR-CNOT2-template	TGCTGTTGACAGTGAGCGacatctggaatatgacaaattaTAGTGAAGCCACAGATGTAtaatttgtcatattccagatggTGCCTACTGCCTCGGA
miR-CNOT3-template	TGCTGTTGACAGTGAGCGatgacctaaagaaggagattaaTAGTGAAGCCACAGATGTAttaatctccttctttaggtcagTGCCTACTGCCTCGGA
miR-Luci-template	TGCTGTTGACAGTGAGCGcccgcctgaagtctctgattaaTAGTGAAGCCACAGATGTAttaatcagagacttcaggcgggTGCCTACTGCCTCGGA
